# Short- and long-term impacts of the National Essential Medicines Policy on drug availability, price, and usage in a deprived rural county in southwestern China: an interrupted time series analysis across 8 years

**DOI:** 10.3389/fpubh.2024.1355239

**Published:** 2024-08-29

**Authors:** Xuechen Xiong, Zhaohua Huo, Shuai Zhou, Ge Bai, Shiying He, Yinan Zhou, Jing Jia, Jianchao Quan, Li Luo

**Affiliations:** ^1^School of Public Health, Fudan University, Shanghai, China; ^2^School of Public Health, The University of Hong Kong, Hong Kong, Hong Kong SAR, China; ^3^Department of Psychiatry, Faculty of Medicine, The Chinese University of Hong Kong, Hong Kong, Hong Kong SAR, China; ^4^Ruijin Hospital, Shanghai Jiao Tong University School of Medicine, Shanghai, China; ^5^JC School of Public Health, The Chinese University of Hong Kong, Hong Kong, Hong Kong SAR, China

**Keywords:** essential medicine, drug price, centralized procurement, rural, primary care

## Abstract

**Background:**

China’s National Essential Medicines Policy (NEMP) has been implemented for over 15 years; yet empirical evidence on its long-term impacts is lacking, particularly in remote and rural regions. This study aims to assess the short-and long-term effects of NEMP on the drug availability, price, and usage in a deprived rural county in southwestern China.

**Methods:**

A quasi-experimental design was employed, featuring a single-group pre-and-post comparison. We gathered 74,436 procurement records spanning from 2009 to 2016 from the drug warehouses of local medical institutions. Pharmaceutical data were analyzed quarterly, considering various policy and therapeutic attributes. Fisher’s Drug Price Index (DPI-F) was calibrated for the retail and wholesale prices of a consistent collection of 405 medications. We conducted interrupted time-series analysis to examine the immediate and enduring impacts of NEMP’s initial (commencing in January 2011) and second (starting from December 2015) stages.

**Results:**

After initiation of NEMP, the number of available essential medicines surged by 115 but subsequently faced a steady quarterly decline (−9.1) in township healthcare centers (THCs, primary care). Conversely, county hospitals (secondary care) initially saw a reduction of 40 in drug availability but later exhibited a steady increase (+4.2 per quarter) up to the second-stage NEMP. Regarding price, THCs encountered abrupt (−26.1%/−15.9% in retail/wholesale price) and sustained (−0.2%/−0.3% per quarter) price drops after NEMP. The immediate price change after NEMP in county hospitals were milder but significant in non-essential medicines, and long-term declines were also observed in all drugs. As for total sales, a significant long-term disparity emerged between THCs (+0.9% per quarter) and county hospitals (+3.3% per quarter). Following the second-stage NEMP, retail prices in county hospitals further decreased, although wholesale prices did not; however, following price upward trends were observed in both THCs and county hospitals. Lastly, the influences of NEMP varied across different therapeutical categories of medicines.

**Conclusion:**

NEMP has successfully regulated drug prices in primary and secondary healthcare facilities in remote and rural areas, both short-term and long-term. However, a remarkable disparity in medicine availability and utilization was observed between different levels of facilities over time. Continuous monitoring is essential, with increased attention needed on the uneven impacts of the policy on diverse drugs, facilities, regions, and demographics.

## Background

Access to safe, effective and affordable medicines is pivotal in improving health outcomes and achieving universal health coverage (1). In developing and resource-limited economies, low accessibility, availability, and utilization of medicines can be consistent and stemming from inadequate drug development, production and distribution, elevated prices and costs, along with poor information, prescription, and adherence practice (2, 3). Globally, the demands for accessible, available, and affordable medicines are also intensifying, due to the inevitably aging population, prevailing non-communicable diseases, and rapidly growing health needs and drug innovations (4–6). Since the introduction of essential medicines by the World Health Organization (WHO) in 1975, Essential Medicines Lists (EML) and related policies have been widely adopted as a powerful and cost-effective tool to promote population health and health equity in different countries (7, 8).

Over the past 15 years (2008–2022), China has witnessed a sixfold increase in *per capita*l health spending, from US$117 (1 USD = 7.31 CNY) to US$843, with an annual growth rate of 16.4% (9). Despite a pullback after health system reforms, pharmaceutical expenses still count for a substantial 33% of China’s soaring health expenditure in China in 2018, significantly exceeding the OECD average of 17% (10, 11). Factors contributing to China’s high drug expenditures include a fragmented distribution system, lax regulation on advertising and promotions, distorted compensation mechanisms, and irrational medicine use (12, 13). The increases in both medicine and health spending also raise concerns about the accessibility and financial sustainability of health system.

In 2009, China launched the National Essential Medicines Policy (NEMP) as a cornerstone of healthcare system reform, aiming to ensure universal health coverage for all citizens (14). NEMP’s objectives are to enhance the availability, affordability, quality and rational use of medicines through the establishment of National Essential Medicines Lists (NEML), centralized tendering, procurement, distribution, and enforcement of essential medicine prescriptions and usage under zero-markup regulations (14, 15). The initial three-year implementation of NEMP in primary healthcare facilities (PHFs) has yielded positive outcomes, including declined drug prices and medical expenditure (16), increased access to medicines and basic health services (17), diminished financial incentives from drug sales for healthcare providers (14), and more rational medicine usage (18, 19).

While the NEMP in China has made strides, it faces significant hurdles. These include a scarcity of low-price and pediatric medicines (20–22), polypharmacy and the over-prescription of antibiotics and injections (18, 23). Primary care services under NEMP are also grappling with discontinuation, operational challenges, and financial constraints (24, 25). Particularly in the western, rural, and impoverished areas of China, these issues are more acute, due to the lower affordability and resources available to local residents and healthcare systems (25–27). Such policy shortcomings can be interpreted by the dynamic responses of stakeholders to the top-down implementation of NEMP within a complex adaptive system (28). For instant, suppliers might suspend the supply of medicines to rural and fragmented markets under low profits or even a financial loss; clinical practitioners might resort to inappropriate prescribing practice, due to insufficient information, fewer choices and altered financial incentives; and patients might changing their healthcare-seeking behavior, opting for higher-level facilities instead (25, 29, 30).

Besides the above challenges, NEMP in China requires further investigation as follows. First, the long-term impacts of NEMP vary from the short-term outcomes and exhibit considerable regional variations. Recent reviews have shown that despite a long-term decrease in median price ratios (31), the availability of essential medicines has not improve and remains low after a decade of NEMP implementation (32). This issue is even more pronounced in some western and rural areas, where the accessibility and affordability challenges have persisted or worsened (31–34). Given that, there is a necessity for more comprehensive and consistent long-term surveillance data from these areas (32).

Second, while NEMP has primarily focused on the primary care system, its spill-over effects on higher levels of facilities cannot be overlooked. Especially, several regulations have been expanded to encompass a broader scope over time. Studies from eastern and economically developed provinces reveal that the availability of essential medicines at secondary and tertiary care levels is significantly lower than at primary care facilities, suggesting a policy gap at these levels (35, 36). However, evidence from western and underdeveloped regions remain sparse.

Third, the implementation of NEMP may exert disproportionate impacts across various medicine categories. Notably, the availability of systematic hormonal preparations and nervous system medications was lower than other types, while the affordability of antineoplastic and immunomodulating agents remained less than optimal (31, 32). Prior research has often been limited to a narrow selection of medicines, leaving a gap in the understanding of effects on none-NEML pharmaceuticals and traditional Chinese medicines (TCMs).

Finally, while median price ratio—a comparison of local prices to median international reference prices—is a widely accepted method developed by WHO/HAI for evaluating drug prices (37), it relies heavily on the availability of international data and may not accurately represent a broad spectrum of medicines. It also fails to capture continuous fluctuations in drug prices and usage. Drug price index (DPI), which measures the ratio of prices of a fixed basket of goods between different periods, offers a viable alternative to address these shortcomings, and is increasingly used in drug price studies (16, 38, 39).

This study aims to evaluate both the short-and long-term impacts of NEMP on the drug availability, price and usage within a deprived rural county in southwestern China, covering the years 2009 to 2016. We further explore the varying impacts of NEMP across different healthcare facility levels, price types, and medicine classifications.

## Methods

### Research questions and hypotheses

Question 1: What are the short-and long-term impacts of NEMP on drug availability, price, and usage in a deprived rural county in southwestern China?

*Hypothesis* 1: NEMP has enhanced the availability, lowered prices and increased usage of essential medicines shortly after implementation.

*Hypothesis* 2: The initial benefits of NEMP has maintained over the long term.

Question 2: Do the impacts of NEMP remain uniform across PHFs and secondary care hospitals?

*Hypothesis* 3: NEMP has spillover impacts on the availability, prices and usage of medicines in higher-tier healthcare facilities.

Question 3: Are the impacts of NEMP consistent across different categories of medicines?

*Hypothesis* 4: Impacts of NEMP vary among different therapeutic classes of medicine.

*Hypothesis* 5: Impacts of NEMP differ between western medicines and TCMs.

*Hypothesis* 6: NEMP has spillover effects on the availability, prices and usage of non-essential medicines.

Question 4: Does selection of different price indicators affect the perceived impacts of NEMP on drug prices?

*Hypothesis* 7: Impacts of NEMP on retail prices are more pronounced than on wholesale prices.

*Hypothesis* 8: The Fisher Price Index reveals a greater price reduction of NEMP compared to the Laspeyres Price Index.

### Study design, setting and policy

This study employed a quasi-experimental design to compare conditions before and after the implementation of NEMP in a rural, remote and poverty-stricken county in Yunnan Province, southwestern China. Yunnan province is characterized by its mountainous and plateau geography, and its residents are facing limited access to healthcare services. The county in selection is predominantly mountainous (94%); three-quarters of its population living in rural areas; its per capital gross domestic product per capita was only US$4,788 in 2020, 70% of the provincial average and 53% of the national average (40). In this economically and geographically isolated county, the three-tiered public healthcare system (73 village clinics – 7 township healthcare centers [THCs]—three county hospitals) severs as a primary healthcare provider for its residents. The county’s geographic and functional isolation makes it an exemplary site to study the impacts of NEMP as a complex social intervention at a micro-level.

NEMP’s rollout in the selected county occurred in two stages (Appendix 1). The initial stage policies (2010.9–2015.10) targeted all government-owned PHFs, including THCs and village clinics. This phase introduced centralized procurement, mandatory prescription of essential medicines, zero-markup retail pricing policy, and conditional financial support. The second stage started from November 2015, expanding to include secondary care facilities (county hospitals) with similar measures: centralized procurement, obligatory use and zero-markup price in essential drugs.

### Data collection

Data were collected from two THCs and two government-operated county hospitals. The THCs, situated at the heart of two distinct townships, deliver primary care for 39.1% of the county’s population across 29.7% of its land. The county hospitals, as the highest-tier local medical institutions, provide secondary care for the entire population and guidance to all PHFs. Drug procurement records between January 2009 and December 2016 were compiled from both electronic systems or paper records in the pharmaceutical warehouses of these facilities.

**Figure 1:**
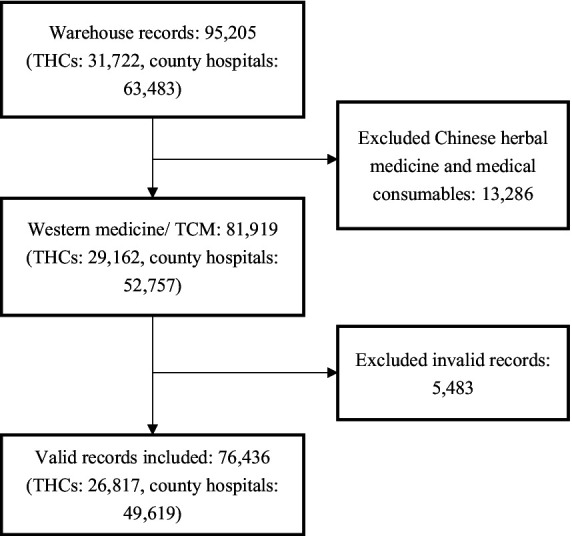
Flow chart of records selection.

We presumed that all stocked medicines were ultimately dispensed to patients, equating drug purchases with drug utilization. Each procurement record contained details such as the purchase date, generic name, dosage form, specification, quantity, and both wholesale/manufacturer (i.e., medicines sold from manufacturers and distributors to hospitals) and retail/consumer prices (i.e., medicines prescribed from hospitals and doctors to patients). Hard-copy records were transcribed by two bachelor medical students and cross-validated by two research members independently (Z.H. and Z.S). Out of 95,205 purchase records, 76,436 were deemed suitable for analysis after discarding those of herbal medicine, medical consumables and invalid information (unmatched drug, extreme value, withdrawal orders; see Figure 1). The total sales from the analyzed records amounted to US$23.6 million, with 93.6% originated from county hospitals and 6.4% from THCs.

### Drug categorization

Medicines were categorized by their policy properties (essential or non-essential drug; western or TCMs) and therapeutical attributes (Anatomical Therapeutic Chemical, ATC system) (41). The dataset encompassed 644 chemical and biological substances and 508 TCMs (see Appendices 2, 3). For standardized analysis of quantity and price, a “unique” medicine was defined by its generic name, dosage form, and specification (e.g., Abacavir-Oral-0.3 g). Different specifications of the same generic name and dosage would be converted to the minimum unit (e.g., Abacvir-Oral-0.6 g - > Abacvir-Oral-0.3 g*2). In total, 797 unique western medicines (520 essential, 422 non-essential) and 599 unique TCMs (272 essential, 327 non-essential) were identified.

### Outcome measures

The availability of medicines was quantified by their presence in healthcare facilities. Drug usage was assessed by sales value. Defined daily doses (DDDs) were not used because over a half (52%) of western medicines and all TCMs did not correspond to a valid DDD value in the ATC system (41).

Regarding drug prices, both wholesale/manufacturer (medicines sold to hospitals) and retail/consumer prices (medicines prescribed to patients) were analyzed, for NEMP aimed to cut costs at these supply chain stages. Prices were tracked by the drug price index (DPI), an indicator of price ratios of a fixed basket of goods between the reporting period and the baseline period (38). Three commonly used DPIs were calibrated: Laspeyres (DPI-L), Paasche (DPI-P), and Fisher (DPI-F) (16, 42). In our analysis, DPIs were calculated for a fixed basket of 405 (29.0% among all 1,396) medicines with complete records from 2009 to 2016.

DPI-L measures price ratios over different periods, weighted by baseline consumption quantity ( $L_{p}=\sum P_{1}Q_{0}/\sum P_{0}Q_{0}$ ; where P_0_ and P_1_ denote prices of a good in baseline and reporting periods, and Q_0_ and Q_1_ denote the consumption quantities). It assumes constant consumption over time and reflects the complete price changes. Conversely, DPI-P is weighted by the consumption quantity in reporting periods ( $P_{p}=\sum P_{1}Q_{1}/\sum P_{0}Q_{1}$ ), assuming quantity changes follow after price changes. A DPI > 1 indicates increase costs to acquire the same basket of goods, < 1 indicates a decreased costs, and = 1 indicates stable costs.

Both DPI-L and DPI-P are biased due to their assumption of unchanged consumption. DPI-F addresses this by averaging changes in baseline and reporting periods using the geometric mean of DPI-L and DPI-P ( $F_{p}=\sqrt{L_{p}\times P_{p}}$ ) (43). Comparing DPI-L, DPI-P, and DPI-F further reflects relative consumption changes in different drugs, where DPI-F < DPI-L (or DPI-F > DPI-P) suggests increased use of low-price drugs, and vice versa.

### Statistical analysis

Considering a policy lag-effect of 3 months, as determined by expert consultation and data observation, we established two intervention points (January 2011 and January 2016), and three policy periods (pre-implementation: January 2009–December 2010; first-stage implementation: January 2011—December 2015; and second-stage implementation: January 2016—December 2016). The analytical interval was set at 3 months, aligning with the purchase cycle of PHCs. The quarterly observation interval is also appropriate and stable to capture secular changes in drug price and usage at the county level. In total, 32 observation points were identified over the eight-year period.

Descriptive analysis was performed on the number, sales, retail price and wholesale price of medicines, categorized by medicine types and facility levels of facilities (i.e., THCs, county hospitals, and overall facilities). Single-group interrupted time-series analysis (ITSA) was utilized to examine the immediate and sustained impacts of NEMP on these metrics (44–46). In ITSA, drug sales were log-transformed, and time interval was set quarterly, totaling 32 time points from 2009–2016: 8 for pre-NEMP, 20 for first stage NEMP, and 4 for second stage NEMP. Segmented linear regression models were constructed with two interruption points: $Y_{t}=\beta_{0}+\beta_{1}T+\beta_{2}X_{1\_t}+\beta_{3}TX_{1\_t}+\beta_{4}X_{2\_t}+\beta_{5}TX_{2\_t}+\varepsilon_{t}$ , where Y_t_ denotes the outcome in quarter t, T denotes the time point since observation, and X_1_t_ and X_2_t_ denote the implementation of first-stage and second-stage policies at T (coded 0 or 1). Coefficient β_0_ and β_1_ separately estimate the baseline level and trend of the outcome in pre-policy periods; β_2_ and β_3_ separately estimate changes in immediate level and long-term trend after the first-stage policy; and β_4_ and β_5_ separately estimates changes in level and trend after the second-stage policy. Ε_t_ measures the random error at time T. Autocorrelation in time-serial data was tested by Durbin–Watson statistics and adjusted with Prais-Winsten method (47).

In sensitivity analysis, inflation was accounted for in both retail (using consumer price index, CPI) and wholesale prices (using producer price index, PPI). All analyses were performed in R version 4.1, and a *p*-value <0.05 was considered statistically significant.

## Results

### Descriptive analysis

Over 8 years, the number of available medicines in the selected county increased modestly from 719 to 782 (+8.8%), while the DPI-F declined by 8.9% and the total medicine spending surged fivefold, from US$0.2 million to US$1.0 million (Appendix 4). By policy attributes, sales of essential medicines grew significantly more than non-essential drugs (5.4-fold vs. 3.0-fold); however, the price cut on essential medicines was less pronounced than on non-essential drugs, with DPI-F decreases of 1.9% versus 24.0%, respectively. By facility levels, county hospitals experienced a 26.4% increase in medicine numbers, a 383.5% surge in sales, and an 8.2% reduction in prices over the same period. Conversely, THCs saw a 46.2% decrease in medicine numbers, a 93.9% increase in sales, and a slight 2.1% price increase (Appendix 4).

### Availability of medicines

**Figure 2:**
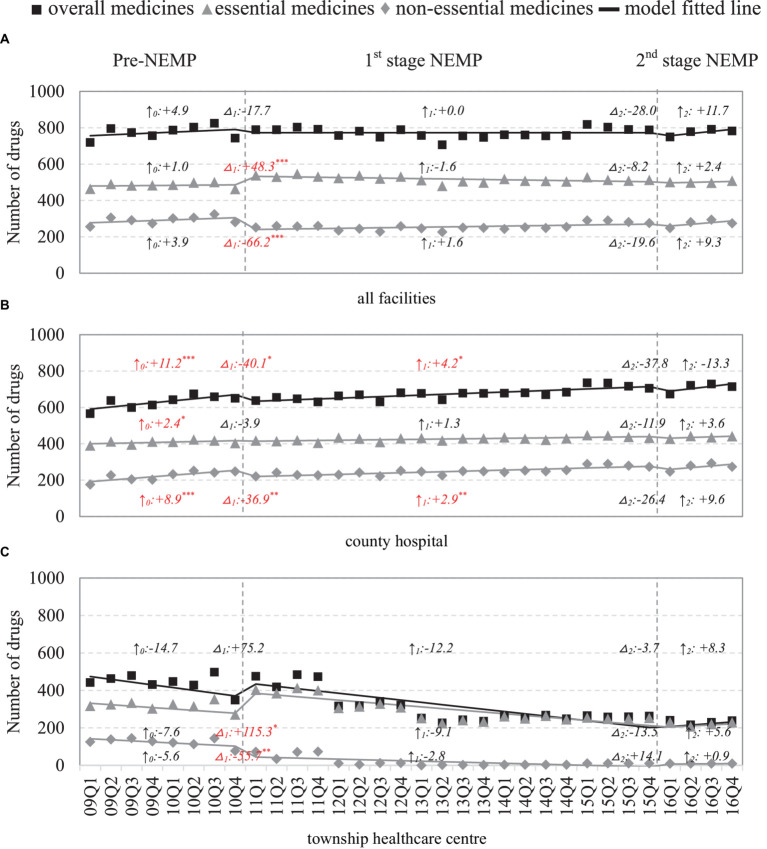
Number of medicines by facility level and essential medicine list. △_1_: immediate change after first-phase NEMP (estimator β_3_ in ITSA model); ↑_0_: sustained change per observation period before first-phase NEMP (β_1_); ↑_1_: sustained change per observation period after first-phase NEMP (β_1_ + β_3_); △_2_: immediate change after second-phase NEMP (β_4_); ↑_2_: sustained change per observation period after second-phase NEMP (β_1_ + β_3_ + β_5_); ITSA, interrupted time-series analysis; NEMP, National Essential Medicines Policy; **(A)** all facilities, **(B)** county hospital, **(C)** township healthcare center. *p < 0.05; **p < 0.01; ***p < 0.001.

Across the county, the number of available medicines remained steady at around 800 across 8 years, yet displaying a disparity between essential and non-essential medicines (Figure 2A). The number of essential medicines increased immediately after first-stage NEMP (ITSA estimate: +48 or 10.4%, *p* < 0.001), while the number of non-essential medicines decreased dramatically (−66 or − 23.6%, *p* < 0.001). No significant secular trend changes were detected in the number of either medicine types; however, the number of non-essential drugs underwent a brief dip after the second stage of NEMP (−20 or − 7.3%, *p* > 0.05).

Facility-wide, county hospitals noted a sharp drop in non-essential drugs (−37 or − 15.0%, *p* < 0.01) after first stage NEMP, followed by a deceleration in growth rate (+2.9 per period, *p* < 0.01; Figure 2B). In THCs, there was a marked increase in essential drugs (115 or 42.4%, *p* < 0.05) and an exit of non-essential drugs (−56 or 72.7%, *p* < 0.01) post the first stage NEMP (Figure 2C). Despite it, essential drugs in THCs continued to decline longitudinally, failing to recover even after the second-stage NEMP.

By medicine types, NEMP consistently influenced the availability of both western medicines and TCMs (Appendix 5). By ATC-classifications, most medicine categories in county hospitals initially dropped but then increased following the first-stage NEMP, except for genitourinary system and sex hormones drugs, which showed a sustained long-term decrease (Appendices 6, 7). In THCs, despite initial increases in most medicine categories after the first-stage NEMP, a long-term downward trend was common and persisted beyond the second-stage NEMP (Appendices 6, 7).

### Sales of medicines

**Figure 3:**
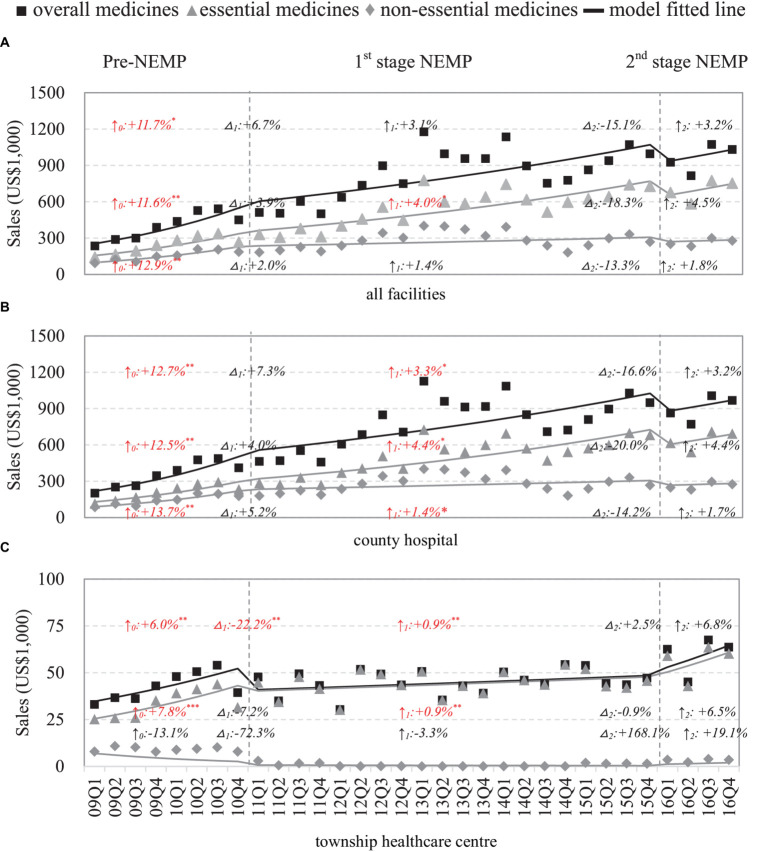
Sales of medicines by facility level and essential medicine list. △_1_: immediate change% after first-phase NEMP [exp(β_3_) in ITSA model]; ↑_0_: sustained change per observation period before first-phase NEMP (β_1_); ↑_1_: sustained change% per observation period after first-phase NEMP [exp(β_1_ + β_3_)]; △_2_: immediate change% after second-phase NEMP [exp(β_4_)]; ↑_2_: sustained change% per observation period after second-phase NEMP [exp(β_1_ + β_3_ + β_5_)]; ITSA, interrupted time-series analysis; NEMP, National Essential Medicines Policy; **(A)** all facilities, **(B)** county hospital, **(C)** township healthcare center. *p < 0.05; **p < 0.01; ***p < 0.001.

County-wide drug expenses were presented (Figure 3A). Drug expenses of county hospitals were a major driver in the pharmaceutical market, with increasing trends for both essential (ITSA estimate: +4.4% per quarter) and non-essential drugs (+1.4% per quarter) after first-stage NEMP (Figure 3B). However, following the second-stage NEMP, county hospitals encountered a 14.2–20.0% drop in drug expenditure of essential and non-essential drugs. In THCs, drug expenditure dropped significantly by 22.2% (essential: −7.2%; non-essential: −72.3%) yet kept increasing at a modest rate (+0.9% per quarter) after policy implementation (Figure 3C).

By medicine types, sales dynamics differed between western medicines (rebound after an initial drop) and TCMs (stable after a sharp rise) in THCs following NEMP (Appendix 8). Further by ATC-classifications, most medicine categories in county hospitals exhibited rising sales trends, except for (i) Genitourinary system and sex hormones, (ii) anti-infectives, (iii) antiparasitic, and (iv) TCMs of surgery, gynecology and orthopedics (Appendices 9, 10). In THCs, after the first-stage NEMP, rises in levels or secular trends were observed mainly in (i) systemic hormonal preparations, (ii) respiratory system and (iii) TCM for internal medicine (Appendices 9, 10).

### Retail price of medicines

**Figure 4:**
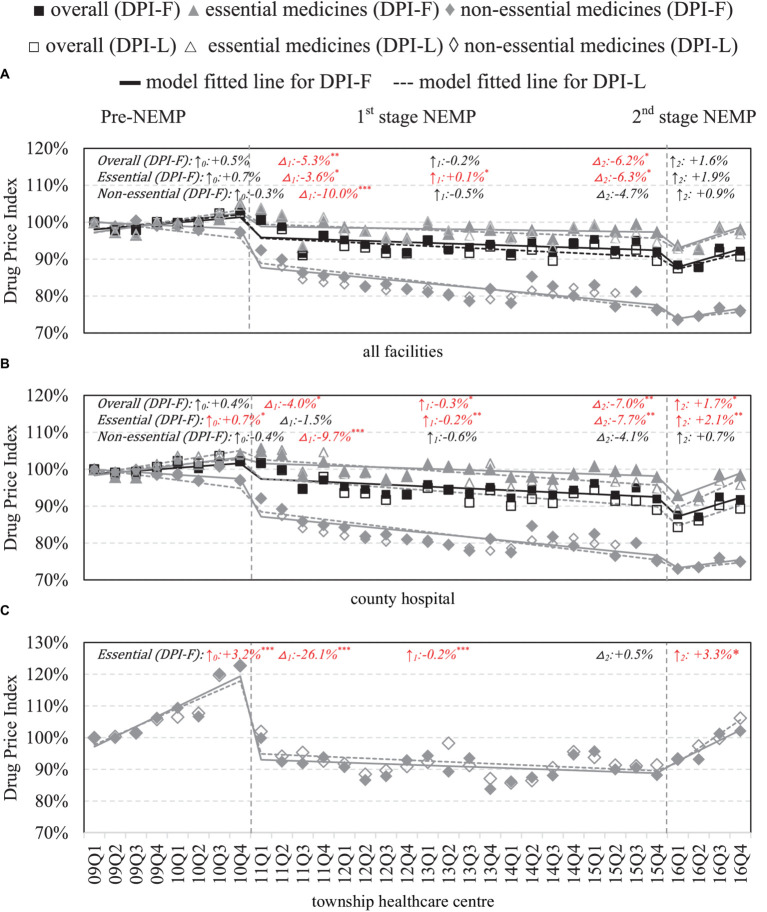
Retail price of medicines by facility level and essential medicine list. △_1_: immediate price change% after first-phase NEMP [exp(β_3_) in ITSA model]; ↑_0_: sustained change per observation period before first-phase NEMP (β_1_); ↑_1_: sustained price change% per observation period after first-phase NEMP [exp(β_1_ + β_3_)]; △_2_: immediate price change% after second-phase NEMP [exp(β_4_)]; ↑_2_: sustained price change% per observation period after second-phase NEMP [exp(β_1_ + β_3_ + β_5_)]; ITSA, interrupted time-series analysis; NEMP, National Essential Medicines Policy; **(A)** all facilities, **(B)** county hospital, **(C)** township healthcare center. *p < 0.05; **p < 0.01; ***p < 0.001.

County-wide, immediate drops in DPIs were observed in both essential (ITSA estimates: first-stage −3.6%, *p* < 0.05; second-stage −6.3%, *p* < 0.05) and non-essential medicines (first-stage: -10.0% *p* < 0.001, second-stage: −4.7% *p* > 0.05) following the two stages of NEMP (Figure 4A). However, upward price trends were observed subsequently after the second-stage NEMP. By facility levels, in county hospitals, impacts of NEMP on prices mirrored the county-wide effects for both essential and non-essential medicines (Figure 4B). In THCs, a sharp decrease (−26.1%, *p* < 0.001) and a downward trend (−0.2% per quarter) were noted after the first-stage NEMP; however, prices began to climb after second stage NEMP (+3.3% per quarter, *p* < 0.05; Figure 4C).

By medicine types, in contrast to western medicines, retail prices of TCMs in county hospitals surged immediately after the first-stage NEMP (+4.4%, *p* < 0.05). Price cut in TCMs were also not as steep as western medicines in THCs (−15.3%, *p* < 0.01) following first-stage NEMP (Appendix 11). Further by ATC classifications, reverse price increases after NEMP were observed in county hospitals in (i) dermatological, (ii) genitourinary system and sex hormones, (iii) systemic hormonal preparations, (iv) Musculo-skeletal system, (v) antiparasitic, (vi) sensory organs and (vii) TCMs of surgical and orthopedic (Appendices 12, 13). In THCs, all medicines exhibited price declines post NEMP, except for (i) Musculo-skeletal and (ii) sensory organ drugs. Upward price trends were observed in various medicines after the second-stage NEMP, despite non-significant levels.

Comparative analyses using Fisher and Laspeyres indices showed similar results (Figure 4A–C), except for higher DPI-F in TCMs within county hospitals, suggesting a higher prescription rate of high-price TCMs (Appendix 11).

### Wholesale price of medicines

**Figure 5:**
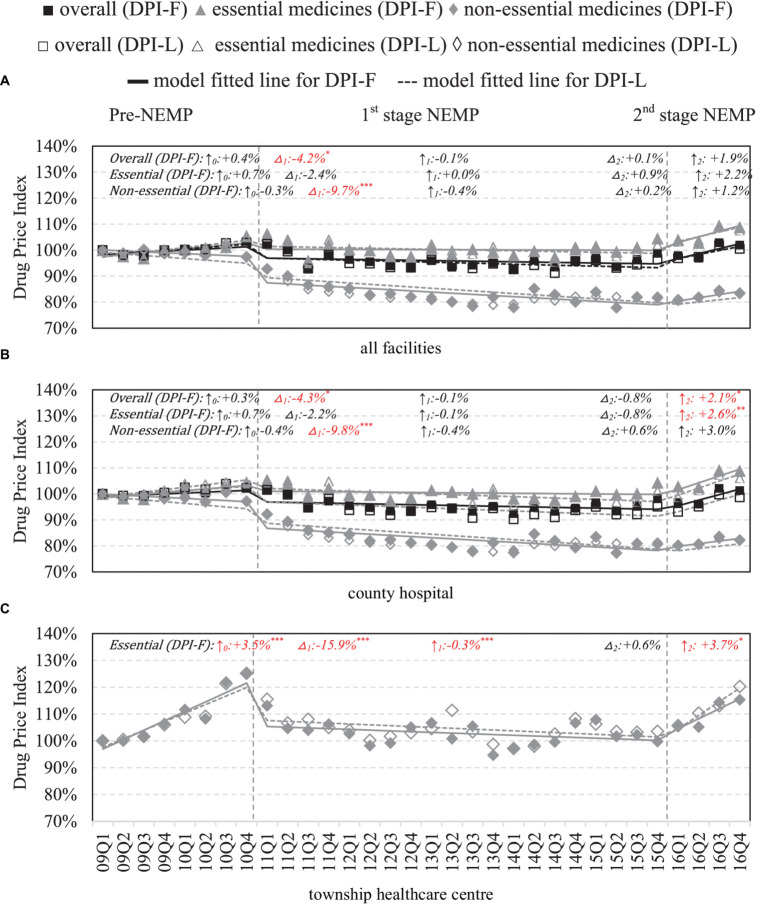
Wholesale price of medicines by facility level and essential medicine list. △_1_: immediate price change% after first-phase NEMP [exp(β_3_) in ITSA model]; ↑_0_: sustained change per observation period before first-phase NEMP (β_1_); ↑_1_: sustained price change% per observation period after first-phase NEMP [exp(β_1_ + β_3_)]; △_2_: immediate price change% after second-phase NEMP [exp(β_4_)]; ↑_2_: sustained price change% per observation period after second-phase NEMP [exp(β_1_ + β_3_ + β_5_)]; ITSA, interrupted time-series analysis; NEMP, National Essential Medicines Policy; **(A)** all facilities, **(B)** county hospital, **(C)** township healthcare center. *p < 0.05; **p < 0.01; ***p < 0.001.

County-wide, significant drops in wholesale prices were mainly observed in non-essential medicines (−9.7%, *p* < 0.001) after first-stage NEMP (Figure 5A). By facilities, in county hospitals, the first-stage NEMP brought a significant decrease in non-essential drug price (−9.8%, *p* < 0.001) (Figure 5B). In contrast, after the second-stage NEMP, essential drugs exhibited a significant upward trend (+2.6% per quarter, *p* < 0.01). In THCs, a sharp decline (−15.9%, *p* < 0.001) and a continued downward trend (−0.3% per quarter, *p* < 0.001) were observed in essential drug price after the first-stage NEMP. However, the trend reversed with an increase in prices after the second stage (+3.7%, *p* < 0.05; Figure 5C).

By medicine types, TCMs saw increases in wholesale price after the first-stage and second-stage NEMP (Appendix 14). Further by ATC-classifications, NEMP’s impacts on wholesale prices in both county hospitals and THCs mirrored those in retail prices (Appendices 15, 16).

### Sensitivity analysis

Main findings of adjusting retail and wholesale prices by CPI and PPI align with the base case analysis. There are only slight variations in the magnitude and statistical levels of impacts (Appendix 17).

## Discussion

### Main findings

The NEMP in China, a comprehensive and enduring medical reform, was evaluated in a deprived rural county in southwestern China. Our summary of findings (Table 1) reveals that NEMP initially improved the availability of essential drugs and curtailed the use of non-essential drugs in primary care facilities, in line with NEMP’s establishment and mandatory implementation. Unexpectedly, we observed a notable spillover effect where the number of non-essential drugs decreased in county hospitals (secondary care) after NEMP, possibly due to the substitution between drug types and new regulations on purchasing platforms. Nevertheless, consistent with previous evidence (31–34), we found the long-term availability of (essential) medicines deteriorated in the sample county, especially in primary care centers. This has become one of the nonnegligible concerns by the public (48), and could seriously hamper the sustainability of NEMP. Key obstacles to access essential medicines in undeveloped and remote areas are still to be overcome, including high transportation costs, low supplier profits, inadequate storage in PHFs, and a lack of motivation and confidence to provide medical or pharmaceutical services among primary care practitioners (24, 49).

**Table 1:** Summary of findings (interrupted time series analysis).

ITSA estimators by quarter (95% Confidence Interval, *p*-value)	Pre-NEMP (2009.1–2010.10)		First-stage NEMP (2010.11–2015.10)		Second-stage NEMP (2015.11–2016.12)	
	Initial Level (β1)	Initial Trend (β2)	Change in Level (β3)	Change in Trend (β4)	Change in Level (β5)	Change in Trend (β6)
Availability of medicines	(number)	(number)	(number)	(number)	(number)	(number)
County-wide	757 (718, 795), <0.001	4.9 (−4.2, 13.9), 0.301	−17.7 (−64.2, 28.9), 0.464	−4.9 (−14.3, 4.5), 0.318	−28.0 (−101.1, 45.1), 0.459	11.7 (−13.6, 37.0), 0.373
Essential medicines	479 (461, 498), <0.001	1.0 (−3.4, 5.3), 0.669	48.3 (26.0, 70.5), <0.001	−2.5 (−7.0, 2.0), 0.284	−8.2 (−42.9, 26.5), 0.648	4.0 (−8.0, 16.0), 0.521
Non-essential medicines	277 (254, 300), <0.001	3.9 (−1.5, 9.4), 0.170	−66.2 (−94.3, −38.1), <0.001	−2.4 (−8.0, 3.3), 0.417	−19.6 (−64.2, 25.1), 0.398	7.7 (−7.7, 23.2), 0.336
County hospitals	591 (568, 614), <0.001	11.2 (5.6, 16.8), <0.001	−40.1 (−68.9, −11.4), 0.011	−7.0 (−12.7, −1.3), 0.024	−37.8 (−86.6, 10.9), 0.140	9.0 (−7.9, 25.9), 0.305
Essential medicines	400 (391, 409), <0.001	2.4 (0.3, 4.6), 0.036	−3.9 (−15.0, 7.2), 0.493	−1.1 (−3.3, 1.1), 0.322	−11.9 (−31.3, 7.6), 0.244	2.3 (−4.5, 9.1), 0.517
Non-essential medicines	191 (175, 208), <0.001	8.9 (4.9, 12.8), <0.001	−36.9 (−57.1, −16.6), 0.001	−5.9 (−10.0, −1.9), 0.008	−26.4 (−60.2, 7.4), 0.138	6.7 (−5.0, 18.4), 0.274
THCs	474 (384, 564), <0.001	−14.7 (−34.2, 4.7), 0.150	75.2 (−18.3, 168.6), 0.127	2.5 (−18.9, 24.0), 0.818	−3.7 (−127.3, 120.0), 0.954	20.5 (−25.6, 66.6), 0.392
Essential medicines	331 (268, 394), <0.001	−7.6 (−20.6, 5.5), 0.265	115.3 (55.6, 174.9), <0.001	−1.5 (−16.2, 13.2), 0.843	−13.5 (−89.4, 62.4), 0.730	14.7 (−14.9, 44.3), 0.341
Non-essential medicines	142 (115, 168), <0.001	−5.6 (−11.8, 0.7), 0.092	−55.7 (−87.8, −23.6), 0.002	2.8 (−3.7, 9.4), 0.402	14.1 (−35.5, 63.7), 0.581	3.6 (−13.6, 20.8), 0.684
Retail price	(DPI-F)	(DPI-F)	(DPI-F)	(DPI-F)	(DPI-F)	(DPI-F)
County-wide	98.0 (95.4, 100.6), <0.001	0.5 (−0.1, 1.1), 0.146	−5.3 (−8.5, −2.1), 0.003	−0.7 (−1.3, 0.0), 0.055	−6.2 (−11.4, −1.0), 0.027	1.8 (0.0, 3.6), 0.058
Essential medicines	97.3 (94.5, 100.0), <0.001	0.7 (0.1, 1.4), 0.035	−3.6 (−6.9, −0.2), 0.048	−0.8 (−1.5, −0.2), 0.024	−6.3 (−11.9, −0.7), 0.036	2.0 (0.1, 3.9), 0.053
Non-essential medicines	100.0 (96.8, 103.1), <0.001	−0.3 (−1.0, 0.5), 0.512	−10.0 (−13.9, −6.1), <0.001	−0.3 (−1.1, 0.5), 0.498	−4.7 (−10.8, 1.5), 0.147	1.5 (−0.7, 3.6), 0.188
County hospitals	98.7 (96.3, 101.1), <0.001	0.4 (−0.2, 1.0), 0.171	−4.0 (−6.9, −1.0), 0.014	−0.7 (−1.3, −0.1), 0.037	−7.0 (−11.6, −2.4), 0.006	2.0 (0.4, 3.6), 0.023
Essential medicines	98.3 (95.8, 100.8), <0.001	0.7 (0.1, 1.3), 0.026	−1.5 (−4.4, 1.5), 0.346	−0.9 (−1.5, −0.3), 0.008	−7.7 (−12.3, −3.1), 0.003	2.3 (0.7, 3.9), 0.009
Non-essential medicines	99.9 (96.6, 103.2), <0.001	−0.4 (−1.1, 0.4), 0.385	−9.7 (−13.8, −5.7), <0.001	−0.2 (−1.0, 0.6), 0.645	−4.1 (−10.4, 2.2), 0.209	1.3 (−0.9, 3.5), 0.257
THCs (Essential medicines)	97.1 (91.8, 102.4), <0.001	3.2 (1.9, 4.4), <0.001	−26.1 (−32.4, −19.8), <0.001	−3.4 (−4.7, −2.1), <0.001	0.5 (−9.0, 10.1), 0.913	3.5 (0.2, 6.8), 0.050
Wholesale price	(DPI-F)	(DPI-F)	(DPI-F)	(DPI-F)	(DPI-F)	(DPI-F)
County-wide	98.3 (95.1, 101.6), <0.001	0.4 (−0.3, 1.2), 0.283	−4.2 (−8.1, −0.3), 0.042	−0.5 (−1.3, 0.3), 0.194	0.1 (−5.8, 6.1), 0.969	2.0 (0.0, 4.1), 0.064
Essential medicines	97.5 (94.3, 100.8), <0.001	0.7 (0.0, 1.5), 0.067	−2.4 (−6.3, 1.6), 0.251	−0.8 (−1.6, 0.0), 0.069	0.9 (−5.4, 7.2), 0.789	2.2 (0.0, 4.3), 0.061
Non-essential medicines	100.1 (96.3, 103.8), <0.001	−0.3 (−1.2, 0.5), 0.441	−9.7 (−14.2, −5.3), <0.001	−0.1 (−1.0, 0.8), 0.840	0.2 (−6.4, 6.8), 0.948	1.7 (−0.6, 4.0), 0.168
County hospitals	99.0 (96.0, 102.0), <0.001	0.3 (−0.4, 1.0), 0.344	−4.3 (−7.8, −0.8), 0.024	−0.5 (−1.2, 0.2), 0.201	−0.8 (−6.0, 4.4), 0.775	2.3 (0.5, 4.1), 0.021
Essential medicines	98.5 (95.6, 101.4), <0.001	0.7 (0.0, 1.4), 0.061	−2.2 (−5.6, 1.3), 0.233	−0.7 (−1.5, 0.0), 0.051	−0.8 (−6.0, 4.4), 0.766	2.7 (0.9, 4.5), 0.008
Non-essential medicines	99.9 (96.1, 103.7), <0.001	−0.4 (−1.3, 0.5), 0.363	−9.8 (−14.3, −5.4), <0.001	0.0 (−1.0, 0.9), 0.941	0.6 (−6.0, 7.2), 0.860	1.5 (−0.9, 3.8), 0.227
THCs (Essential medicines)	96.9 (91.1, 102.8), <0.001	3.5 (2.2, 4.9), <0.001	−15.9 (−22.9, −8.9), <0.001	−3.8 (−5.2, −2.4), <0.001	0.6 (−9.9, 11.2), 0.905	4.0 (0.3, 7.7), 0.043
Sales of medicines	(US$1,000)	(%)	(%)	(%)	(%)	(%)
County-wide	254 (188, 342), <0.001	11.7 (4.7, 19.1), 0.002	6.7 (−21.7, 45.5), 0.683	−7.7 (−14.0, −0.9), 0.036	−15.1 (−43.7, 28.0), 0.442	0.1 (−14.1, 16.6), 0.988
Essential medicines	156 (121, 201), <0.001	11.6 (5.2, 18.3), 0.001	3.9 (−22.8, 39.7), 0.802	−6.8 (−12.4, −0.8), 0.036	−18.3 (−46.8, 25.4), 0.363	0.4 (−13.7, 16.8), 0.960
Non-essential medicines	98 (65, 147), <0.001	12.9 (3.8, 22.6), 0.009	2.0 (−29.9, 48.6), 0.917	−10.2 (−18.3, −1.2), 0.035	−13.3 (−46.1, 39.3), 0.559	0.4 (−16.8, 21.0), 0.970
County hospitals	218 (159, 300), <0.001	12.7 (5.3, 20.6), 0.002	7.3 (−22.2, 47.9), 0.673	−8.4 (−15.0, −1.2), 0.031	−16.6 (−45.3, 27.1), 0.405	−0.1 (−14.7, 17.1), 0.993
Essential medicines	129 (98, 171), <0.001	12.5 (5.7, 19.8), 0.001	4.0 (−24.2, 42.7), 0.810	−7.2 (−13.3, −0.8), 0.038	−20.0 (−48.9, 25.4), 0.340	0.0 (−14.8, 17.3), 0.999
Non-essential medicines	89 (59, 134), <0.001	13.7 (4.5, 23.7), 0.006	5.2 (−28.2, 54.0), 0.797	−10.8 (−19.0, −1.9), 0.027	−14.2 (−47.0, 38.9), 0.538	0.3 (−17.0, 21.3), 0.974
THCs	35 (30, 40), <0.001	6.0 (2.5, 9.7), 0.002	−22.2 (−34.6, −7.6), 0.008	−4.8 (−8.1, −1.5), 0.009	2.5 (−25.0, 40.0), 0.879	5.9 (−5.2, 18.1), 0.319
Essential medicines	25 (22, 30), <0.001	7.8 (3.8, 12.0), <0.001	−7.2 (−23.6, 12.7), 0.459	−6.4 (−10.0, −2.7), 0.002	−0.9 (−29.8, 39.8), 0.959	5.5 (−6.5, 19.0), 0.390
Non-essential medicines	7 (0, 123), 0.200	−13 (−48.1, 45.3), 0.597	−72.3 (−95.9, 85.5), 0.197	11.3 (−40.2, 106), 0.739	168.1 (−71.4, 2,409), 0.395	23.2 (−56.2, 246), 0.695

DPI-F, Fisher Price Index; ITSA, interrupted time series analysis; NEMP, National Essential Medicines Policy; THC, township healthcare centers.

Regarding drug prices, centralized procurement and zero-markup policies of NEMP led to a substantial decline by nearly 25% and a modest long-tern decrease in consumer price of essential medicines in THCs, aligning with prior studies (16, 31, 50). Also, in county hospitals (secondary care), we observed a modest decline as spillover effects in essential drugs after first-stage NEMP, as well as an abrupt drop of 8% after zero-markup policy in second-stage NEMP. Previous studies also revealed the availability and affordability of essential drugs are improved in secondary and tertiary hospitals (35, 36). Surprisingly as a new finding, prices of non-essential medicines also fell significantly in county hospitals, potentially due to the provincial centralized procurement for these medicines and manufacturers’ evolving pricing strategies (39). Despite these reductions, the upward price trends in all types of medicines and facilities after second-stage NEMP are noteworthy, possibly due to the price deregulations, warranting further observation (36).

As a pivotal link of medication cost, wholesale price changes from manufacturers to healthcare providers were also examined were also examined. We found that wholesale price post-NEMP showed identical trends of reduction with retail price, but with lower rates, suggesting room for additional measures to reduce procurement and distribution costs.

Concerning medicine sales, fluctuations continued after NEMP, with stark disparities between primary care and secondary care facilities. THCs saw a 22.2% drop in in total drug expenses experienced after NEMP due to the price reduction and mandatory use of essential drugs. Inversely, medicine sales in county hospitals experienced a 7.3% increase, despite the drop in prices. This indicates potentially larger usage of essential medicines in secondary care after NEMP. As a new finding, over the long term, THC drug sales exhibited only a modest increase of 0.9% quarterly, whereas drug sales in county hospitals grew at a high rate of 3.3% (even 4.4% in essential drugs). This widening gap suggests that price cut does not guarantee increased consumption in medicine (51). Except for the challenges of NEMP in rural areas listed above, patients migration from primary care to higher-level or private facilities was also an important reason of decreased medicine use, due to distrust of medicine quality and dissatisfaction with monotonous supply of generic medicines (28). These unexpected results could undermine PHFs’ role as health gatekeepers in rural and remote areas.

Lastly, NEMP’s impacts varied by medicine type. As for availability, we noted a declining number in genitourinary system and sex hormone medicines in rural and deprived areas, contrary to previous studies (32). Regarding prices, western medicines of (i) dermatological, (ii) genitourinary system and sex hormones, (iii) systemic hormonal preparations and (iv) Musculo-skeletal system, (v) antiparasitic, (vi) sensory organ, and TCMs of surgical and orthopedic were faced with upward trends and required further monitoring. Additionally, price cut in TCMs after NEMP was smaller than western medicines, suggesting opportunities for policy refinement. There was also a trend of prescribing higher-priced TCMs in county hospitals, likely influenced by the mark-up price policy and government subsidy standards based on drug sales at that moment (16). Total sales analysis revealed that the most significant increases in county hospitals were in cardiovascular, systemic hormonal, musculoskeletal, and metabolism medicines. In THCs, sales more than doubled for anti-infectives and TCMs for internal medicine, while sales for cardiovascular, sensory organs, and blood-forming organs decreased significantly, contradicting epidemiological trends and highlighting issues with polypharmacy and antibiotic overprescription (18, 23).

### Strengths and limitations

This study is among the few to examine the long-term and secular impacts of NEMP over an eight-year period in southwestern China. It specifically focuses on a micro-context within a geographically isolated and economically underdeveloped county. The deliberate selection of an isolated county and the employment of a quasi-experimental design is expected to minimize potential confounding factors, such as other drug-related policies or interactions with surrounding areas. Leveraging comprehensive pharmaceutical records from major healthcare providers, we conducted multi-dimensional analyses encompassing a variety of outcomes, facility levels, medicine types, and prices. It also enables us to understand not only the policy’s intended effects but also its spillover effects on other healthcare system components. The use of price indices and ITSA serves as robust methods to address the limitations of WHO/HAI method (e.g., restrictions on dosage forms and specifications) (31, 32), and to more accurately reflect the temporal price changes of multiple medicines in the pharmaceutical market.

Undoubtedly, our study owns limitations. First, the quasi-experimental pre-post design lacking control groups cannot fully eliminate the influence of unobserved factors. We anticipate this limitation can be mitigated by selecting a geographically isolated region with a less dynamic social and healthcare system. Second, village clinics were excluded from our analysis due to incomplete drug records before 2012. These 73 clinics, typically managed by single physicians, play a vital role as foundational units in primary care. Their exclusion constrained the comprehensiveness of our findings. Also, we did not incorporate private sectors in our analysis. Since 2014, two private hospitals have been established in the studied county. Their presence could have significantly influenced patient flow and medicine consumption in the closed market. Notably, we detected fluctuations in medicine sales in county hospitals between 2014 and 2015, which merits further investigation.

Moreover, our calculation of Drug Price Index (DPI) only considered medicines with complete records across 8 years, omitting those newly introduced or discontinued during this time. Still and all, we analyzed prices of 405 pharmaceuticals, a substantially larger number than previous studies. Finally, data were only available for 1 year after the second-stage NEMP, insufficient to confirm trend changes afterwards. Extended surveillance is necessary. Moreover, using monetary sales, rather than DDDs, may be affected by the fluctuating drug prices and not accurately reflect the actual quantity of medicine usage. We advocate for more rigor methodologies to measure and amalgamate the quantities of various medicines.

### Implication

This study illuminates the dynamic effects of the National Essential Medicines Policy (NEMP) on the local pharmaceutical system from a micro-perspective in a rural, remote, and economically challenged county in Southwestern China. Our findings offer insights in policy enhancements in similar contexts characterized by low economic development, scarce health resources, high transportation costs, and limited capacity in personnel’s, finances and services (31, 32).

First, despite establishment and mandatory implementation of NEML, the availability of essential medicines remained scarce in rural, remote and underdeveloped regions, especially in primary care settings. Continuous efforts are needed on (i) financial incentives for suppliers to counterbalance the high transportation costs and low profit margins in remote areas, (ii) an efficient drug bidding and distribution system, (iii) improved infrastructure and training for physicians to ensure the provision of essential medicines.

Second, medicine prices in remote and underdeveloped areas have been effectively controlled and maintained at lower levels over the long term, owing to both the zero-markup policy in retail price and centralized tendering and procurement. The significant price cut in non-essential medicines also enlighten future policies, suggesting that provincial and periodical centralized procurement (e.g., Volume-Based medicine Purchasing Policy, nationwide collective pharmaceutical procurement) can be potent tools for price regulation (33). Moreover, given the price fluctuation, routine monitoring systems for supply, price and usage are crucial for ongoing improvements, especially in regions with limited development and resources (31, 36).

Third, precise regulations targeted at specific categories of medicines may enhance NEMP’s effectiveness in rural and less developed counties. For instance, the availability of genitourinary and sex hormones and antiparasitic products was low in PHFs under NEMP, while prices of dermatological, genitourinary system and sex hormones, Musculo-skeletal, and sensory organ medicines kept rising. Optimal bidding procurement policies, pricing mechanisms, shortage warning systems, and administrative support are essential to meet patients’ needs for these medicines (32, 34). An affordable, efficient and accessible public-financed reimbursement system is also vital to address the emerging number of innovative and high-price medicines (e.g., oncology treatments) (52). Furthermore, the suboptimal price reduction of TCMs compared to western medicines, accompanied by their growing usage, suggests further regulations on TCMs can be potential directions for policy refinement.

Fourth, the fact that lower prices did not ensure higher consumption indicates that collaborative efforts are necessary to overcome barriers to the use of essential medicines in primary care in remote and rural areas. Safety and quality of essential medicines is a fundamental prerequisite. Regular and regional adjustments to essential medicine lists and centralized procurement and distribution system are needed to align with the needs of local pharmaceutical suppliers, healthcare providers and patients (34). Financial incentives (e.g., enhanced reimbursement rate) and education for practitioners and patients are also effective policy instruments to increase the use of essential drugs (31, 34). A robust and thriving market is key to fostering a virtuous cycle of population health and pharmaceutical development.

Finally, ongoing monitoring and evaluation of NEMP are imperative for future research. Considering the self-adaptive system and responses under top-down policies, it is important to focus on the disproportionate impacts across different regions (undeveloped, rural, and remote areas), populations (older adults), system levels (village, county, city, or province), medicine types, and sectors (public or private). Investigating the mechanisms underlying these expected or unintended outcomes is valuable from the perspectives of different stakeholders (i.e., regulators, public administrators, manufacturers, healthcare providers, patients and third-party payers). Additionally, exploring whether changes in medicine numbers, prices, and usage correlate with improvements in population health remains an essential yet unexplored area of study.

## Conclusion

The NEMP represents a nationwide, systemic, and comprehensive medico-social reform in China. This study investigates the impacts of NEMP on the availability, price, and sales of medicines in a remote, rural and deprived county in Southwestern China. The policy has effectively capped drug prices in primary care in the short-and long-terms, while also exerting spill-over effects in curbing medication costs in secondary care institutions. However, over time, a widening gap in medicine availability and utilization has emerged between rural primary healthcare facilities and county hospitals. While the number of medicines has shown modest increases in county hospitals, it has been on a steady decline in THCs. Similarly, medicine consumption and sales have experienced moderate increases in county hospitals but have risen only marginally in THCs. Ongoing surveillance is necessary, particularly as a reversal of rising price was detected after the second-stage NEMP. Further research and policies are encouraged to address the uneven impacts of NEMP across various pharmaceuticals, care levels, regions and demographics.

## Data Availability

The original contributions presented in the study are included in the article/Supplementary material, further inquiries can be directed to the corresponding author.
