# Supplementary material for: Short- and long-term impacts of the National Essential Medicines Policy on drug availability, price, and usage in a deprived rural county in southwestern China: an interrupted time series analysis across 8 years
Source: Front Public Health. 2024 Aug 29;12:1355239. doi: 10.3389/fpubh.2024.1355239 (PMC11390679; doi:10.3389/fpubh.2024.1355239)
Supplement: Supplementary file 1 [file Data_Sheet_1.pdf]

Appendices

Contents

Appendix 1. Implementation of National Essential Medicines Policy (NEMP) in the selected county2

Appendix 2. Full lists of medicines by policy properties (western, traditional Chinese, essential, non-essential medicines) 4

Appendix 3. Full lists of drugs by Anatomical Therapeutic Chemical Classification System (ATC) and Traditional Chinese Medicine (TCM) classification....14

Appendix 4. Descriptive analysis on medicine types, sales and prices 24

Appendix 5. Number of medicines by western or Traditional Chinese medicines 28

Appendix 6. Number of medicines by ATC system for western medicines and TCM classification .30

Appendix 7. Interrupted time-series analysis on medicine number....32

Appendix 8. Sales of medicines by western or Traditional Chinese medicines 35

Appendix 9. Sales of medicines by ATC system for western medicines and TCM classification (US$1,000)....37

Appendix 10. Interrupted time-series analysis on medicine sales after log transformation 41

Appendix 11. Retail prices of medicines by western or Traditional Chinese medicines 44

Appendix 12. Retail prices of medicines by ATC system for western medicines and TCM classification (Fisher Price Index) 46

Appendix 13. Interrupted time-series analysis on medicine retail prices 48

Appendix 14. Wholesale prices of medicines by western or Traditional Chinese medicines 53

Appendix 15. Wholesale prices of medicines by ATC system for western medicines and TCM classification (Fisher Price Index) 55

Appendix 16. Interrupted time-series analysis on medicine wholesale prices 57

Appendix 17. Sensitivity analysis on drug price index after adjusting for inflation 62

Appendix 1. Implementation of National Essential Medicines Policy (NEMP) in the selected county

Policy[a]	Pre-NEMP (2009.1-2010.11)	Stage 1 NEMP (2010.11 - 2015.10)		Stage 2 NEMP (after 2015.11)
	Health care facilities[b]	Primary care facilities[b]	Secondary care facilities[b]	Secondary care facilities[b]
Selection of medicines	• No limit on selection of medicines	• Compilation and administration of **National Essential Medicines List** (nation-level) and provincial supplementary list (province-level), based on clinical necessity, safety, efficacy, affordability and ease of use. (2010.6)<br>• Revision of lists in every 3 years	/	/
Production, procurement and distribution	• Facilities can freely contact with distributors and procure medicines in need (primary-care level)	• **Centralized tendering and selection** of providers and distributors of essential medicines, based on price-volume-linked and bulk purchasing (province-level; 2010.11)<br>• Establishment of centralized, province-wide, online platform for essential medicine procurement and distribution, at uniform prices (province-level; 2010.11)	• Establishment of centralized, province-wide, online platform for procurement of medicine (including essential medicine) for secondary and tertiary facilities	• Facilities are required to procure medicines on provincial centralized platform as priority; reduction of distributors from 8 to 2 (county-level)
Pricing	• A 15% profit margin was allowed on retail price of medicines, based on the wholesale price (purchase cost from manufacturers and distributors)	• **Zero-markup policy**: the retail price of essential drugs should be equal to the wholesale/ purchase price (primary-care-level; 2010.11)	/	• Zero-markup policy for all drugs (except for Chinese herbal medicine) (county-level)
Clinical use	• Not limit on clinical use of medicines	• Facilities and physicians should **only** stock and use essential medicines on list, based on the	• Use of essential medicines should exceed 30% of	• Use of essential medicines should exceed 50% of total drug use

		clinical guidance on national essential medicines and formulary (primary-care-level; 2010.11)<br>• Facilities are allowed to non-essential drugs after approval, but not exceeding 10% of total drug use (primary-care-level; 2011.5)<br>• Facilities are allowed to off-list drugs after approval, but not exceeding 20% of total drug use (primary-care-level; 2015.1)	total drug use (2012.1)	
Financial compensation for revenue loss due to zero-mark-up policy	/	• Disconnection between service income and operational expenditure of facilities<br>• Financial aids for facilities and physicians, based on service population, area, and performance assessment (State- and county-level; 2012.1)	/	• Price adjustment for medical service and financial aids for facilities (county-level)

a. Policies under the National Essential Medicines Scheme were retrieved from official documents at national, provincial, state, and county levels.

b. Primary care facilities in the county referred to all government-owned township health centres and village clinics; secondary care facilities referred to all government-owned county hospitals.

Appendix 2. Full lists of medicines by policy properties (western, traditional Chinese, essential, non-essential medicines)

Type	Essential drug list	Generic name for western medicine (Pinyin for Traditional Chinese Medicine)
Western medicine	Essential medicine	**A**carbose; Acetagastrodin; Acetylsalicylic acid; Aciclovir; Adenosine; Aescinate; Albendazole; Alfacalcidol; Allopurinol; Alprazolam; Ambroxol; Amikacin; Aminomethylbenzoic acid; Aminophenazone, combinations excl. psycholeptics; Aminophylline; Amiodarone; Amlodipine; Amoxicillin; Amoxicillin and Clavulanate Potassium; Ampicillin; Ampicillin, combinations; Anisodamine; Aripiprazole; Ascorbic acid (vit C); Atropine; Azathioprine; Azithromycin; **B**acillus licheniformis; Batilol; Beclometasone; Belladonna total alkaloids; Benazepril; Benzathine benzylpenicillin; Benzylpenicillin; Berberine; BERGENINI COMPOSITAE TABLETS; Betahistine; Bismuth Potassium Citrate; Bismuth, Magnesium and Sodium Bicarbonate; Bisoprolol; Budesonide; Bupivacaine; Butenafine; **C**alcium carbonate; Calcium Carbonate and Vitamin D3; Calcium chloride; Calcium folinate; Calcium gluconate; Captopril; Carbamazepine; Carbamide; Cefaclor; Cefalexin; Cefathiamidine; Cefazolin; Cefixime; Cefmetazole; Cefoperazone, combinations; Cefotaxime; Cefpiramide; Cefradine; Ceftazidime; Ceftriaxone; Cefuroxime; Cerebroprotein; Chloramphenicol; Chlorphenamine; Chlorpromazine; Chlortetracycline; Chondroitine; Chymotrypsin; Ciclosporin; Ciprofloxacin; Citicoline; Clarithromycin; Clindamycin; Clopidogrel; Clotrimazole; Clozapine; Codeine; Codeine, combinations excl. psycholeptics; Coenzyme A; Colchicine; Colloidal Bismuth Pectin; Combinations of barbiturates; Commpound Vitamin B; Compound Aluminium Hydroxide; Compound Amino Acid 18AA; Compound Liquorice; Compound Sodium Chloride; Cortisone; Cyanocobalamin; Cyclophosphamide; Cyproheptadine; Cytochrome C; **D**eslanoside; Desmopressin; Desogestrel and estrogen; Desonide; Dexamethasone; Dexamethasone, combinations; Dexibuprofen; Dextran; Diammonium Glycyrrhizinate; Diazepam; Diclofenac; Diethylstilbestrol; Difenidol; Digoxin; Diphenhydramine; Dipyridamole; Dobutamine; Domperidone; Dopamine; Doxepin; Doxycycline; **E**conazole; Edaravone; Enalapril; Ephedrine; Epinephrine; Ergocalciferol; Erythromycin; Erythromycin, combinations; Estazolam; Etamsylate; Ethacridine lactate; Ethambutol; **F**amotidine; Fat emulsions; Felodipine; Fenbufen; Fenofibrate; Fentanyl; Ferrous succinate; Fluconazole; Flunarizine; Fluocinolone acetonide; Fluorouracil; Folic acid; Fosfomycin; Furazolidone; Furosemide; **G**entamicin; Glibenclamide; Gliclazide; Glipizide; Glucurolactone; Glutathione; Glycerine Enema or Sorbitol Enema; Glyceryl trinitrate; **H**aemocoagulase Atrox; Haloperidol; Heparin; Hepatocyte Growth-promoting Factors; Huperzine A; Hydrobenzole; Hydrochlorothiazide; Hydrocortisone; Hydroxyethylstarch; **I**buprofen; Ichtasol; Indapamide; Indometacin; Inosine; Insulin (human); Insulin aspart; Insulin detemir; Insulin glargine; Iohexol; Irbesartan; Isoflurane; Isoniazid; Isoprenaline; Isosorbide dinitrate; Isosorbide mononitrate; **K**etamine; **L**actasin; Levodopa and Benserazide Hydrochloride; Levofloxacin; Levothyroxine sodium; Lidocaine; Lincomycin; Lithium; Live Combined Bacillus Subtilis and Enterococcus Faecium; Live Combined Bifidobacterrium,Lactobacillus and Enterococcus; Lobeline; Loratadine; Lovastatin; **M**agnesium sulfate; Mannitol; Medroxyprogesterone;

		Meglumin diatrizoate; Menadione; Metamizole sodium; Metaraminol; Metformin; Methotrexate; Methylprednisolone; Methylthioninium chloride; Metoclopramide; Metoprolol; Metronidazole; Mexiletine; Miconazole; Midazolam; Mifepristone; Misoprostol; Mitomycin; Morphine; Mosapride; Multivitamins and other minerals, incl. combinations; **Naloxone**; Naphazoline, combinations; Neostigmine; Nicergoline; Nifedipine; Nikethamide; Nimodipine; Nitrendipine; Nitrofurantoin; Nitroprusside; Nitroprusside sodium; Norepinephrine; Norfloxacin; Nystatin; Nystatin, combinations; **O**floxacin; Omeprazole; Ondansetron; Ordinary salt combinations; Oryzanol; Oxaliplatin; Oxytocin; **P**aracetamol, combinations excl. psycholeptics; Paroxetine; Penehyclidine; Penfluridol; Pentoxyverine; Perphenazine; Pethidine; Phenobarbital; Phenolphthalein; Phenoxymethylpenicillin; Phentolamine; Phenytoin; Phytomenadione; Pilocarpine; Piracetam; Polyene Phosphatidylcholine ; Potassium chloride; Potassium Dehydroandrograpolide Succinate; Pralidoxime; Prednisone; Procaine; Procaine, combinations; Progesterone; Promethazine; Propafenone; Propofol; Propranolol; Propylthiouracil; Protamine; Pseudoephedrine, combinations; Pyrazinamide; Pyridoxine (vit B6); **R**anitidine; Remifentanil; Repaglinide; Reserpine; Reserpine and diuretics, combinations with other drugs; Reserpine, combinations; Ribavirin; Riboflavin (vit B2); Rice Bran Sterol; Rifampicin; Risperidone; Roxithromycin; Salbutamol; Simvastatin; Smectite; **S**odium bicarbonate; Sodium Lactate Ringer's; Spironolactone; Sterile Water for Injection; Steroidal saponins; Streptomycin; Sucralfate; Sulfamethoxazole; Sulfasalazine; Sulfur; Sulpiride; Suxamethonium; **T**amsulosin; Terazosin; Terbutaline; Testosterone; Tetanus antitoxin; Tetracaine; Tetracycline; Thiamazole; Thiamine (vit B1); Thrombin; Timolol; Tinidazole; Tobramycin; Tocopherol (vit E); Tranexamic acid; Triamcinolone; Trihexyphenidyl; Tripterysium Glycosides; Tropicamide; **U**rokinase; Ursodeoxycholic acid; **V**alproic acid; Valsartan; Vecuronium; Verapamil; Vitamin U,Belladonna and Aluminium Capsules; **W**arfarin
	Non-essential medicine	**A**bacavir; Aceclofenac; Acetylcysteine; Adefovir dipivoxil; Alanyl glutamine; Albumin tannate; Alendronic acid; Almagate; Ambroxol Hydrochloride and Clenbuterol Hydrochloride; Amino acids; Amiotide; Amoxicillim and Sulbactam; Atenolol; Atorvastatin; Atracurium; Azlocillin; Aztreonam; **B**CG vaccine; Benorilate; Benzbromarone; Benzoyl peroxide; Betamethasone; Bismuth preparations, combinations; Bozhi Glycopeptide; Bucinnazine; Bulleyaconitine A; Buspirone; Butylscopolamine and analgesics; **C**alcitriol; Calcium acetate anhydrous; Calcium Chloride and Sodium Bromide; Calcium dobesilate; Calcium lactate; Calcium phosphate; Candesartan; Capecitabine; Carbachol; Carbazochrome; Carbazochrome; Carbocisteine; Carboprost; Carvedilol; Cattle Encephalon Glycoside and Ignotin; Cefadroxil; Cefaloridine; Cefepime; Ceftizoxime; Celecoxib; Cervus and Cucumis Polypeptide; Cervus Nippon Temmick; Cetirizine; Chitosan; Chlorhexidine; Chlorphenamine, combinations; Cimetidine; Cisplatin; Clomifene; Coated Aldehyde Oxystarch; Combinations of electrolytes; Compound Ammonium Glycyrrhetate; Compound Bismuth Aluminate; Compound Carraghenate; Compound Diisopropylamine Dichloroacetate; Compound Ketoconazole; CompoundChlorhexidineHydrochlorideDusting; Conjugated estrogens; Cromoglicic acid;

		Cyclic adenosine monophosphate; Cydiodine Buccal; Cyproterone and estrogen; **D**aidzein; Deoxynucleotide; Desloratadine; Desoxyribonuclease; Dexketoprofen tromethamine; Dexmedetomidine; Dextriferron; Dextromethorphan; Dezocine; Diacerein; Diltiazem; Dimefline; Disodium Cantharidinate; Doxofylline; Doxorubicin; Droperidol; Dyclonine; **E**bastine; Efavirenz; Elcatonin; Enflurane; Entecavir; Enteral Nutritional Suspension; epalrestat; Ephedrine, combinations; Epirubicin; Erythropoietin; Escitalopram; Esmolol; Estradiol; Ethacridine lactate; Ethylenediamine Diaceturate; Etimicin; Etomidate; Etoposide; Etoricoxib; Extract of Horse Chestnut Seeds; **F**at Emulsion, Amino Acids and Glucose; Febuxostat; Ferrous fumarate; Ferrous gluconate; Ferrous Lactate; Ferulate; Fibrinolysin; Finasteride; Flavonihippopha; Fleroxacin; Fluoxetine; Fluvastatin; Formoterol; Foscarnet; Fructose; Fructose 1,6-diphosphate; Fusidic acid; **G**abapentin; Gabexate Mesilate; Gadopentetate dimeglumine; Galantamine; Ganciclovir; Gastrodin; Gastromucin; Gatifloxacin; Gemcitabine; Gemfibrozil; Ginkgo Leaf Extract and Dipyridamole; Glucosamine; Glucose, combinations; Glutaraldehyde; Glycerol; Granisetron; Griseofulvin; **H**alcinonide; Human Epidermal Growth Factor; Hydrogen peroxide; Hydroxychloroquine; Ibuprofen, combinations; Immunoglobulins, normal human, for intravascular adm.; Interleukin; Invert sugar; iodizedlecithin; Ioversol; Ipratropium bromide; Iron, multivitamins and minerals; Iron, vitamin B12 and folic acid; Itraconazole; **K**itasamycin; **L**abetalol; Lacidipine; Lactic acid producing organisms; Lactulose; Lamivudine; Lansoprazole; Leflunomide; Leucogen; Levamisole; Levamlodipine; Levocarnitine; Levornidazole; Levosimendan; L-Glutamine and Sodium Gualenate; Lidocaine, combinations; Ligustrazine; Lindane; Lomefloxacin; Lopinavir/Ritonavir; Lornoxicam; Losartan; Loxoprofen; Lycopene; Lysine,Inosite and Vitamin B12; **M**annatide; Mebendazole; Meclofenoxate; Mecobalamin; Medicinal charcoal; Medroxyprogesterone; Melitracen and psycholeptics; Meloxicam; Meropenem; Methenamine; Metronidazole,Clotrimazole and Chlorhexidine Acetate Suppositories; Mezlocillin; Micronomicin; Microspheres of human albumin; Midecamycin; Miglitol; Milrinone; Mirtazapine; Montelukast; Moroxydine; Mouse Nerve Growth Factor; Moxifloxacin; Mucopolysaccharide Polysulfate; Multienzymes (lipase, protease etc.); Mupirocin; Muscular Amino Acids and Peptides and Nucleosides; Mycophenolate Mofetil; **N**abumetone; Naltrexone; Naphazoline; Naproxen; Nattokinase; Neomycin; Netilmicin; Nevirapine; Nifuratel; **O**ctreotide; Olanzapine; Olmesartan medoxomil; Ornidazole; Oxacillin; Oxiracetam; Oxybuprocaine; Oxytetracycline; Ozagrel; **P**aclitaxel; Pantoprazole; Parecoxib; Pazufloxacin; Pefloxacin; Peginterferon alfa-2b; Perindopril; Phenylbutazone; Phloroglucinol; Pholcodine; Pidotimod; Pioglitazone; Piperacillin; Pirenoxine; Policresulen; Poly I:C; Polymyxin B; Polystyrene sulfonate; Poractant Alfa; Posterior Pituitary Injection; Potassium Magnesium Aspartate; Potassium permanganate; Pregabalin; protein-free calf blood extract; **Q**uetiapine; Quinine, combinations with psycholeptics; **R**abeprazole; Racecadotril; Recombinant Bovine Basic Fibroblast Grow; Recombinant Human Granulocyte Colony-Stimulating Factor Injection; Recombinant Human Granulocyte Colony-Stimulating Factor Injection; Recombinant Human Granulocyte Colony-Stimulating Factor Injection; Recombinant human

		growthhormone; Rifampicin, pyrazinamide, ethambutol and isoniazid; Rifamycin; Rivaroxaban; Rocuronium bromide; Ropivacaine; Rosuvastatin; **S**accharated iron oxide; Salcatonin; SALMETEROL Fluticasone; Sarpogrelate; Scopolamine; Sertaconazole; Sertraline; Sevoflurane; Silibinin; Silybin Meglumine; Sirolimus; Sodium citrate; Sodium Glucuronic Acid; Sodium Hyaluronate; Sodium hypochlorite; Somatostatin; Spectinomycin; Spiramycin; Stavudine; Streptokinase; Strophanthidin; Succinylated Gelatin; Sufentanil; Sulfamethoxazole and trimethoprim; Sulfotanshinone Sodium; **T**acrolimus; Tamoxifen; Technetium (99mTc) medronic acid; Tegafur; Tegafur, combinations; Telmisartan; Temozolomide; Tenofovirdisoproxil; Terfenadine; Tetanus immunoglobulin; Thalidomide; Thiamphenicol; Thymalfasin; Thymopetidum; Thyroid gland preparations; Tiopronin; Tiotropium bromide; Total glucosides of paeonia; Tramadol; Transfer Factor; Trimetazidine; Tropicamide, combinations; Tropisetron; Troxerutin; Trypsin, combinations; **U**benimex; Urapidil; Valsartan and amlodipine; **V**ancomycin; Venlafaxine; Vidarabine; Vinpocetine; Vitamin B4; **Z**idovudine; Zinc gluconate
Chinese Traditiona l Medicine	Essential medicine	(Pinyin) **An** Gong Niu Huang Wan; An Shen Bu Nao Ye; An Shen Bu Xin Wan; **Ba** Zhen Yi Mu Wan (Jiao Nang); Bai Ji Ke Li (Fen, Tang Jiang); Bai Zi Yang Xin Wan; Ban Lan Gen Ke Li; Ban Lan Gen Pian; Ban Lan Qing Re Ke Li; Bao Ji Wan (Kou Fu Ye); Bi Yan Kang Pian; Bing Peng San; Bu Zhang Nao Xin Tong Jiao Nang; Bu Zhong Yi Qi Wan (Ke Li); **Can** Fu Zhu She Ye; Can Ling Bai Shu San (Wan, Ke Li); Can Ling Jian Pi Wei Ke Li; Can Mai Zhu She Ye; Can Qi Jiang Tang Pian /Jiao Nang; Can Song Yang Xin Jiao Nang; Chai Hu Zhu She Ye; Chen Xiang Lu Bai Lu Pian; Ci Wu Jia Nao Ling Ye; **Da** Huo Luo Wan (Jiao Nang); Dan Can Zhu She Ye (Dong Gan /Fen Zhen); Dan Deng Tong Nao Jiao Nang (Ruan Jiao Nang); Dan Hong Zhu She Ye; Deng Zhan Hua Su Pian; Deng Zhan Hua Su Zhu She Ye; **Er** Long Zuo Ci Wan; **Feng** Han Gan Mao Ke Li; Feng Re Gan Mao Ke Li; Fu Fang Dan Can Pian (Ke Li, Jiao Nang, Di Wan); Fu Fang Feng Shi Ning Jiao Nang (Pian); Fu Fang Nan Ban Lan Gen Pian; Fu Fang Nan Xing Zhi Tong Gao; Fu Fang Sha Ji Zi You Shuan; Fu Fang Xue Shuan Tong Jiao Nang (Pian); Fu Ke Qian Jin Pian (Jiao Nang); Fu Ling Bai Shu Ke Li; Fu Yan Kang Pian; **Gan** Mao Ling (Ke Li, Chong Ji, Jiao Nang, Pian); Gan Mao Qing Re Ke Li (Jiao Nang); Gan Mao Shu Feng Pian (Ke Li, Wan); Gan Mao Xiao Yan Pian; Geng Nian An Pian (Jiao Nang); Gong Liu Xiao Jiao Nang; Gong Xue Ning Jiao Nang; Gou Pi Gao; Gu Shang Yu He Ji; Guan Xin Ning Zhu She Ye; Gui Pi Wan (He Ji); Gui Zhi Fu Ling Wan (Jiao Nang); **Han** Chuan Zu Pa Ke Li; Heng Gu Gu Shang Yu He Ji; Hong Hua Huang Se Su; Hu Gan Pian (Ke Li, Jiao Nang); Hu Li San Jiao Nang; Hua Hong Pian (Ke Li, Jiao Nang); Hua Tan Li Yan Huang Shi Xiang Sheng Wan; Hua Tuo Zai Zao Wan; Huang Lian Shang Qing Wan (Ke Li, Jiao Nang, Pian); Huang Qi Ke Li; Huang Qi Zhu She Ye; Huang Teng Su Pian (Fen San Pian, Jiao Nang, Ruan Jiao Nang); Huo Xiang Zheng Qi Jiao Nang; Huo Xiang Zheng Qi Shui (Kou Fu Ye, Ruan Jiao Nang, Ke Li); Huo Xue Zhi Tong San (Jiao Nang); **Ji** De Sheng She Yao Pian; Ji Zhi Tang Jiang (Ke Li); Jian Wei Xiao Shi Pian; Jie Er Yin Xi Ye; Jie Gu Qi Li Pian; Jin Dan Pian; Jin Gang Teng Jiao Nang; Jin Kui Shen Qi Wan (Pian); Jin Qian Cao Ke Li; Jing Fu Kang Ke Li; Jing Shu Ke Li; Jing Wan Hong Ruan Gao; Ju Hong Wan (Ke Li, Jiao Nang, Pian); **Kang** Bing Du He Ji;

		Kang Bing Du Kou Fu Ye (Ke Li); Kang Fu Xin Ye; Kang Gan Ling Pian; Kang Gong Yan Pian /Jiao Nang; Ke Ke Tang Jiang; Kui Hua Hu Gan Pian; **Li** Dan Zhi Tong Jiao Nang /Pian; Lian Hua Qing Wen Jiao Nang; Lian Hua Qing Wen Jiao Nang (Ke Li); Liu Wei Di Huang Wan (Ke Li, Jiao Nang); Long Xue Jie Jiao Nang; Ma Ren Run Chang Wan (Ruan Jiao Nang); **Ma** Ying Long Zhi Chuang Gao; Ming Mu Di Huang Wan; Mo Guan Fu Kang Jiao Nang; Mo Luo Ning Zhu She Ye; Mo Xue Kang Jiao Nang; **Nao** Xin Shu Kou Fu Ye; Nao Xin Tong Wan (Jiao Nang, Pian); Niu Huang Jie Du Wan (Jiao Nang, Ruan Jiao Nang, Pian); Niu Huang Shang Qing Wan (Jiao Nang, Pian); **Pai** Shi Ke Li; Pen Yan Jing Ke Li (Jiao Nang); Pu Le An Jiao Nang (Pian); **Qi** Ju Di Huang Wan (Jiao Nang, Pian); Qi Ye Shen An Pian; Qi Zhi Wei Tong Ke Li (Pian); Qiang Li Pi Pa Lu; Qing Fei Hua Tan Wan; Qing Fei Yi Huo Pian; Qing Hou Li Yan Ke Li; Qing Huo Zhi Mai Pian; Qing Kai Ling Ke Li (Jiao Nang, Pian, Zhu She Ye); Qing Xuan Zhi Ke Ke Li; **Ren** Can Zai Zao Wan; Ru Pi Xiao Ke Li (Jiao Nang, Pian); Run Zao Zhi Yang Jiao Nang; **San** Huang Pian; San Jin Pian; San Jiu Wei Tai Ke Li (Jiao Nang); San Qi Pian; Se Chang Zhi Xie San; Shang Ke Jie Gu Pian; She Dan Chen Pi Kou Fu Ye; She Dan Chuan Bei Ye; Sheng Mo Yin (Ke Li, Jiao Nang, Zhu She Ye); Shi Di Shui; Shu Gan Ke Li; Shu Jin Huo Xue Wan (Pian, Jiao Nang); Shu Mi Tong Jiao Nang; Shu Xin Tong Mo Jiao Nang; Shuang Huang Lian He Ji (Kou Fu Ye, Ke Li, Jiao Nang, Pian); Shuang Huang Lian Zhu She Ye; Si Mo Tang Kou Fu Ye; Si Shen Wan (Pian); Su Xiao Jiu Xin Wan; **Tian** Ma Xing Nao Jiao Nang; Tian Qi Hua Ye Ke Li; Tian Wang Bu Xin Wan (Pian); Tong Bian Ling Jiao Nang; Tong Luo Qu Tong Gao; Tong Qiao Bi Yan Pian; Tong Shu Jiao Nang; Tong Xin Luo Jiao Nang; Tong Xuan Li Fei Wan (Ke Li, Jiao Nang, Pian); **Wang** Bi Ke Li (Jiao Nang, Pian); Wei Su Ke Li; Wen Xin Ke Li; Wu Di Dan Jiao Nang; Wu Ji Bai Feng Wan (Jiao Nang, Pian); **Xian** Ling Gu Bao Jiao Nang (Pian); Xiang Guo Jian Xiao Pian; Xiang Ju Jiao Nang (Pian); Xiang Lian Pian; Xiang Sha Ping Wei Wan (Ke Li); Xiao Chai Hu Ke Li; Xiao Er Bao Tai Kang Ke Li; Xiao Er Chang Wei Kang Ke Li; Xiao Er Hua Du San (Jiao Nang); Xiao Er Hua Tan Zhi Ke Ke Li; Xiao Er Ke Chuan Ling Ke Li; Xiao Er Re Su Qing Kou Fu Ye (Ke Li); Xiao Er Xiao Ji Zhi Ke Kou Fu Ye; Xiao Er Yan Bian Ke Li; Xiao Ke Wan; Xiao Yan Li Dan Pian (Ke Li, Jiao Nang); Xiao Yao Wan (Ke Li); Xiao Zhong Zhi Tong Ding; Xin Fu Fang Da Qing Ye Pian; Xin Ke Shu Jiao Nang (Pian); Xin Qin Ke Li; Xing Pi Yang Er Ke Li; Xiong Dan Yan Yao Shui; Xuan Mai Gan Ju Ke Li; Xue Sai Tong Jiao Nang (Pian); Xue Sai Tong Zhu She Ye (Dong Gan); Xue Zhi Kang Pian (Jiao Nang); **Yang** Xue Qing Nao Wan (Ke Li); Yao Bi Tong Jiao Nang; Yi Mo Kang Pian; Yi Mu Cao Gao (Ke Li, Jiao Nang, Pian); Yi Xin Shu Ke Li (Jiao Nang, Pian); Yin Dan Xin Nao Tong Ruan Jiao Nang; Yin Huang Kou Fu Ye (Ke Li, Jiao Nang, Pian); Yin Qiao Jie Du Wan (Ke Li, Jiao Nang, Ruan Jiao Nang, Pian); Yin Xing Ye Jiao Nang (Pian, Di Wan); Yin Zhi Huang Kou Fu Ye (Ke Li); Yong Long Zheng Hong Hua You; Yu Ping Feng Ke Li; Yuan Hu Zhi Tong Pian (Ke Li, Jiao Nang, Di Wan); Yun Nan Bai Yao (Jiao Nang, Ke Li, Gao, Ding, Qi Wu Ji); Yun Nan Hong Yao Jiao Nang; Yun Tong Ding Jiao Nang; **Zao** Ren An Shen Ke Li (Jiao Nang); Zhen Zhu Ming Mu Di Yan Ye; Zheng Tian Wan (Jiao Nang); Zhi Bai Di Huang Wan; Zhui Feng Tou Gu Wan; Zu Shi Ma Guan Jie Zhi Tong; Zu Shi Ma Zhu She Ye

		Chinese name: 安宫牛黄丸, 安神补脑液, 安神补心丸, 八珍益母丸(胶囊), 白芨颗粒(粉、糖浆), 柏子养心丸, 板蓝根颗粒, 板蓝根片, 板蓝清热颗粒, 保济丸(口服液), 鼻炎康片, 冰硼散, 步长脑心通胶囊, 补中益气丸(颗粒), 参附注射液, 参苓白术散(丸、颗粒), 参苓健脾胃颗粒, 参麦注射液, 参芪降糖片/胶囊, 参松养心胶囊, 柴胡注射液, 陈香露白露片, 刺五加脑灵液, 大活络丸(胶囊), 丹参注射液(冻干/粉针), 丹灯通脑胶囊(软胶囊), 丹红注射液, 灯盏花素片, 灯盏花素注射液, 耳聋左慈丸, 风寒感冒颗粒, 风热感冒颗粒, 复方丹参片(颗粒、胶囊、滴丸), 复方风湿宁胶囊(片), 复方南板蓝根片, 复方南星止痛膏, 复方沙棘籽油栓, 复方血栓通胶囊(片), 妇科千金片(胶囊), 茯苓白术颗粒, 妇炎康片, 感冒灵(颗粒、冲剂、胶囊、片), 感冒清热颗粒(胶囊), 感冒疏风片(颗粒、丸), 感冒消炎片, 更年安片(胶囊), 宫瘤消胶囊, 宫血宁胶囊, 狗皮膏, 骨伤愈合剂, 冠心宁注射液, 归脾丸(合剂), 桂枝茯苓丸(胶囊), 寒喘祖帕颗粒, 恒古骨伤愈合剂, 红花黄色素, 护肝片(颗粒、胶囊), 虎力散胶囊, 花红片(颗粒、胶囊), 化痰利咽黄氏响声丸, 华佗再造丸, 黄连上清丸(颗粒、胶囊、片), 黄芪颗粒, 黄芪注射液, 黄藤素片(分散片、胶囊、软胶囊), 霍香正气胶囊, 藿香正气水(口服液、软胶囊，颗粒), 活血止痛散(胶囊), 季德胜蛇药片, 急支糖浆(颗粒), 健胃消食片, 洁尔阴洗液, 接骨七厘片, 金胆片, 金刚藤胶囊, 金匮肾气丸(片), 金钱草颗粒, 颈复康颗粒, 颈舒颗粒, 京万红软膏, 橘红丸(颗粒、胶囊、片), 抗病毒合剂, 抗病毒口服液(颗粒), 康复新液, 抗感灵片, 抗宫炎片/胶囊, 克咳糖浆, 葵花护肝片, 利胆止痛胶囊/片, 莲花清瘟胶囊, 连花清瘟胶囊(颗粒), 六味地黄丸(颗粒、胶囊), 龙血竭胶囊, 麻仁润肠丸(软胶囊), 马应龙痔疮膏, 明目地黄丸, 脉管复康胶囊, 脉络宁注射液, 脉血康胶囊, 脑心舒口服液, 脑心通丸(胶囊、片), 牛黄解毒丸(胶囊、软胶囊、片), 牛黄上清丸(胶囊、片), 排石颗粒, 盆炎净颗粒(胶囊), 普乐安胶囊(片), 杞菊地黄丸(胶囊、片), 七叶神安片, 气滞胃痛颗粒(片), 强力枇杷露, 清肺化痰丸, 清肺抑火片, 清喉利咽颗粒, 清火栀麦片, 清开灵颗粒(胶囊、片、注射液), 清宣止咳颗粒, 人参再造丸, 乳癖消颗粒(胶囊、片), 润燥止痒胶囊, 三黄片, 三金片, 三九胃泰颗粒(胶囊), 三七片, 涩肠止泻散, 伤科接骨片, 蛇胆陈皮口服液, 蛇胆川贝液, 生脉饮(颗粒、胶囊、注射液), 十滴水, 舒肝颗粒, 舒筋活血丸(片，胶囊), 舒泌通胶囊, 舒心通脉胶囊, 双黄连合剂(口服液、颗粒、胶囊、片), 双黄莲注射液, 四磨汤口服液, 四神丸(片), 速效救心丸, 天麻醒脑胶囊, 田七花叶颗粒, 天王补心丸(片), 通便灵胶囊, 通络祛痛膏, 通窍鼻炎片, 痛舒胶囊, 通心络胶囊, 通宣理肺丸(颗粒、胶囊、片), 尪痹颗粒(胶囊、片), 胃苏颗粒, 稳心颗粒, 无敌丹胶囊, 乌鸡白凤丸(胶囊、片), 仙灵骨葆胶囊(片), 香果健消片, 香菊胶囊(片), 香连片, 香砂平胃丸(颗粒), 小柴胡颗粒, 小儿宝泰康颗粒, 小儿肠胃康颗粒, 小儿化毒散(胶囊), 小儿化痰止咳颗粒, 小儿咳喘灵颗粒, 小儿热速清口服液(颗粒), 小儿消积止咳口服液, 小儿咽扁颗粒, 消渴丸, 消炎利胆片(颗粒、胶囊), 逍遥丸(颗粒), 消肿止痛酊, 新复方大青叶片, 心可舒胶囊(片), 辛芩颗粒, 醒脾养儿颗粒, 熊胆眼药水, 玄麦甘桔颗粒, 血塞通胶囊(片), 血塞通注射液(冻干), 血脂康片(胶囊), 养血清脑丸(颗粒), 腰痹通胶囊, 益脉康片, 益母草膏(颗粒、胶囊、片), 益心舒颗粒(胶囊、片), 银丹心脑通软胶囊, 银黄口服液(颗粒、胶囊、片), 银翘解毒丸(颗粒、胶囊、软胶囊、片), 银杏叶胶囊(片、滴丸), 茵栀黄口服液(颗粒), 永龙正红花油, 玉屏风颗粒, 元胡止痛片(颗粒、胶囊、滴丸), 云南白药(胶囊、颗粒、膏、酊、气雾剂), 云南红药胶囊, 晕痛定胶囊, 枣仁安神颗粒(胶囊), 珍珠明目滴眼液, 正天丸(胶囊), 知柏地黄丸, 追风透骨丸, 祖师麻关节止痛, 祖师麻注射液

	Non-essential medicine	(Pinyin) **Bai** Jie Jiao Nang; Bai Le Mian Jiao Nang; Bai Ling Jiao Nang; Ban Lan Gen Zhen; Ban Xia Tang Jiang; Bao Bao Shi Zhen Gao; Bao Bao Yi Tie Ling; Bao Fei Ning Sou Ke Li; Bao Fu Kang Shuan ; Bao Guang Fu Le Ke Li; Bao Tai Ling; Bi Jie Fen Qing Wan; Bu Shen Jian Gu He Ji; **Can** Qi Wu Wei Zi Tang Jiang; Can Xian Sheng Mo Kou Fu Ye; Can Xiong Pu Tao Tang Zhu She Ye; Chang Qing Cha; Chang Wei Ning Pian; Chang Yan Ning Jiao Nang (Pian); Chu Yi Ming Mu Pian; Chuan Bai Zhi Yang Xi Ji; Chuan Bei Pi Pa Gao; Chuan Bei Pi Pa Tang Jiang; Chuan Ke Zhi Zhu She Ye; Chuan Xin Lian Zhu She Ye; Ci Wu Jia Pian; Ci Wu Jia Zhu She Ye; Cuo Chuang Gao; **Da** Li Tong Ke Li; Dan Bie Jiao Nang; Dan E Fu Kang Jian Gao; Dan Shi Li Tong Pian; Dan Shi Tong Jiao Nang; Dan Shu Jiao Nang; Dang Gui Nian Tong Ke Li; Dao Chi Pian; Deng Tai Ye Ke Li; Deng Zhan Sheng Mo Jiao Nang; Deng Zhan Xi Xin He Ji; Deng Zhan Xi Xin Ke Li; Deng Zhan Xi Xin Zhu She Ye; Di Yu Sheng Bai Pian; Dian Xian Ning Pian; Diao Jing Cu Yun Wan; Die Da Qi Li Pian; Ding Gui Er Qi Tie; Dong Qing Gao; Dou Liang Ruan Jiao Nang; Du Yi Wei Ruan Jiao Nang; **E** Shu You Zhu She Ye; **Fei** Li Ke He Ji Jiao Nang; Feng Liao Chang Wei Kang Ke Li; Feng Shi Ding Jiao Nang; Feng Shi Ning Zhu She Ye; Feng You Jing; Fu Fa Xian He Cao Chang Yan Jiao Nang; Fu Fang A Jiao Jiang; Fu Fang Cao Shan Hu Pian; Fu Fang Chuan Xin Lian Pian; Fu Fang Dang Gui Zhu She Ye; Fu Fang Huang Song Xi Ye; Fu Fang Ku Can Zhu She Ye; Fu Fang Long Xue Jie Jiao Nang; Fu Fang Luo Bu Ma Pian; Fu Fang Yu Xing Cao Tang Jiang; Fu Ke Diao Jing Pian; Fu Ke Zai Zao Wan /Jiao Nang; Fu Ma Di Bi Ye; Fu Ming Pian; Fu Xue Kang Ke Li; Fu Yan Jie; Fu Yan Kang Fu Jiao Nang; Fu Yan Kang Ruan Jiao Nang; **Gan** Ju Bing Mei Pian; Gan Kang Pian; Gan Mao Di Wan; Gan Mao Qing Pian /Jiao Nang; Gan Mao Zhi Ke Tang Jiang; Gan Wei Qi Tong San; Gan Zhi Xiao Jiao Nang; Gang Tai; Gong Lao Qu Huo Jiao Nang; Gu Bi He Ji; Gu Can Chang An Jiao Nang; Gu Ci Ping Pian; Gu Long Jiao Nang; Gu Shu Kang Jiao Nang; Gu Yu Ling Jiao Nang; Gu Zeng Sheng Zhen Tong Gao; Gu Zhi Zeng Sheng Tie; Guan Xin Shu Tong Jiao Nang; Gui Fu Li Zhong Wan; Gui Long Jin Gu Ning Pian; **Ha** Jie Ding Chuan Jiao Nang; Hai Kun Shen Xi Jiao Nang; He Lao; He Xue Ming Mu Pian; Hong He Fu Jie Xi Ye; Hong Hua Zhu She Ye; Hong Hui Xiang Zhu She Ye; Hou Er Huan Xiao Yan Ke Li; Hua Ji Kou Fu Ye; Huang Jin Wan Hong Gao; Huang Qi Sheng Mo Yin; Huang Teng Su Zhu She Ye; **Ji** Yan Gao; Jian Er Qing Jie Ye; Jian Min Yan Hou Pian; Jian Pi Yang Gan Wan; Jian Yang Jiao Nang; Jian Zhou Yan Gao Yao; Jiang Tang Wan; Jie Gu Pian; Jie Gu Xu Jin Jiao Nang; Jie Jiu Ling Kou Fu Ye; Jie Yu An Shen Ke Li; Jin Ji Jiao Nang; Jin Lian Wei Shu Pian; Jin Long Jiao Nang; Jin Ma Gan Tai Ke Li; Jin Qian Tong Lin Ke Li; Jin Sang Kai Yin Wan; Jin Sang Li Yan Wan; Jin Sang Zi Hou Bao; Jin Shui Bao Jiao Nang; Jin Zhen Kou Fu Ye; Jin Zhi Jie Yin Han Shu Ye; Jing Dou Nian Ci Yan Mi Lian Chuan Bei Pi Pa Gao; Jing Long Xiao Tong Tie; Ju Geng Ma Huang Jian Tang Jiang; Juan Bi Ke Li; **Kai** Hou Jian Pen Wu Ji; Kang Ai Zhu She Ye; Kang Fu Ning Jiao; Kang Fu Yan Jiao Nang; Kang Fu Yan Jiao Nang; Kang Gan Ke Li; Kang Gu Zeng Sheng Pian; Ke Ke Jiao Nang; Ke Lu Kou Fu Ye; Ke Su Ting Tang Jiang; Ke Yang Shu Xi Ye; Kou Qiang Yan Pen Wu Ji; Ku Die Zi Zhu She Ye; Kui Hua Wei Kang Ling; Kui Yang Ling Jiao Nang; **Lan** Qin Kou Fu Ye; Li Shu Kang Jiao Nang; Li Xu Wang Xue Sai Tong Ruan Jiao Nang; Ling Zhi Yi Shou Jiao Nang; Liu Jing Tou Tong Pian; Liu Ling Wan; Long

		Hu Pai Qing Liang You; Long Mu Zhuang Gu Ke Li; **Man** Yan Shu Ning Ke Li; Mei Bao Shi Run Shao Shang Gao; Mu Ru Duo Ke Li; Nao Li Qing Wan; **Nao** Ling Su Jiao Nang; Nao Luo Tong Jiao Nang; Niao Du Qing Ke Li; Niao Gan Ning Ke Li; Ning Xin Bao Jiao Nang; Nu Bao Jiao Nang; Nu Jin Pian; Nuan Gong Qi Wei Wan; **Pai** Du Yang Yan Jiao Nang; **Qi** Li Qiang Xin Jiao Nang; Qi Zheng Xiao Tong Tie; Qian Bai Bi Yan Pian; Qian Jin Xi Ye; Qian Lie Ci Sheng Tie; Qian Lie Shu Tong Jiao Nang; Qian Zi Hong Ke Li; Qiang Li Nao Qing Su Pian; Qin Qiao Kou Fu Ye; Qing Hou Yan Ke Li; Qing Liang You; Qing Re Jie Du Kou Fu Ye; Qing Re San Jie Pian; Qing Re Zhi Ke Ke Li; Qing Wei Huang Lian Wan; Qiu Xie Ling He Ji; Qu Feng Zhi Tong Jiao Nang; **Re** Du Ning Zhu She Ye; Re Du Qing Pian; Ren Can Feng Wang Jiang; Rou Kou Wu Wei Wan; Ru Kang Pian; Ru Ning Pian; Run Chang Jiao Nang; **Sang** Ju Gan Mao Pian; Sha Ji Gan Ru Ji; Sha Qi Wan; Shan Zha Ke Li; Shang Ke Die Da Pian; Shao Bei Zhu She Ye; Shao Lin Feng Shi Die Da Gao; She Xiang Bao Xin Wan; She Xiang Zhi Tong Tie Gao; She Xiang Zhuang Gu Gao; She Xiang Zhui Feng Gao; Shen Bao He Ji; Shen Bao Tang Jiang; Shen Shi Tong Ke Li; Shen Shuai Ning Jiao Nang; Shi Du Qing Jiao Nang; Shi Lin Tong Ke Li; Shu Jin Jian Yao Wan; Shu Xin Jiang Zhi Pian; Shu Xue Ning Zhu She Ye; Shu Xue Tong Zhu She Ye; Si Huang Ruan Gao; Si Ji Gan Mao Jiao Nang; Song Jie You; **Tan** Ke Jing San (Pian); Tan Re Qing Zhu She Ye; Tian He Gu Tong Gao; Tian He Zhui Feng Gao; Tian Ma Tou Tong Pian; Tian Ma Wan; Tong Feng Shu Pian; Tong Feng Xiao Ke Li; Tong Jing Bao Ke Li; Tong Shu Jiao Nang; Tong Zhi Su Run Jiang Jiao Nang; Tou Tong Ning Jiao Nang; **Wan** Tong Jin Gu Tie; Wei Cyin Qiao Pian; Wei Fu Chun Pian; Wei Kang Ling Jiao Nang; Wen Pi Gu Chang San; Wen Wei Shu Ke Li; Wu Lin Hua Shi Wan; Wu Zhi Jiao Nang; **Xi** Gua Shuang Pian; Xi Yan Ping Zhu She Ye; Xian Zhu Li Kou Fu Ye; Xiang Dan Zhu She Ye; Xiang Pi Gao; Xiang Sha Wei Tong San; Xiao Er Fei Re Ke Chuan Kou Fu Ye; Xiao Er Qing Yan Ke Li; Xiao He Pian; Xiao Ke Chuan Tang Jiang; Xiao Ru San Jie Jiao Nang; Xiao Shuan Tong Luo Jiao Nang; Xiao Shuan Tong Luo Ke Li; Xiao Xuan Zhi Yun Pian; Xiao Yan Ruan Gao; Xiao Yan Zhi Tong Gao; Xie Gong Jiao Nang; Xin Bao Wan; Xin Nao Qing Ruan Jiao Nang; Xin Sheng Hua Ke Li; Xin Shu Bao Jiao Nang; Xing Nao Jing Zhu She Ye; Xing Xiang Tu Er Feng; Xu Zhang Qing; Xue Bi Jing Zhu She Ye; Xue Zhi Tong Jiao Nang; **Yan** Li Shuang Kou Han Di Wan; Yan Yan Pian; Yang Shen Jiao Nang; Yang Wei Shu Ke Li; Yang Xue An Shen Ke Li; Yang Xue Sheng Fa Jiao Nang; Yao Xi Tong Jiao Nang; Ye Mu Gua Pian; Ye Su Jiao Nang; Yi Gan Fu Zheng Jiao Nang; Yi Gan Jie Du Jiao Nang; Yi Jin Jiang Tang Jiao Nang; Yi Mu Kang Ke Li; Yi Qi Bu Xue Pian; Yi Tong Shu Zhu She Ye; Yi Xue Sheng Jiao Nang; Yi Zhi Hao Shang Shi Qu Tong Gao; Yin Xing Mi Huan Kou Fu Ye; Yin Xing Ye Zhu She Ye; Yin Zhi Huang Zhu She Ye; Ying Er Jian Pi Ke Li; Ying Er Jian Pi San; Yu Biao Bu Shen Wan; Yu Feng Ning Xin Di Wan; Yu Ping Feng Kou Fu Ye; Yu Xing Cao Pian; Yun Kang Kou Fu Ye; Yun Kang Ying Yang Ye; **Zhen** Nao Ning Jiao Nang; Zheng Gu Shui; Zheng Hong Hua You; Zheng Qing Feng Tong Ning Huan Shi Pian; Zheng Qing Feng Tong Ning Zhu She Ye; Zhi Chuan Ling Qi Wu Ji; Zhi Ke Wan; Zhi Tong Hua Zheng Pian (Jiao Nang); Zhi Xie Bao Tong Ke Li; Zhi Xie Li Ke Li; Zhi Xue Jiao Nang; Zhong Tong Cha Ji; Zhong Tong Qi Wu Ji; Zhu Hong Jun Su Ruan Gao; Zhuang Gu She Xiang Zhi Tong Gao; Zi Cao San Huang Ruan Gao; Zi

		Xin Yin Ke Li<br><br>Chinese name: 百解胶囊, 百乐眠胶囊, 百令胶囊, 板蓝根针, 半夏糖浆, 宝宝湿疹膏, 宝宝一贴灵, 保肺宁嗽颗粒, 保妇康栓 , 宝光妇乐颗粒, 保胎灵, 萆解分清丸, 补肾健骨合剂, 参芪五味子糖浆, 参仙升脉口服夜, 参芎葡萄糖注射液, 肠清茶, 肠胃宁片, 肠炎宁胶囊(片), 除翳明目片, 川百止痒洗剂, 川贝枇杷膏, 川贝枇杷糖浆, 喘可治注射液, 穿心莲注射液, 刺五加片, 刺五加注射液, 痤疮膏, 达立通颗粒, 丹鳖胶囊, 丹莪妇康煎膏, 胆石利通片, 胆石通胶囊, 胆舒胶囊, 当归拈痛颗粒, 导赤片, 灯台叶颗粒, 灯盏生脉胶囊, 灯盏细辛合剂, 灯盏细辛颗粒, 灯盏细辛注射液, 地榆升白片, 癫痫宁片, 调经促孕丸, 跌打七厘片, 丁桂儿脐贴, 冬青膏, 都梁软胶囊, 独一味软胶囊, 莪术油注射液, 肺力咳合剂胶囊, 枫蓼肠胃康颗粒, 风湿定胶囊, 风湿宁注射液, 风油精, 复发仙鹤草肠炎胶囊, 复方阿胶浆, 复方草珊瑚片, 复方穿心莲片, 复方当归注射液, 复方黄松洗液, 复方苦参注射液, 复方龙血竭胶囊, 复方罗布麻片, 复方鱼腥草糖浆, 妇科调经片, 妇科再造丸/胶囊, 呋麻滴鼻液, 复明片, 妇血康颗粒, 妇炎洁, 妇炎康复胶囊, 妇炎康软胶囊, 甘桔冰梅片, 肝康片, 感冒滴丸, 感冒清片/胶囊, 感冒止咳糖浆, 肝胃气痛散, 肝脂消胶囊, 肛泰, 功劳去火胶囊, 骨痹合剂, 谷参肠安胶囊, 骨刺平片, 骨龙胶囊, 骨疏康胶囊, 骨愈灵胶囊, 骨增生镇痛膏, 骨质增生贴, 冠心舒通胶囊, 桂附理中丸, 归龙筋骨宁片, 蛤蚧定喘胶囊, 海昆肾喜胶囊, 核酪, 和血明目片, 红核妇洁洗液, 红花注射液, 红茴香注射液, 猴耳环消炎颗粒, 化积口服液, 黄金万红膏, 黄芪生脉饮, 黄藤素注射液, 鸡眼膏, 健儿清解液, 健民咽喉片, 健脾养肝丸, 健阳胶囊, 肩周炎膏药, 降糖丸, 接骨片, 接骨续筋胶囊, 解酒灵口服液, 解郁安神颗粒, 金鸡胶囊, 金莲胃舒片, 金龙胶囊, 金马肝泰颗粒, 金钱通淋颗粒, 金嗓开音丸, 金嗓利咽丸, 金嗓子喉宝, 金水宝胶囊, 金振口服液, 金栀洁龈含漱液, 京都念慈菴蜜炼川贝枇杷膏, 京龙消痛贴, 桔梗麻黄碱糖浆, 蠲痹颗粒, 开喉剑喷雾剂, 康艾注射液, 康妇凝胶, 康妇炎胶囊, 抗妇炎胶囊, 抗感颗粒, 抗骨增生片, 克咳胶囊, 咳露口服液, 咳速停糖浆, 克痒舒洗液, 口腔炎喷雾剂, 苦碟子注射液, 葵花胃康灵, 溃疡灵胶囊, 蓝芩口服液, 利舒康胶囊, 理洫王血塞通软胶囊, 灵芝益寿胶囊, 六经头痛片, 六灵丸, 龙虎牌清凉油, 龙牡壮骨颗粒, 慢严舒柠颗粒, 美宝湿润烧伤膏, 母乳多颗粒, 脑立清丸, 脑灵素胶囊, 脑络通胶囊, 尿毒清颗粒, 尿感宁颗粒, 宁心宝胶囊, 女宝胶囊, 女金片, 暖宫七味丸, 排毒养颜胶囊, 芪苈强心胶囊, 奇正消痛贴, 千柏鼻炎片, 千金洗液, 前列磁生贴, 前列舒通胶囊, 千紫红颗粒, 强力脑清素片, 芩翘口服液, 清喉咽颗粒, 清凉油, 清热解毒口服液, 清热散结片, 清热止咳颗粒, 清胃黄连丸, 秋泻灵合剂, 祛风止痛胶囊, 热毒宁注射液, 热毒清片, 人参蜂王浆, 肉蔻五味丸, 乳康片, 乳宁片, 润肠胶囊, 桑菊感冒片, 沙棘干乳剂, 痧气丸, 山楂颗粒, 伤科跌打片, 芍倍注射液, 少林风湿跌打膏, 麝香保心丸, 麝香止痛贴膏, 麝香壮骨膏, 麝香追风膏, 肾宝合剂, 肾宝糖浆, 肾石通颗粒, 肾衰宁胶囊, 湿毒清胶囊, 石淋通颗粒, 舒筋健腰丸, 舒心降脂片, 舒血宁注射液, 疏血通注射液, 四黄软膏, 四季感冒胶囊, 松节油, 痰咳净散(片), 痰热清注射液, 天和骨通膏, 天和追风膏, 天麻头痛片, 天麻丸, 痛风舒片, 痛风消颗粒, 痛经宝颗粒, 通舒胶囊, 通滞苏润江胶囊, 头痛宁胶囊, 万通筋骨贴, 维 C 银翘片, 胃复春片, 胃康灵胶囊, 温脾固肠散, 温胃舒颗粒, 五淋化石丸, 五酯胶囊, 西瓜霜片, 喜炎平注射液, 鲜竹沥口服液, 香丹注射液, 象皮膏, 香砂胃痛散, 小儿肺热咳喘口服液, 小儿清咽颗粒, 消核片, 消咳喘糖浆, 消乳散结胶囊, 消栓通络胶囊, 消栓通络颗粒, 消眩止晕片, 消炎软膏, 消炎止痛膏, 蝎蚣胶囊, 心宝丸, 心脑清软胶囊, 新生化颗粒, 心舒宝胶囊, 醒脑静注射液, 杏香兔耳风, 徐长卿, 血必净注射液, 血滞通胶囊, 咽立爽口含滴丸, 咽炎片, 养肾胶囊, 养胃舒颗粒, 养血安神颗粒, 养血生发胶

		囊, 腰息痛胶囊, 野木瓜片, 野苏胶囊, 乙肝扶正胶囊, 乙肝解毒胶囊, 益津降糖胶囊, 益母康颗粒, 益气补血片, 伊痛舒注射液, 益血生胶囊, 一枝蒿伤湿祛痛膏, 银杏蜜环口服液, 银杏叶注射液, 茵栀黄注射液, 婴儿健脾颗粒, 婴儿健脾散, 鱼鳔补肾丸, 愈风宁心滴丸, 玉屏风口服液, 鱼腥草片, 孕康口服液, 孕康营养液, 镇脑宁胶囊, 正骨水, 正红花油, 正清风痛宁缓释片, 正清风痛宁注射液, 止喘灵气雾剂, 止咳丸, 止痛化症片(胶囊), 止泻保童颗粒, 止泻利颗粒, 止血胶囊, 肿痛搽剂, 肿痛气雾剂, 竹红菌素软膏, 壮骨麝香止痛膏, 紫草三黄软膏, 滋心阴颗粒

Appendix 3. Full lists of drugs by Anatomical Therapeutic Chemical Classification System (ATC) and Traditional Chinese Medicine (TCM) classification

ATC Classification	Generic name for western medicine
Alimentary Tract and Metabolism	Acarbose; Acetylsalicylic acid; Albumin tannate; Alfacalcidol; Almagate; Ascorbic acid (vit C); Atropine; Bacillus licheniformis; Beclometasone; Belladonna total alkaloids; Berberine; Betamethasone; Bismuth Potassium Citrate; Bismuth, Magnesium and Sodium Bicarbonate; Bozhi Glycopeptide; Budesonide; Bulleyaconitine A; Butylscopolamine and analgesics; Calcitriol; Calcium acetate anhydrous; Calcium carbonate; Calcium Carbonate and Vitamin D3; Calcium chloride; Calcium gluconate; Calcium lactate; Calcium phosphate; Chlorhexidine; Chlortetracycline; Cimetidine; Clotrimazole; Coated Aldehyde Oxystarch; Colloidal Bismuth Pectin; Commpound Vitamin B; Compound Aluminium Hydroxide; Compound Amino Acid 18AA; Compound Ammonium Glycyrrhetate; Compound Bismuth Aluminate; Compound Diisopropylamine Dichloroacetate; Compound Sodium Chloride; CompoundChlorhexidineHydrochlorideDusting; Cromoglicic acid; Cydiodine Buccal; Deoxynucleotide; Dexamethasone; Diammonium Glycyrrhizinate; Domperidone; Doxycycline; Enteral Nutritional Suspension; epalrestat; Ephedrine, combinations; Epinephrine; Ergocalciferol; Etimicin; Famotidine; Fat Emulsion, Amino Acids and Glucose; Gabexate Mesilate; Gastromucin; Glibenclamide; Gliclazide; Glipizide; Glucurolactone; Glycerine Enema or Sorbitol Enema; Glycerol; Granisetron; Hepatocyte Growth-promoting Factors; Hydrocortisone; Hydrogen peroxide; Insulin (human); Insulin aspart; Insulin detemir; Insulin glargine; Lactasin; Lactic acid producing organisms; Lactulose; Lansoprazole; Levocarnitine; L-Glutamine and Sodium Gualenate; Live Combined Bacillus Subtilis and Enterococcus Faecium; Live Combined Bifidobacterrium,Lactobacillus and Enterococcus; Magnesium sulfate; Mannitol; Medicinal charcoal; Metformin; Metoclopramide; Metronidazole; Miconazole; Miglitol; Misoprostol; Mosapride; Multienzymes (lipase, protease etc.); Multivitamins and other minerals, incl. combinations; Neomycin; Nystatin; Omeprazole; Ondansetron; Ordinary salt combinations; Pantoprazole; Penehyclidine; Phenolphthalein; Phloroglucinol; Pioglitazone; Polyene Phosphatidylcholine ; Polymyxin B; Potassium chloride; Potassium Magnesium Aspartate; Prednisone; protein-free calf blood extract; Pyridoxine (vit B6); Rabeprazole; Racecadotril; Ranitidine; Recombinant human growthhormone; Repaglinide; Riboflavin (vit B2); Rice Bran Sterol; Scopolamine; Silibinin; Silybin Meglumine; Smectite; Sodium Glucuronic Acid; Sodium Lactate Ringer's; Streptomycin; Sucralfate; Sulfasalazine; Tetracycline; Thiamine (vit B1); Thymalfasin; Tocopherol (vit E); Triamcinolone; Tripterysium Glycosides; Tropisetron; Ursodeoxycholic acid; Vancomycin; Vitamin U,Belladonna and Aluminium Capsules; Zinc gluconate
Blood and Blood Forming Organs	Acetylsalicylic acid; Aescinate; Alanyl glutamine; Amino acids; Aminomethylbenzoic acid; Batilol; Calcium chloride; Carbamide; Carbazochrome; Chlorhexidine; Chymotrypsin; Clopidogrel; Coated Aldehyde Oxystarch; Coenzyme A; Combinations of electrolytes; Compound Amino Acid 18AA; Compound Sodium Chloride; CompoundChlorhexidineHydrochlorideDusting; Cyanocobalamin; Cyclic adenosine monophosphate; Daidzein; Deoxynucleotide; Desoxyribonuclease; Dextran; Dextriferron; Dipyridamole; Edaravone; Epinephrine; Erythropoietin; Etamsylate; Ethacridine lactate; Ethylenediamine Diaceturate; Extract of Horse Chestnut Seeds; Fat Emulsion, Amino

	Acids and Glucose; Fat emulsions; Ferrous fumarate; Ferrous gluconate; Ferrous Lactate; Ferrous succinate; Ferulate; Fibrinolysin; Flavonihippopha; Folic acid; Ginkgo Leaf Extract and Dipyridamole; Haemocoagulase Atrox; Heparin; Hydroxyethylstarch; Iron, multivitamins and minerals; Iron, vitamin B12 and folic acid; Leucogen; Levamlodipine; Ligustrazine; Magnesium sulfate; Mannatide; Mannitol; Mecobalamin; Menadione; Microspheres of human albumin; Mucopolysaccharide Polysulfate; Muscular Amino Acids and Peptides and Nucleosides; Nattokinase; Neomycin; Ozagrel; Phytomenadione; Potassium chloride; protein-free calf blood extract; Recombinant Human Granulocyte Colony-Stimulating Factor Injection; Recombinant Human Granulocyte Colony-Stimulating Factor Injection; Rivaroxaban; Saccharated iron oxide; Sarpogrelate; Sodium bicarbonate; Sodium Lactate Ringer's; Streptokinase; Succinylated Gelatin; Sulfotanshinone Sodium; Thrombin; Tranexamic acid; Urokinase; Warfarin
Cardiovascular System	Adenosine; Amiodarone; Amlodipine; Atorvastatin; Benazepril; Betamethasone; Bismuth preparations, combinations; Bisoprolol; Calcium dobesilate; Candesartan; Captopril; Carvedilol; Cattle Encephalon Glycoside and Ignotin; Cyclic adenosine monophosphate; Deslanoside; Dexamethasone; Digoxin; Diltiazem; Dobutamine; Dopamine; Edaravone; Enalapril; Epinephrine; Esmolol; Felodipine; Fenofibrate; Ferulate; Flavonihippopha; Fluocinolone acetonide; Fluvastatin; Fructose 1,6-diphosphate; Furosemide; Gemfibrozil; Ginkgo Leaf Extract and Dipyridamole; Glyceryl trinitrate; Heparin; Hong Hua Huang Se Su ; Hydrochlorothiazide; Hydrocortisone; Ibuprofen; Indapamide; Indometacin; Invert sugar; Irbesartan; Isoprenaline; Isosorbide dinitrate; Isosorbide mononitrate; Labetalol; Lacidipine; Lidocaine; Losartan; Lovastatin; Metaraminol; Metoprolol; Mexiletine; Milrinone; Nattokinase; Nicergoline; Nifedipine; Nimodipine; Nitrendipine; Nitroprusside; Nitroprusside sodium; Norepinephrine; Olmesartan medoxomil; Perindopril; Phentolamine; Procaine; Propafenone; Propranolol; protein-free calf blood extract; Reserpine; Reserpine and diuretics, combinations with other drugs; Reserpine, combinations; Rosuvastatin; Sarpogrelate; Simvastatin; Spironolactone; Steroidal saponins; Telmisartan; Tetracaine; Timolol; Trimetazidine; Troxerutin; Urapidil; Valsartan; Valsartan and amlodipine; Verapamil
Dermatologicals	Aciclovir; Amikacin; Beclometasone; Benzoyl peroxide; Betamethasone; Budesonide; Butenafine; Calcitriol; Calcium gluconate; Carbamide; Chloramphenicol; Chlorhexidine; Chlortetracycline; Clindamycin; Clotrimazole; Compound Carraghenate; Compound Ketoconazole; CompoundChlorhexidineHydrochlorideDusting; Cromoglicic acid; Desonide; Dexamethasone; Diclofenac; Diphenhydramine; Econazole; Erythromycin; Erythromycin, combinations; Ethacridine lactate; Finasteride; Fluconazole; Fusidic acid; Gentamicin; Griseofulvin; Halcinonide; Human Epidermal Growth Factor; Hydrocortisone; Hydrogen peroxide; Ichtasol; Inosine; Lidocaine; Magnesium sulfate; Methylprednisolone; Metronidazole; Miconazole; Mouse Nerve Growth Factor; Mupirocin; Neomycin; Nystatin; Oxybuprocaine; Oxytetracycline; Policresulen; Potassium permanganate; protein-free calf blood extract; Recombinant Bovine Basic Fibroblast Grow; Sertaconazole; Sodium hypochlorite; Sulfur; Tacrolimus; Tetracaine; Tetracycline; Triamcinolone
Genito Urinary System and Sex Hormones	Ascorbic acid (vit C); Calcium chloride; Carboprost; Chloramphenicol; Clindamycin; Clomifene; Clotrimazole; Compound Ketoconazole; Conjugated estrogens; Cyproterone and estrogen; Desogestrel and estrogen; Diethylstilbestrol; Econazole; Estradiol; Finasteride; Furazolidone; Ibuprofen; Inosine; Levornidazole; Medroxyprogesterone; Metronidazole; Metronidazole,Clotrimazole and Chlorhexidine Acetate Suppositories;

	Miconazole; Mifepristone; Misoprostol; Naproxen; Nifuratel; Nystatin; Nystatin, combinations; Ornidazole; Oxytetracycline; Phentolamine; Policresulen; Progesterone; Recombinant human growthhormone; Tamsulosin; Terazosin; Testosterone
Systemic Hormonal Preparations, Excl. Sex Hormones and Insulins	Betamethasone; Cortisone; Desmopressin; Dexamethasone; Elcatonin; Hydrocortisone; Levothyroxine sodium; Methylprednisolone; Octreotide; Oxytocin; Posterior Pituitary Injection; Prednisone; Propylthiouracil; Somatostatin; Thiamazole; Thyroid gland preparations; Triamcinolone
Antiinfectives For Systemic Use	Abacavir; Aciclovir; Adefovir dipivoxil; Amikacin; Amoxicillim and Sulbactam; Amoxicillin; Amoxicillin and Clavulanate Potassium; Ampicillin; Ampicillin, combinations; Azlocillin; Aztreonam; Benzylpenicillin; Cefaclor; Cefadroxil; Cefalexin; Cefaloridine; Cefathiamidine; Cefazolin; Cefepime; Cefixime; Cefmetazole; Cefoperazone, combinations; Cefotaxime; Cefpiramide; Cefradine; Ceftazidime; Ceftizoxime; Ceftriaxone; Cefuroxime; Chloramphenicol; Chlortetracycline; Ciprofloxacin; Clarithromycin; Clindamycin; Compound Ammonium Glycyrrhetate; Compound Ketoconazole; Deoxynucleotide; Efavirenz; Entecavir; Erythromycin; Ethambutol; Etimicin; Fleroxacin; Fluconazole; Foscarnet; Fosfomycin; Fusidic acid; Ganciclovir; Gatifloxacin; Gentamicin; Immunoglobulins, normal human, for intravascular adm.; Isoniazid; Itraconazole; Kitasamycin; Lamivudine; Levofloxacin; Levornidazole; Lincomycin; Lomefloxacin; Lopinavir/Ritonavir; Mannatide; Meropenem; Methenamine; Metronidazole; Mezlocillin; Miconazole; Midecamycin; Moroxydine; Moxifloxacin; Neomycin; Netilmicin; Nevirapine; Nitrofurantoin; Norfloxacin; Ofloxacin; Ornidazole; Oxacillin; Oxytetracycline; Pazufloxacin; Pefloxacin; Phenoxymethylpenicillin; Piperacillin; Polymyxin B; Pyrazinamide; Recombinant human growthhormone; Ribavirin; Rifampicin; Rifamycin; Roxithromycin; Spectinomycin; Spiramycin; Stavudine; Streptomycin; Sulfamethoxazole; Sulfamethoxazole and trimethoprim; Tenofovirdisoproxil; Tetanus antitoxin; Tetanus immunoglobulin; Tetracycline; Thiamphenicol; Thymalfasin; Thymopetidum; Tinidazole; Tobramycin; Transfer Factor; Tripterysium Glycosides; Vancomycin; Vidarabine; Zidovudine
Antineoplastic and Immunomodulating Agents	Azathioprine; BCG vaccine; Bozhi Glycopeptide; Capecitabine; Celecoxib; Ciclosporin; Cisplatin; Cyclophosphamide; Diethylstilbestrol; Disodium Cantharidinate; Doxorubicin; Epirubicin; Fluorouracil; Gemcitabine; Interleukin; Leflunomide; Leucogen; Mannatide; Medroxyprogesterone; Methotrexate; Mitomycin; Mycophenolate Mofetil; Oxaliplatin; Paclitaxel; Peginterferon alfa-2b; Pidotimod; Poly I:C; Recombinant Human Granulocyte Colony-Stimulating Factor Injection; Sirolimus; Tacrolimus; Tamoxifen; Tegafur; Tegafur, combinations; Temozolomide; Thalidomide; Ubenimex; Vitamin B4
Musculo-Skeletal System	Aceclofenac; Alendronic acid; Allopurinol; Atracurium; Benzbromarone; Bulleyaconitine A; Celecoxib; Cervus and Cucumis Polypeptide; Cervus Nippon Temmick; Chondroitine; Colchicine; Dexibuprofen; Diacerein; Diclofenac; Etoricoxib; Febuxostat; Fenbufen; Glucosamine; Glucurolactone; Ibuprofen; Ibuprofen, combinations; Indometacin; Lornoxicam; Loxoprofen; Meloxicam; Mucopolysaccharide Polysulfate; Nabumetone; Naproxen; Parecoxib;

	Phenylbutazone; Quinine, combinations with psycholeptics; Salcatonin; Sodium Hyaluronate; Suxamethonium; Total glucosides of paeonia; Tripterysium Glycosides; Trypsin, combinations; Vecuronium
Nervous System	Acetagastrodin; Acetylsalicylic acid; Aescinate; Alprazolam; Aminophenazone, combinations excl. psycholeptics; Anisodamine; Aripiprazole; Betahistine; Bucinnazine; Bulleyaconitine A; Bupivacaine; Buspirone; Carbachol; Carbamazepine; Cattle Encephalon Glycoside and Ignotin; Cerebroprotein; Chlorpromazine; Citicoline; Clozapine; Codeine, combinations excl. psycholeptics; Combinations of barbiturates; Dexketoprofen tromethamine; Dexmedetomidine; Dezocine; Diazepam; Difenidol; Doxepin; Droperidol; Enflurane; epalrestat; Escitalopram; Estazolam; Etomidate; Fentanyl; Flunarizine; Fluoxetine; Gabapentin; Galantamine; Gastrodin; Haloperidol; Huperzine A; Isoflurane; Ketamine; Levodopa and Benserazide Hydrochloride; Lidocaine; Lidocaine, combinations; Lithium; Loxoprofen; Lysine,Inosite and Vitamin B12; Meclofenoxate; Melitracen and psycholeptics; Metamizole sodium; Midazolam; Mirtazapine; Morphine; Mouse Nerve Growth Factor; Muscular Amino Acids and Peptides and Nucleosides; Neostigmine; Olanzapine; Oryzanol; Oxiracetam; Paracetamol, combinations excl. psycholeptics; Paroxetine; Penfluridol; Perphenazine; Pethidine; Phenobarbital; Phenytoin; Pilocarpine; Piracetam; Pregabalin; Procaine; Procaine, combinations; Propofol; protein-free calf blood extract; Quetiapine; Remifentanil; Risperidone; Ropivacaine; Scopolamine; Sertraline; Sevoflurane; Sufentanil; Sulpiride; Tetracaine; Tramadol; Trihexyphenidyl; Valproic acid; Venlafaxine; Vinpocetine
Antiparasitic Products, Insecticides and Repellents	Albendazole; Hydroxychloroquine; Levamisole; Levornidazole; Lindane; Metronidazole; Ornidazole; Tinidazole
Respiratory System	Acetylcysteine; Ambroxol; Ambroxol Hydrochloride and Clenbuterol Hydrochloride; Aminophylline; Amoxicillim and Sulbactam; Amoxicillin and Clavulanate Potassium; Beclometasone; BERGENINI COMPOSITAE TABLETS; Betamethasone; Budesonide; Calcium Chloride and Sodium Bromide; Carbocisteine; Cetirizine; Chlorhexidine; Chlorphenamine; Codeine; Compound Liquorice; Cromoglicic acid; Cyproheptadine; Cytochrome C; Desloratadine; Dexamethasone; Dexamethasone, combinations; Dextromethorphan; Dimefline; Diphenhydramine; Doxofylline; Ebastine; Ephedrine; Epinephrine; Formoterol; Ipratropium bromide; Isoprenaline; Lidocaine; Lobeline; Loratadine; Mannatide; Montelukast; Mupirocin; Naphazoline; Neomycin; Nikethamide; Pentoxyverine; Pholcodine; Poractant Alfa; Potassium Dehydroandrograpolide Succinate; Promethazine; Pseudoephedrine, combinations; Salbutamol; Terbutaline; Terfenadine; Tiopronin; Tiotropium bromide; Triamcinolone
Sensory Organs	Acetylcysteine; Aciclovir; Amikacin; Amiotide; Ampicillin; Ascorbic acid (vit C); Atropine; Azithromycin; Betamethasone; Carbachol; Chloramphenicol; Chlorhexidine; Chlortetracycline; Chymotrypsin; Ciprofloxacin; Cortisone; Cromoglicic acid; Desonide; Dexamethasone; Diclofenac; Ephedrine; Epinephrine; Erythromycin; Fluocinolone acetonide; Fusidic acid; Ganciclovir; Gatifloxacin; Gentamicin; Heparin; Hydrobenzole; Hydrocortisone; Hydrogen peroxide; Indometacin; Inosine; iodizedlecithin; Levofloxacin; Lidocaine; Lomefloxacin; Miconazole;

	Micronomicin; Moxifloxacin; Naphazoline; Naphazoline, combinations; Neomycin; Neostigmine; Netilmicin; Norfloxacin; Ofloxacin; Oxybuprocaine; Oxytetracycline; Pilocarpine; Pirenoxine; Polymyxin B; Procaine; Rifamycin; Scopolamine; Tetracaine; Tetracycline; Timolol; Tobramycin; Triamcinolone; Tropicamide; Tropicamide, combinations; Vidarabine
TCM classification	Pinyin for TCMs
Internal medicine	An Gong Niu Huang Wan ; An Shen Bu Nao Ye ; An Shen Bu Xin Wan ; Bai Ji Ke Li (Fen 、Tang Jiang); Bai Jie Jiao Nang ; Bai Le Mian Jiao Nang ; Bai Ling Jiao Nang ; Bai Zi Yang Xin Wan ; Ban Lan Gen Ke Li ; Ban Lan Gen Pian ; Ban Lan Gen Zhen ; Ban Lan Qing Re Ke Li ; Ban Xia Tang Jiang ; Bao Bao Yi Tie Ling ; Bao Fei Ning Sou Ke Li ; Bao Ji Wan (Kou Fu Ye); Bu Zhang Nao Xin Tong Jiao Nang ; Bu Zhong Yi Qi Wan (Ke Li); Can Fu Zhu She Ye ; Can Ling Bai Shu San (Wan 、Ke Li); Can Ling Jian Pi Wei Ke Li ; Can Mai Zhu She Ye ; Can Qi Jiang Tang Pian /Jiao Nang ; Can Qi Wu Wei Zi Tang Jiang ; Can Song Yang Xin Jiao Nang ; Can Xian Sheng Mo Kou Fu Ye ; Can Xiong Pu Tao Tang Zhu She Ye ; Chai Hu Zhu She Ye ; Chang Qing Cha ; Chang Yan Ning Jiao Nang (Pian); Chen Xiang Lu Bai Lu Pian ; Chuan Bei Pi Pa Gao ; Chuan Bei Pi Pa Tang Jiang ; Chuan Ke Zhi Zhu She Ye ; Chuan Xin Lian Zhu She Ye ; Ci Wu Jia Nao Ling Ye ; Ci Wu Jia Pian ; Da Huo Luo Wan (Jiao Nang); Da Li Tong Ke Li ; Dan Can Zhu She Ye (Dong Gan /Fen Zhen); Dan Deng Tong Nao Jiao Nang (Ruan Jiao Nang); Dan Hong Zhu She Ye ; Dan Shi Li Tong Pian ; Dan Shi Tong Jiao Nang ; Dan Shu Jiao Nang ; Dang Gui Nian Tong Ke Li ; Dao Chi Pian ; Deng Tai Ye Ke Li ; Deng Zhan Hua Su Pian ; Deng Zhan Hua Su Zhu She Ye ; Deng Zhan Sheng Mo Jiao Nang ; Deng Zhan Xi Xin He Ji ; Deng Zhan Xi Xin Ke Li ; Deng Zhan Xi Xin Zhu She Ye ; Di Yu Sheng Bai Pian ; Dian Xian Ning Pian ; Ding Gui Er Qi Tie ; Du Yi Wei Ruan Jiao Nang ; E Shu You Zhu She Ye ; Fei Li Ke He Ji Jiao Nang ; Feng Han Gan Mao Ke Li ; Feng Liao Chang Wei Kang Ke Li ; Feng Re Gan Mao Ke Li ; Feng Shi Ding Jiao Nang ; Feng Shi Ning Zhu She Ye ; Fu Fa Xian He Cao Chang Yan Jiao Nang ; Fu Fang A Jiao Jiang ; Fu Fang Chuan Xin Lian Pian ; Fu Fang Dan Can Pian (Ke Li 、Jiao Nang 、Di Wan); Fu Fang Dang Gui Zhu She Ye ; Fu Fang Feng Shi Ning Jiao Nang (Pian); Fu Fang Long Xue Jie Jiao Nang ; Fu Fang Luo Bu Ma Pian ; Fu Fang Nan Ban Lan Gen Pian ; Fu Fang Yu Xing Cao Tang Jiang ; Fu Ling Bai Shu Ke Li ; Gan Kang Pian ; Gan Mao Di Wan ; Gan Mao Ling (Ke Li 、Chong Ji 、Jiao Nang 、Pian); Gan Mao Qing Pian /Jiao Nang ; Gan Mao Qing Re Ke Li (Jiao Nang); Gan Mao Shu Feng Pian (Ke Li 、Wan); Gan Mao Xiao Yan Pian ; Gan Mao Zhi Ke Tang Jiang ; Gan Wei Qi Tong San ; Gan Zhi Xiao Jiao Nang ; Gong Lao Qu Huo Jiao Nang ; Gu Can Chang An Jiao Nang ; Gu Long Jiao Nang ; Guan Xin Ning Zhu She Ye ; Guan Xin Shu Tong Jiao Nang ; Gui Fu Li Zhong Wan ; Gui Pi Wan (He Ji); Ha Jie Ding Chuan Jiao Nang ; Hai Kun Shen Xi Jiao Nang ; Han Chuan Zu Pa Ke Li ; Hong Hua Huang Se Su ; Hong Hua Zhu She Ye ; Hong Hui Xiang Zhu She Ye ; Hou Er Huan Xiao Yan Ke Li ; Hu Gan Pian (Ke Li 、Jiao Nang); Hu Li San Jiao Nang ; Hua Ji Kou Fu Ye ; Hua Tan Li Yan Huang Shi Xiang Sheng Wan ; Hua Tuo Zai Zao Wan ; Huang Lian Shang Qing Wan (Ke Li 、Jiao Nang 、Pian); Huang Qi Ke Li ; Huang Qi Sheng Mo Yin ; Huang Qi Zhu She Ye ; Huang Teng Su Pian (Fen San Pian 、Jiao Nang 、Ruan Jiao Nang); Huang Teng Su Zhu She Ye ; Huo Xiang Zheng Qi Jiao Nang ; Huo Xiang Zheng Qi Shui (Kou Fu Ye 、Ruan

	Jiao Nang，Ke Li); Ji Zhi Tang Jiang (Ke Li); Jian Er Qing Jie Ye ; Jian Pi Yang Gan Wan ; Jian Wei Xiao Shi Pian ; Jian Yang Jiao Nang ; Jiang Tang Wan ; Jie Jiu Ling Kou Fu Ye ; Jie Yu An Shen Ke Li ; Jin Kui Shen Qi Wan (Pian); Jin Lian Wei Shu Pian ; Jin Ma Gan Tai Ke Li ; Jin Shui Bao Jiao Nang ; Jin Zhen Kou Fu Ye ; Jin Zhi Jie Yin Han Shu Ye ; Jing Dou Nian Ci Yan Mi Lian Chuan Bei Pi Pa Gao ; Ju Geng Ma Huang Jian Tang Jiang ; Ju Hong Wan (Ke Li 、Jiao Nang 、Pian); Juan Bi Ke Li ; Kang Ai Zhu She Ye ; Kang Bing Du He Ji ; Kang Bing Du Kou Fu Ye (Ke Li); Kang Gan Ke Li ; Kang Gan Ling Pian ; Ke Ke Jiao Nang ; Ke Ke Tang Jiang ; Ke Lu Kou Fu Ye ; Ke Su Ting Tang Jiang ; Kou Qiang Yan Pen Wu Ji ; Ku Die Zi Zhu She Ye ; Kui Hua Hu Gan Pian ; Kui Hua Wei Kang Ling ; Lan Qin Kou Fu Ye ; Li Dan Zhi Tong Jiao Nang /Pian ; Li Xu Wang Xue Sai Tong Ruan Jiao Nang ; Lian Hua Qing Wen Jiao Nang ; Lian Hua Qing Wen Jiao Nang (Ke Li); Ling Zhi Yi Shou Jiao Nang ; Liu Jing Tou Tong Pian ; Liu Ling Wan ; Liu Wei Di Huang Wan (Ke Li 、Jiao Nang); Ma Ren Run Chang Wan (Ruan Jiao Nang); Mo Luo Ning Zhu She Ye ; Mo Xue Kang Jiao Nang ; Mu Ru Duo Ke Li ; Nao Li Qing Wan ; Nao Luo Tong Jiao Nang ; Nao Xin Shu Kou Fu Ye ; Nao Xin Tong Wan (Jiao Nang 、Pian); Niao Du Qing Ke Li ; Niao Gan Ning Ke Li ; Ning Xin Bao Jiao Nang ; Niu Huang Jie Du Wan (Jiao Nang 、Ruan Jiao Nang 、Pian); Niu Huang Shang Qing Wan (Jiao Nang 、Pian); Pai Du Yang Yan Jiao Nang ; Pu Le An Jiao Nang (Pian); Qi Li Qiang Xin Jiao Nang ; Qi Ye Shen An Pian ; Qi Zhi Wei Tong Ke Li (Pian); Qian Bai Bi Yan Pian ; Qian Lie Ci Sheng Tie ; Qian Lie Shu Tong Jiao Nang ; Qian Zi Hong Ke Li ; Qiang Li Nao Qing Su Pian ; Qiang Li Pi Pa Lu ; Qin Qiao Kou Fu Ye ; Qing Fei Hua Tan Wan ; Qing Fei Yi Huo Pian ; Qing Hou Li Yan Ke Li ; Qing Hou Yan Ke Li ; Qing Huo Zhi Mai Pian ; Qing Kai Ling Ke Li (Jiao Nang 、Pian 、Zhu She Ye); Qing Liang You ; Qing Re Jie Du Kou Fu Ye ; Qing Re San Jie Pian ; Qing Re Zhi Ke Ke Li ; Qing Wei Huang Lian Wan ; Qing Xuan Zhi Ke Ke Li ; Qiu Xie Ling He Ji ; Qu Feng Zhi Tong Jiao Nang ; Re Du Ning Zhu She Ye ; Re Du Qing Pian ; Ren Can Feng Wang Jiang ; Ren Can Zai Zao Wan ; Run Chang Jiao Nang ; Run Zao Zhi Yang Jiao Nang ; San Huang Pian ; San Jin Pian ; San Jiu Wei Tai Ke Li (Jiao Nang); San Qi Pian ; Sang Ju Gan Mao Pian ; Se Chang Zhi Xie San ; Sha Ji Gan Ru Ji ; Sha Qi Wan ; Shan Zha Ke Li ; Shao Bei Zhu She Ye ; She Dan Chen Pi Kou Fu Ye ; She Dan Chuan Bei Ye ; She Xiang Bao Xin Wan ; Shen Bao He Ji ; Shen Bao Tang Jiang ; Shen Shuai Ning Jiao Nang ; Sheng Mo Yin (Ke Li 、Jiao Nang 、Zhu She Ye); Shi Di Shui ; Shi Du Qing Jiao Nang ; Shi Lin Tong Ke Li ; Shu Gan Ke Li ; Shu Mi Tong Jiao Nang ; Shu Xin Jiang Zhi Pian ; Shu Xin Tong Mo Jiao Nang ; Shu Xue Ning Zhu She Ye ; Shu Xue Tong Zhu She Ye ; Shuang Huang Lian He Ji (Kou Fu Ye 、Ke Li 、Jiao Nang 、Pian); Shuang Huang Lian Zhu She Ye ; Si Ji Gan Mao Jiao Nang ; Si Mo Tang Kou Fu Ye ; Si Shen Wan (Pian); Su Xiao Jiu Xin Wan ; Tan Ke Jing San (Pian); Tan Re Qing Zhu She Ye ; Tian He Zhui Feng Gao ; Tian Ma Tou Tong Pian ; Tian Ma Wan ; Tian Ma Xing Nao Jiao Nang ; Tian Qi Hua Ye Ke Li ; Tian Wang Bu Xin Wan (Pian); Tong Bian Ling Jiao Nang ; Tong Feng Shu Pian ; Tong Feng Xiao Ke Li ; Tong Shu Jiao Nang ; Tong Xin Luo Jiao Nang ; Tong Xuan Li Fei Wan (Ke Li 、Jiao Nang 、Pian); Tou Tong Ning Jiao Nang ; Wang Bi Ke Li (Jiao Nang 、Pian); Wei Cyin Qiao Pian ; Wei Fu Chun Pian ; Wei Kang Ling Jiao Nang ; Wei Su Ke Li ; Wen Pi Gu Chang San ; Wen Wei Shu Ke Li ; Wen Xin Ke Li ; Wu Ji Bai Feng Wan (Jiao Nang 、Pian); Wu Lin Hua Shi Wan ; Wu Zhi Jiao Nang ; Xi Gua Shuang Pian ; Xi Yan Ping Zhu

	She Ye ; Xian Zhu Li Kou Fu Ye ; Xiang Dan Zhu She Ye ; Xiang Guo Jian Xiao Pian ; Xiang Lian Pian ; Xiang Sha Ping Wei Wan (Ke Li); Xiang Sha Wei Tong San ; Xiao Chai Hu Ke Li ; Xiao Er Bao Tai Kang Ke Li ; Xiao Er Chang Wei Kang Ke Li ; Xiao Er Fei Re Ke Chuan Kou Fu Ye ; Xiao Er Hua Du San (Jiao Nang); Xiao Er Hua Tan Zhi Ke Ke Li ; Xiao Er Ke Chuan Ling Ke Li ; Xiao Er Qing Yan Ke Li ; Xiao Er Re Su Qing Kou Fu Ye (Ke Li); Xiao Er Xiao Ji Zhi Ke Kou Fu Ye ; Xiao Er Yan Bian Ke Li ; Xiao Ke Chuan Tang Jiang ; Xiao Ke Wan ; Xiao Shuan Tong Luo Jiao Nang ; Xiao Shuan Tong Luo Ke Li ; Xiao Xuan Zhi Yun Pian ; Xiao Yao Wan (Ke Li); Xie Gong Jiao Nang ; Xin Bao Wan ; Xin Fu Fang Da Qing Ye Pian ; Xin Ke Shu Jiao Nang (Pian); Xin Nao Qing Ruan Jiao Nang ; Xin Shu Bao Jiao Nang ; Xing Nao Jing Zhu She Ye ; Xing Pi Yang Er Ke Li ; Xing Xiang Tu Er Feng ; Xu Zhang Qing ; Xue Bi Jing Zhu She Ye ; Xue Sai Tong Jiao Nang (Pian); Xue Sai Tong Zhu She Ye (Dong Gan); Xue Zhi Kang Pian (Jiao Nang); Xue Zhi Tong Jiao Nang ; Yang Shen Jiao Nang ; Yang Wei Shu Ke Li ; Yang Xue An Shen Ke Li ; Yang Xue Qing Nao Wan (Ke Li); Yang Xue Sheng Fa Jiao Nang ; Ye Mu Gua Pian ; Ye Su Jiao Nang ; Yi Gan Fu Zheng Jiao Nang ; Yi Gan Jie Du Jiao Nang ; Yi Jin Jiang Tang Jiao Nang ; Yi Mo Kang Pian ; Yi Qi Bu Xue Pian ; Yi Tong Shu Zhu She Ye ; Yi Xin Shu Ke Li (Jiao Nang 、Pian); Yi Xue Sheng Jiao Nang ; Yi Zhi Hao Shang Shi Qu Tong Gao ; Yin Dan Xin Nao Tong Ruan Jiao Nang ; Yin Huang Kou Fu Ye (Ke Li 、Jiao Nang 、Pian); Yin Qiao Jie Du Wan (Ke Li 、Jiao Nang 、Ruan Jiao Nang 、Pian); Yin Xing Mi Huan Kou Fu Ye ; Yin Xing Ye Jiao Nang (Pian 、Di Wan); Yin Xing Ye Zhu She Ye ; Yin Zhi Huang Kou Fu Ye (Ke Li); Yin Zhi Huang Zhu She Ye ; Ying Er Jian Pi Ke Li ; Ying Er Jian Pi San ; Yu Biao Bu Shen Wan ; Yu Feng Ning Xin Di Wan ; Yu Ping Feng Ke Li ; Yu Ping Feng Kou Fu Ye ; Yu Xing Cao Pian ; Yuan Hu Zhi Tong Pian (Ke Li 、Jiao Nang 、Di Wan); Yun Tong Ding Jiao Nang ; Zao Ren An Shen Ke Li (Jiao Nang); Zhen Nao Ning Jiao Nang ; Zheng Qing Feng Tong Ning Huan Shi Pian ; Zheng Qing Feng Tong Ning Zhu She Ye ; Zheng Tian Wan (Jiao Nang); Zhi Bai Di Huang Wan ; Zhi Chuan Ling Qi Wu Ji ; Zhi Ke Wan ; Zhi Xie Bao Tong Ke Li ; Zhi Xie Li Ke Li ; Zhi Xue Jiao Nang ; Zhui Feng Tou Gu Wan ; Zi Xin Yin Ke Li ; Zu Shi Ma Guan Jie Zhi Tong ; Zu Shi Ma Zhu She Ye<br><br>Chinese name:安宫牛黄丸; 安神补脑液; 安神补心丸; 白芨颗粒（粉、糖浆）; 百解胶囊; 百乐眠胶囊; 百令胶囊; 柏子养心丸; 板蓝根颗粒; 板蓝根片; 板蓝根针; 板蓝清热颗粒; 半夏糖浆; 宝宝一贴灵; 保肺宁嗽颗粒; 保济丸（口服液）; 步长脑心通胶囊; 补中益气丸（颗粒）; 参附注射液; 参苓白术散（丸、颗粒）; 参苓健脾胃颗粒; 参麦注射液; 参芪降糖片/胶囊; 参芪五味子糖浆; 参松养心胶囊; 参仙升脉口服夜; 参芎葡萄糖注射液; 柴胡注射液; 肠清茶; 肠炎宁胶囊（片）; 陈香露白露片; 川贝枇杷膏; 川贝枇杷糖浆; 喘可治注射液; 穿心莲注射液; 刺五加脑灵液; 刺五加片; 大活络丸（胶囊）; 达立通颗粒; 丹参注射液（冻干/粉针）; 丹灯通脑胶囊（软胶囊）; 丹红注射液; 胆石利通片; 胆石通胶囊; 胆舒胶囊; 当归拈痛颗粒; 导赤片; 灯台叶颗粒; 灯盏花素片; 灯盏花素注射液; 灯盏生脉胶囊; 灯盏细辛合剂; 灯盏细辛颗粒; 灯盏细辛注射液; 地榆升白片; 癫痫宁片; 丁桂儿脐贴; 独一味软胶囊; 莪术油注射液; 肺力咳合剂胶囊; 风寒感冒颗粒; 枫蓼肠胃康颗粒; 风热感冒颗粒; 风湿定胶囊; 风湿宁注射液; 复发仙鹤草肠炎胶囊; 复方阿胶浆; 复方穿心莲片; 复方丹参片（颗粒、胶囊、滴丸）; 复方当归注射液; 复方风湿宁胶囊（片）; 复方龙血竭胶囊; 复方罗布麻片; 复方南板蓝根片; 复方鱼腥草糖浆; 茯苓白术颗粒; 肝康片; 感冒滴丸; 感冒灵（颗粒、冲剂、胶囊、片）; 感冒清片/胶囊; 感冒清热颗粒（胶囊）; 感冒疏风片（颗粒、丸）; 感冒消

	炎片; 感冒止咳糖浆; 肝胃气痛散; 肝脂消胶囊; 功劳去火胶囊; 谷参肠安胶囊; 骨龙胶囊; 冠心宁注射液; 冠心舒通胶囊; 桂附理中丸; 归脾丸（合剂）; 蛤蚧定喘胶囊; 海昆肾喜胶囊; 寒喘祖帕颗粒; 红花黄色素; 红花注射液; 红茴香注射液; 猴耳环消炎颗粒; 护肝片（颗粒、胶囊）; 虎力散胶囊; 化积口服液; 化痰利咽黄氏响声丸; 华佗再造丸; 黄连上清丸（颗粒、胶囊、片）; 黄芪颗粒; 黄芪生脉饮; 黄芪注射液; 黄藤素片（分散片、胶囊、软胶囊）; 黄藤素注射液; 霍香正气胶囊; 藿香正气水（口服液、软胶囊，颗粒）; 急支糖浆（颗粒）; 健儿清解液; 健脾养肝丸; 健胃消食片; 健阳胶囊; 降糖丸; 解酒灵口服液; 解郁安神颗粒; 金匮肾气丸（片）; 金莲胃舒片; 金马肝泰颗粒; 金水宝胶囊; 金振口服液; 金栀洁龈含漱液; 京都念慈菴蜜炼川贝枇杷膏; 桔梗麻黄碱糖浆; 橘红丸（颗粒、胶囊、片）; 蠲痹颗粒; 康艾注射液; 抗病毒合剂; 抗病毒口服液（颗粒）; 抗感颗粒; 抗感灵片; 克咳胶囊; 克咳糖浆; 咳露口服液; 咳速停糖浆; 口腔炎喷雾剂; 苦碟子注射液; 葵花护肝片; 葵花胃康灵; 蓝芩口服液; 利胆止痛胶囊/片; 理洫王血塞通软胶囊; 莲花清瘟胶囊; 连花清瘟胶囊（颗粒）; 灵芝益寿胶囊; 六经头痛片; 六灵丸; 六味地黄丸（颗粒、胶囊）; 麻仁润肠丸（软胶囊）; 脉络宁注射液; 脉血康胶囊; 母乳多颗粒; 脑立清丸; 脑络通胶囊; 脑心舒口服液; 脑心通丸（胶囊、片）; 尿毒清颗粒; 尿感宁颗粒; 宁心宝胶囊; 牛黄解毒丸（胶囊、软胶囊、片）; 牛黄上清丸（胶囊、片）; 排毒养颜胶囊; 普乐安胶囊（片）; 芪苈强心胶囊; 七叶神安片; 气滞胃痛颗粒（片）; 千柏鼻炎片; 前列磁生贴; 前列舒通胶囊; 千紫红颗粒; 强力脑清素片; 强力枇杷露; 芩翘口服液; 清肺化痰丸; 清肺抑火片; 清喉利咽颗粒; 清喉咽颗粒; 清火栀麦片; 清开灵颗粒（胶囊、片、注射液）; 清凉油; 清热解毒口服液; 清热散结片; 清热止咳颗粒; 清胃黄连丸; 清宣止咳颗粒; 秋泻灵合剂; 祛风止痛胶囊; 热毒宁注射液; 热毒清片; 人参蜂王浆; 人参再造丸; 润肠胶囊; 润燥止痒胶囊; 三黄片; 三金片; 三九胃泰颗粒（胶囊）; 三七片; 桑菊感冒片; 涩肠止泻散; 沙棘干乳剂; 痧气丸; 山楂颗粒; 芎倍注射液; 蛇胆陈皮口服液; 蛇胆川贝液; 麝香保心丸; 肾宝合剂; 肾宝糖浆; 肾衰宁胶囊; 生脉饮（颗粒、胶囊、注射液）; 十滴水; 湿毒清胶囊; 石淋通颗粒; 舒肝颗粒; 舒泌通胶囊; 舒心降脂片; 舒心通脉胶囊; 舒血宁注射液; 疏血通注射液; 双黄连合剂（口服液、颗粒、胶囊、片）; 双黄莲注射液; 四季感冒胶囊; 四磨汤口服液; 四神丸（片）; 速效救心丸; 痰咳净散（片）; 痰热清注射液; 天和追风膏; 天麻头痛片; 天麻丸; 天麻醒脑胶囊; 田七花叶颗粒; 天王补心丸（片）; 通便灵胶囊; 痛风舒片; 痛风消颗粒; 通舒胶囊; 通心络胶囊; 通宣理肺丸（颗粒、胶囊、片）; 头痛宁胶囊; 尪痹颗粒（胶囊、片）; 维 C 银翘片; 胃复春片; 胃康灵胶囊; 胃苏颗粒; 温脾固肠散; 温胃舒颗粒; 稳心颗粒; 乌鸡白凤丸（胶囊、片）; 五淋化石丸; 五酯胶囊; 西瓜霜片; 喜炎平注射液; 鲜竹沥口服液; 香丹注射液; 香果健消片; 香连片; 香砂平胃丸（颗粒）; 香砂胃痛散; 小柴胡颗粒; 小儿宝泰康颗粒; 小儿肠胃康颗粒; 小儿肺热咳喘口服液; 小儿化毒散（胶囊）; 小儿化痰止咳颗粒; 小儿咳喘灵颗粒; 小儿清咽颗粒; 小儿热速清口服液（颗粒）; 小儿消积止咳口服液; 小儿咽扁颗粒; 消咳喘糖浆; 消渴丸; 消栓通络胶囊; 消栓通络颗粒; 消眩止晕片; 逍遥丸（颗粒）; 蝎蚣胶囊; 心宝丸; 新复方大青叶片; 心可舒胶囊（片）; 心脑清软胶囊; 心舒宝胶囊; 醒脑静注射液; 醒脾养儿颗粒; 杏香兔耳风; 徐长卿; 血必净注射液; 血塞通胶囊（片）; 血塞通注射液（冻干）; 血脂康片（胶囊）; 血滞通胶囊; 养肾胶囊; 养胃舒颗粒; 养血安神颗粒; 养血清脑丸（颗粒）; 养血生发胶囊; 野木瓜片; 野苏胶囊; 乙肝扶正胶囊; 乙肝解毒胶囊; 益津降糖胶囊; 益脉康片; 益气补血片; 伊痛舒注射液; 益心舒颗粒（胶囊、片）; 益血生胶囊; 一枝蒿伤湿袪痛膏; 银丹心脑通软胶囊; 银黄口服液（颗粒、胶囊、片）; 银翘解毒丸（颗粒、胶囊、软胶囊、片）; 银杏蜜环口服液; 银杏叶胶囊（片、滴丸）; 银杏叶注射液; 茵栀黄口服液（颗粒）; 茵栀黄注射液; 婴儿健脾颗粒; 婴儿健脾散; 鱼鳔补肾丸; 愈风宁心滴丸; 玉屏风颗粒; 玉屏风口服液; 鱼腥草片; 元胡止痛片（颗粒、胶囊、滴丸）; 晕痛定胶囊; 枣仁安神颗粒（胶囊）; 镇脑宁胶囊; 正清风痛宁缓释片; 正清风痛宁注射液; 正天丸（胶囊）; 知柏地黄丸; 止喘灵气雾剂; 止咳丸; 止泻保童颗粒; 止泻利颗粒; 止血胶囊; 追风透骨丸; 滋心阴颗粒; 祖师麻关节止痛; 祖师麻注射液

Surgical medecine	Bao Bao Shi Zhen Gao ; Cuo Chuang Gao ; Dong Qing Gao ; Feng You Jing ; Huang Jin Wan Hong Gao ; Ji De Sheng She Yao Pian ; Ji Yan Gao ; Jin Dan Pian ; Jin Qian Cao Ke Li ; Jin Qian Tong Lin Ke Li ; Jing Wan Hong Ruan Gao ; Kang Fu Xin Ye ; Ma Ying Long Zhi Chuang Gao ; Man Yan Shu Ning Ke Li ; Mei Bao Shi Run Shao Shang Gao ; Mo Guan Fu Kang Jiao Nang ; Pai Shi Ke Li ; She Xiang Zhi Tong Tie Gao ; She Xiang Zhui Feng Gao ; Shen Shi Tong Ke Li ; Si Huang Ruan Gao ; Song Jie You ; Xiao Yan Li Dan Pian (Ke Li 、Jiao Nang); Xiao Yan Zhi Tong Gao ; Xiao Zhong Zhi Tong Ding ; Zhu Hong Jun Su Ruan Gao<br><br>Chinese name:宝宝湿疹膏; 痤疮膏; 冬青膏; 风油精; 黄金万红膏; 季德胜蛇药片; 鸡眼膏; 金胆片; 金钱草颗粒; 金钱通淋颗粒; 京万红软膏; 康复新液; 马应龙痔疮膏; 慢严舒柠颗粒; 美宝湿润烧伤膏; 脉管复康胶囊; 排石颗粒; 麝香止痛贴膏; 麝香追风膏; 肾石通颗粒; 四黄软膏; 松节油; 消炎利胆片（颗粒、胶囊）; 消炎止痛膏; 消肿止痛酊; 竹红菌素软膏
Gynecological medicine	Ba Zhen Yi Mu Wan (Jiao Nang); Bao Fu Kang Shuan ; Bao Tai Ling ; Chuan Bai Zhi Yang Xi Ji ; Dan Bie Jiao Nang ; Dan E Fu Kang Jian Gao ; Diao Jing Cu Yun Wan ; Fu Fang Huang Song Xi Ye ; Fu Fang Sha Ji Zi You Shuan ; Fu Ke Diao Jing Pian ; Fu Ke Qian Jin Pian (Jiao Nang); Fu Ke Zai Zao Wan /Jiao Nang ; Fu Xue Kang Ke Li ; Fu Yan Jie ; Fu Yan Kang Fu Jiao Nang ; Fu Yan Kang Pian ; Fu Yan Kang Ruan Jiao Nang ; Geng Nian An Pian (Jiao Nang); Gong Liu Xiao Jiao Nang ; Gong Xue Ning Jiao Nang ; Gui Zhi Fu Ling Wan (Jiao Nang); Hong He Fu Jie Xi Ye ; Hua Hong Pian (Ke Li 、Jiao Nang); Jie Er Yin Xi Ye ; Jin Gang Teng Jiao Nang ; Jin Ji Jiao Nang ; Kang Fu Ning Jiao ; Kang Fu Yan Jiao Nang ; Kang Fu Yan Jiao Nang ; Kang Gong Yan Pian /Jiao Nang ; Ke Yang Shu Xi Ye ; Nu Bao Jiao Nang ; Nu Jin Pian ; Nuan Gong Qi Wei Wan ; Pen Yan Jing Ke Li (Jiao Nang); Ru Kang Pian ; Ru Ning Pian ; Ru Pi Xiao Ke Li (Jiao Nang 、Pian); Tong Jing Bao Ke Li ; Xiao Ru San Jie Jiao Nang ; Xin Sheng Hua Ke Li ; Yi Mu Cao Gao (Ke Li 、Jiao Nang 、Pian); Yi Mu Kang Ke Li ; Yun Kang Kou Fu Ye ; Yun Kang Ying Yang Ye ; Zhi Tong Hua Zheng Pian (Jiao Nang)<br><br>Chinese name:八珍益母丸（胶囊）; 保妇康栓; 保胎灵; 川百止痒洗剂; 丹鳖胶囊; 丹莪妇康煎膏; 调经促孕丸; 复方黄松洗液; 复方沙棘籽油栓; 妇科调经片; 妇科千金片（胶囊）; 妇科再造丸/胶囊; 妇血康颗粒; 妇炎洁; 妇炎康复胶囊; 妇炎康片; 妇炎康软胶囊; 更年安片（胶囊）; 宫瘤消胶囊; 宫血宁胶囊; 桂枝茯苓丸（胶囊）; 红核妇洁洗液; 花红片（颗粒、胶囊）; 洁尔阴洗液; 金刚藤胶囊; 金鸡胶囊; 康妇凝胶; 康妇炎胶囊; 抗妇炎胶囊; 抗宫炎片/胶囊; 克痒舒洗液; 女宝胶囊; 女金片; 暖宫七味丸; 盆炎净颗粒（胶囊）; 乳康片; 乳宁片; 乳癖消颗粒（胶囊、片）; 痛经宝颗粒; 消乳散结胶囊; 新生化颗粒; 益母草膏（颗粒、胶囊、片）; 益母康颗粒; 孕康口服液; 孕康营养液; 止痛化症片（胶囊）
Orthopedic medicine	Bu Shen Jian Gu He Ji ; Die Da Qi Li Pian ; Fu Fang Nan Xing Zhi Tong Gao ; Gou Pi Gao ; Gu Bi He Ji ; Gu Ci Ping Pian ; Gu Shang Yu He Ji ; Gu Shu Kang Jiao Nang ; Gu Yu Ling Jiao Nang ; Gu Zeng Sheng Zhen Tong Gao ; Gu Zhi Zeng Sheng Tie ; Gui Long Jin Gu Ning Pian ; Heng Gu Gu Shang Yu He Ji ; Huo Xue Zhi Tong San (Jiao Nang); Jian Zhou Yan Gao Yao ; Jie Gu Pian ; Jie Gu Qi Li Pian ; Jie Gu Xu Jin Jiao Nang ; Jing Fu Kang Ke Li ; Jing Shu Ke Li ; Kang Gu Zeng Sheng Pian ; Long Hu Pai Qing Liang You ; Long Mu Zhuang Gu Ke Li ; Long Xue Jie Jiao Nang ; Qi

	Zheng Xiao Tong Tie ; Shang Ke Die Da Pian ; Shang Ke Jie Gu Pian ; Shao Lin Feng Shi Die Da Gao ; She Xiang Zhuang Gu Gao ; Shu Jin Huo Xue Wan (Pian， Jiao Nang); Shu Jin Jian Yao Wan ; Tian He Gu Tong Gao ; Tong Luo Qu Tong Gao ; Tong Shu Jiao Nang ; Wan Tong Jin Gu Tie ; Wu Di Dan Jiao Nang ; Xian Ling Gu Bao Jiao Nang (Pian); Xiang Pi Gao ; Yao Bi Tong Jiao Nang ; Yao Xi Tong Jiao Nang ; Yong Long Zheng Hong Hua You ; Yun Nan Bai Yao (Jiao Nang 、Ke Li 、Gao 、Ding 、Qi Wu Ji); Yun Nan Hong Yao Jiao Nang ; Zheng Gu Shui ; Zheng Hong Hua You ; Zhong Tong Cha Ji ; Zhong Tong Qi Wu Ji ; Zhuang Gu She Xiang Zhi Tong Gao<br><br>Chinese name:补肾健骨合剂; 跌打七厘片; 复方南星止痛膏; 狗皮膏; 骨痹合剂; 骨刺平片; 骨伤愈合剂; 骨疏康胶囊; 骨愈灵胶囊; 骨增生镇痛膏; 骨质增生贴; 归龙筋骨宁片; 恒古骨伤愈合剂; 活血止痛散（胶囊）; 肩周炎膏药; 接骨片; 接骨七厘片; 接骨续筋胶囊; 颈复康颗粒; 颈舒颗粒; 抗骨增生片; 龙虎牌清凉油; 龙牡壮骨颗粒; 龙血竭胶囊; 奇正消痛贴; 伤科跌打片; 伤科接骨片; 少林风湿跌打膏; 麝香壮骨膏; 舒筋活血丸（片，胶囊）; 舒筋健腰丸; 天和骨通膏; 通络祛痛膏; 痛舒胶囊; 万通筋骨贴; 无敌丹胶囊; 仙灵骨葆胶囊（片）; 象皮膏; 腰痹通胶囊; 腰息痛胶囊; 永龙正红花油; 云南白药(胶囊、颗粒、膏、酊、气雾剂); 云南红药胶囊; 正骨水; 正红花油; 肿痛搽剂; 肿痛气雾剂; 壮骨麝香止痛膏
Otolaryngology medicine	Bi Yan Kang Pian ; Bing Peng San ; Er Long Zuo Ci Wan ; Fu Fang Cao Shan Hu Pian ; Fu Ma Di Bi Ye ; Jin Sang Kai Yin Wan ; Jin Sang Li Yan Wan ; Jin Sang Zi Hou Bao ; Kai Hou Jian Pen Wu Ji ; Kui Yang Ling Jiao Nang ; Tong Qiao Bi Yan Pian ; Xiang Ju Jiao Nang (Pian); Xin Qin Ke Li ; Xuan Mai Gan Ju Ke Li ; Yan Li Shuang Kou Han Di Wan ; Yan Yan Pian ; Jian Min Yan Hou Pian<br><br>Chinese name:鼻炎康片; 冰硼散; 耳聋左慈丸; 复方草珊瑚片; 呋麻滴鼻液; 金嗓开音丸; 金嗓利咽丸; 金嗓子喉宝; 开喉剑喷雾剂; 溃疡灵胶囊; 通窍鼻炎片; 香菊胶囊（片）; 辛芩颗粒; 玄麦甘桔颗粒; 咽立爽口含滴丸; 咽炎片; 健民咽喉片
Ophthalmic medicine	Chu Yi Ming Mu Pian ; Fu Fang Xue Shuan Tong Jiao Nang (Pian); Fu Ming Pian ; He Xue Ming Mu Pian ; Ming Mu Di Huang Wan ; Xiong Dan Yan Yao Shui ; Zhen Zhu Ming Mu Di Yan Ye;<br><br>Chinese name:除翳明目片; 复方血栓通胶囊（片）; 复明片; 和血明目片; 明目地黄丸; 熊胆眼药水; 珍珠明目滴眼液
Oncologic medicine	Fu Fang Ku Can Zhu She Ye ; Jin Long Jiao Nang<br><br>Chinese name:复方苦参注射液; 金龙胶囊

Appendix 4. Descriptive analysis on medicine types, sales and prices

Part I. 2009-2012

	2009				2010				2011				2012			
	Jan-Mar	Apr-Jun	Jul-Sep	Oct-Dec	Jan-Mar	Apr-Jun	Jul-Sep	Oct-Dec	Jan-Mar	Apr-Jun	Jul-Sep	Oct-Dec	Jan-Mar	Apr-Jun	Jul-Sep	Oct-Dec
	Pre-NEMP								First-stage NEMP							
**County-wide**																
Number of medicines	719	795	773	756	787	803	825	743	789	789	803	791	757	781	748	789
- Essential medicines	464	490	482	483	486	498	501	463	538	530	546	531	523	537	520	531
- Non-essential medicines	255	305	291	273	301	305	324	280	251	259	257	260	234	244	228	258
Sales (US$1,000)	233	287	298	388	437	527	541	449	511	504	602	500	636	735	897	749
- Essential medicines	140	164	193	241	281	318	336	264	329	306	377	310	400	457	555	447
- Non-essential medicines	93	123	105	147	156	208	204	185	182	199	225	190	236	278	342	302
Retail price (DPI-L)[a]	100.0	97.8	97.8	100.0	99.8	100.0	102.4	102.5	100.6	98.1	91.0	96.3	93.5	93.0	91.6	91.5
- Essential medicines	100.0	97.3	96.8	100.3	99.8	100.7	102.3	104.4	103.9	102.1	93.6	101.4	97.7	97.4	95.7	95.3
- Non-essential medicines	100.0	99.0	100.5	99.3	99.6	98.3	102.5	97.5	92.2	87.9	84.5	83.7	83.1	82.2	81.5	82.1
Retail price (DPI-F)[a]	100.0	97.8	97.6	99.6	99.6	99.1	100.9	103.0	100.7	98.7	91.5	96.2	95.2	93.9	92.6	91.8
- Essential medicines	100.0	97.3	96.6	99.8	99.8	99.6	100.8	105.0	103.5	101.8	93.3	100.1	98.7	97.7	95.8	95.6
- Non-essential medicines	100.0	98.9	100.5	99.0	98.9	97.8	101.0	97.2	92.4	89.9	86.3	85.5	85.1	82.6	83.2	81.8
Wholesale price (DPI-L)[a]	100.0	98.1	97.9	99.9	100.2	100.3	102.7	102.8	102.5	99.5	92.9	98.2	95.0	94.8	93.3	93.2
- Essential medicines	100.0	97.8	97.0	100.4	100.4	101.0	102.8	104.9	106.4	104.1	96.2	104.0	99.7	99.9	98.1	97.7
- Non-essential medicines	100.0	98.9	100.2	98.9	99.6	98.4	102.5	97.7	92.7	88.0	84.8	83.9	83.2	82.3	81.5	82.2
Wholesale price (DPI-F)[a]	100.0	98.0	97.8	99.5	99.9	99.4	101.2	103.3	102.4	100.1	93.1	97.9	96.4	95.5	93.9	93.2
- Essential medicines	100.0	97.7	96.9	99.8	100.3	99.9	101.2	105.5	105.7	103.6	95.4	102.3	100.4	99.9	97.7	97.6
- Non-essential medicines	100.0	98.8	100.2	98.7	99.0	98.0	100.9	97.4	92.8	90.0	86.6	85.8	85.2	82.7	83.2	82.0
**County hospitals (secondary care)**																
Number of medicines	565	638	599	613	642	674	658	650	637	655	647	630	663	669	630	680
- Essential medicines	390	412	395	411	410	423	417	403	417	414	419	404	434	428	409	429
- Non-essential medicines	175	226	204	202	232	251	241	247	220	241	228	226	229	241	221	251
Sales (US$1,000)	200	250	262	345	389	476	487	410	463	469	553	457	605	683	848	705
- Essential medicines	115	138	167	206	242	277	292	233	284	271	330	269	370	405	506	403
- Non-essential medicines	85	112	95	140	147	199	194	177	179	198	223	188	236	278	342	302

Retail price (DPI-L)[a]	100.0	99.1	99.3	100.6	102.2	101.5	103.6	102.1	101.7	99.7	94.6	98.0	93.6	93.4	91.7	93.1
- Essential medicines	100.0	99.0	99.0	101.4	103.6	103.5	104.2	104.3	105.8	105.2	99.4	104.6	98.7	98.9	96.7	98.5
- Non-essential medicines	100.0	99.2	99.9	98.8	99.0	97.2	102.3	97.3	92.4	87.3	84.0	82.9	82.0	81.1	80.3	81.0
Retail price (DPI-F)[a]	100.0	98.3	98.3	99.7	101.3	100.1	101.8	102.2	101.5	99.9	94.8	97.1	95.2	94.5	93.2	93.0
- Essential medicines	100.0	97.9	97.8	100.2	102.5	101.4	102.2	104.2	105.0	104.0	98.3	102.1	99.5	99.4	97.4	98.2
- Non-essential medicines	100.0	99.1	99.9	98.5	98.4	96.8	100.7	96.9	92.1	89.2	85.9	84.9	84.2	81.8	82.3	81.0
Wholesale price (DPI-L)[a]	100.0	99.3	99.2	100.4	102.4	101.7	103.8	102.3	101.5	99.6	94.7	98.1	93.7	93.6	91.8	93.4
- Essential medicines	100.0	99.4	99.1	101.3	103.9	103.7	104.5	104.5	105.6	105.1	99.4	104.8	98.9	99.2	97.0	98.8
- Non-essential medicines	100.0	99.1	99.5	98.4	99.1	97.3	102.3	97.4	92.5	87.4	84.1	83.1	82.1	81.2	80.4	81.1
Wholesale price (DPI-F)[a]	100.0	98.5	98.4	99.5	101.5	100.2	102.0	102.4	101.4	99.9	94.9	97.2	95.4	94.7	93.3	93.2
- Essential medicines	100.0	98.2	98.0	100.0	102.7	101.6	102.6	104.4	104.8	104.0	98.4	102.2	99.7	99.7	97.6	98.5
- Non-essential medicines	100.0	99.0	99.5	98.2	98.5	96.9	100.6	97.1	92.2	89.3	85.9	85.0	84.3	81.9	82.4	81.2
**THCs (primary care)**																
Number of medicines	442	463	479	431	447	428	497	348	475	418	484	473	316	318	341	321
- Essential medicines	317	325	334	303	326	315	353	271	403	384	413	400	306	314	329	309
- Non-essential medicines	125	138	145	128	121	113	144	77	72	34	71	73	10	4	12	12
Sales (US$1,000)	33	37	36	43	48	51	54	39	48	35	49	43	30	52	49	43
- Essential medicines	25	26	26	35	39	41	44	32	45	34	48	41	30	52	49	43
- Non-essential medicines	8	11	10	8	9	9	10	8	3	1	2	2	0	0	0	0
Retail price (DPI-L)[c]	100.0	100.4	101.5	105.7	106.5	107.7	119.6	122.7	101.9	94.1	95.3	92.6	91.7	88.4	89.7	90.9
Retail price (DPI-F)[c]	100.0	99.9	101.3	106.3	109.4	106.6	120.2	122.6	99.9	92.4	91.9	93.8	90.7	86.6	87.8	92.9
Wholesale price (DPI-L)[c]	100.0	100.5	101.6	105.9	108.8	109.2	121.3	125.2	115.5	106.7	108.0	104.9	103.9	100.2	101.6	103.0
Wholesale price (DPI-F)[c]	100.0	99.9	101.4	106.4	111.6	108.2	121.9	125.0	113.1	104.7	103.9	106.2	102.7	98.2	99.2	105.1

Part II. 2013-2016

	2013				2014				2015				2016			
	Jan-Mar	Apr-Jun	Jul-Sep	Oct-Dec	Jan-Mar	Apr-Jun	Jul-Sep	Oct-Dec	Jan-Mar	Apr-Jun	Jul-Sep	Oct-Dec	Jan-Mar	Apr-Jun	Jul-Sep	Oct-Dec
	First-stage NEMP												2nd stage NEMP			
**County-wide**																
Number of medicines	757	706	755	747	761	760	755	758	818	803	790	788	749	778	791	782
- Essential medicines	511	480	505	498	518	508	507	504	528	513	509	513	501	498	497	508
- Non-essential medicines	246	226	250	249	243	252	248	254	290	290	281	275	248	280	294	274

Sales (US$1,000)	1176	995	955	956	1135	896	752	777	862	939	1071	995	926	815	1072	1031
- Essential medicines	776	599	584	638	744	616	514	596	623	642	740	727	675	583	773	752
- Non-essential medicines	401	396	372	318	391	279	238	181	239	298	331	268	251	232	299	278
Retail price (DPI-L)[a]	95.1	92.9	91.6	93.3	91.0	92.5	89.5	94.1	93.9	91.4	92.5	89.5	87.4	87.9	91.9	90.7
- Essential medicines	100.3	97.8	96.3	99.1	95.5	96.9	93.2	98.8	99.2	95.7	97.2	94.7	92.8	93.3	98.2	96.7
- Non-essential medicines	82.1	80.7	79.8	79.0	79.6	81.5	80.4	82.2	80.9	80.8	80.9	76.6	73.9	74.4	76.1	75.6
Retail price (DPI-F)[a]	94.9	92.4	93.2	94.0	91.5	94.8	92.1	94.5	95.3	92.3	95.1	92.0	88.4	87.6	92.9	92.1
- Essential medicines	99.4	96.8	98.8	97.9	96.2	97.9	95.5	98.5	99.2	97.8	99.5	97.3	93.8	92.7	98.7	98.1
- Non-essential medicines	81.0	80.2	78.6	81.9	78.1	85.2	82.6	80.0	82.9	77.1	81.1	76.1	73.5	74.5	76.8	76.0
Wholesale price (DPI-L)[a]	96.4	94.2	93.0	94.8	92.6	94.2	91.2	95.8	96.0	93.1	94.4	96.4	96.9	97.4	101.8	100.5
- Essential medicines	102.2	99.8	98.5	101.2	98.0	99.5	95.5	101.3	101.6	97.7	99.4	102.1	103.2	103.7	109.3	107.6
- Non-essential medicines	81.9	80.3	79.5	78.8	79.4	81.2	80.5	82.2	82.2	82.0	82.2	82.2	81.2	81.8	83.6	83.0
Wholesale price (DPI-F)[a]	95.9	93.4	94.2	95.1	92.8	96.1	93.4	95.8	96.9	93.6	96.4	98.7	97.8	96.9	102.8	101.9
- Essential medicines	100.9	98.3	100.4	99.4	98.0	99.7	97.3	100.3	101.0	99.4	101.1	104.6	104.0	102.8	109.5	108.8
- Non-essential medicines	81.0	80.1	78.5	81.9	78.0	85.2	82.8	80.1	83.9	77.8	81.9	81.7	80.7	81.8	84.5	83.4
**County hospitals (secondary care)**																
Number of medicines	677	642	678	677	678	680	669	683	735	734	716	705	673	721	728	714
- Essential medicines	431	417	429	429	435	428	421	429	447	445	437	432	427	442	435	441
- Non-essential medicines	246	225	249	248	243	252	248	254	288	289	279	273	246	279	293	273
Sales (US$1,000)	1126	960	912	917	1084	850	708	722	809	895	1027	948	863	770	1005	967
- Essential medicines	725	563	541	599	693	570	470	542	571	599	698	682	616	540	710	692
- Non-essential medicines	401	396	372	318	391	279	238	181	238	296	330	266	247	230	295	275
Retail price (DPI-L)[a]	94.9	94.4	90.9	94.3	90.1	91.9	90.8	93.5	94.4	91.4	91.4	89.0	84.3	86.1	90.3	89.3
- Essential medicines	101.2	101.0	96.4	101.6	95.1	96.8	95.7	98.8	100.8	96.6	96.5	94.8	89.2	91.5	96.9	95.8
- Non-essential medicines	80.9	79.6	78.7	77.9	78.6	80.7	79.8	81.4	79.8	79.6	79.8	75.7	73.1	73.7	75.2	74.7
Retail price (DPI-F)[a]	95.8	94.6	93.3	95.1	92.2	94.5	93.0	94.3	96.1	93.0	94.9	91.9	87.6	87.1	92.5	91.8
- Essential medicines	101.5	100.7	100.1	100.0	97.9	98.1	97.4	98.9	100.9	99.5	99.9	98.0	93.0	92.8	98.7	98.5
- Non-essential medicines	80.3	79.5	77.9	81.1	77.4	84.6	81.7	79.2	82.5	76.5	80.0	75.1	73.0	73.4	76.0	74.9
Wholesale price (DPI-L)[a]	94.8	94.4	90.8	94.4	90.2	92.1	91.0	93.7	95.0	92.1	92.1	95.1	93.1	95.1	99.7	98.7
- Essential medicines	101.0	101.1	96.3	101.9	95.5	97.2	95.9	99.1	101.1	97.0	96.9	101.2	98.8	101.4	107.3	106.1
- Non-essential medicines	80.8	79.3	78.4	77.7	78.3	80.5	79.9	81.5	81.4	81.0	81.2	81.4	80.3	81.0	82.7	82.1
Wholesale price (DPI-F)[a]	95.8	94.6	93.2	95.2	92.4	94.7	93.2	94.5	96.5	93.4	95.3	98.1	96.7	96.0	102.1	101.3
- Essential medicines	101.4	100.8	100.1	100.2	98.2	98.4	97.7	99.1	101.1	99.8	100.2	104.4	102.9	102.6	109.1	108.9

- Non-essential medicines	80.3	79.5	77.8	81.2	77.4	84.6	82.0	79.3	83.5	77.3	80.9	80.7	80.2	80.6	83.6	82.2
**THCs (primary care)**																
Number of medicines	252	226	243	235	264	253	267	248	265	258	259	263	240	216	229	238
- Essential medicines	252	224	241	233	261	250	264	245	255	248	249	253	231	207	220	229
- Non-essential medicines	0	2	2	2	3	3	3	3	10	10	10	10	9	9	9	9
Sales (US$1,000)	51	35	43	39	50	46	44	54	54	44	44	47	63	45	67	64
- Essential medicines	51	35	43	39	50	46	44	54	52	43	42	46	59	43	64	60
- Non-essential medicines	0	0	0	0	0	0	0	0	2	1	2	1	3	2	4	3
Retail price (DPI-L)[c]	92.2	98.3	91.2	87.1	85.7	86.4	90.5	95.6	93.7	91.3	91.1	91.4	93.3	97.4	99.8	106.1
Retail price (DPI-F)[c]	94.3	89.3	93.5	83.8	86.2	87.5	88.1	94.6	95.7	90.0	90.5	88.2	93.2	93.1	101.3	102.1
Wholesale price (DPI-L)[c]	104.5	111.3	103.3	98.7	97.1	98.0	102.6	108.3	106.2	103.5	103.2	103.6	105.7	110.3	113.1	120.3
Wholesale price (DPI-F)[c]	106.7	100.8	105.5	94.7	97.3	98.6	99.6	106.7	107.9	101.7	102.2	99.8	105.3	105.1	114.4	115.3

a. Drug price index was calculated based on 426 unique medicines with records in each period from 2009-2016 (baseline: 100).

b. Drug price index was calculated based on 383 unique medicines with records in each period from 2009-2016 (baseline: 100).

c. Drug price index was calculated based on 117 unique medicines (essential medicines only) with records in each period from 2009-2016 (baseline: 100).

Note: DPI-F, Fisher Price Index; DPI-L, Laspeyres Price Index; DPI-P, Paasche Price Index; NEMP, National Essential Medicines Policy

Appendix 5. Number of medicines by western or Traditional Chinese medicines

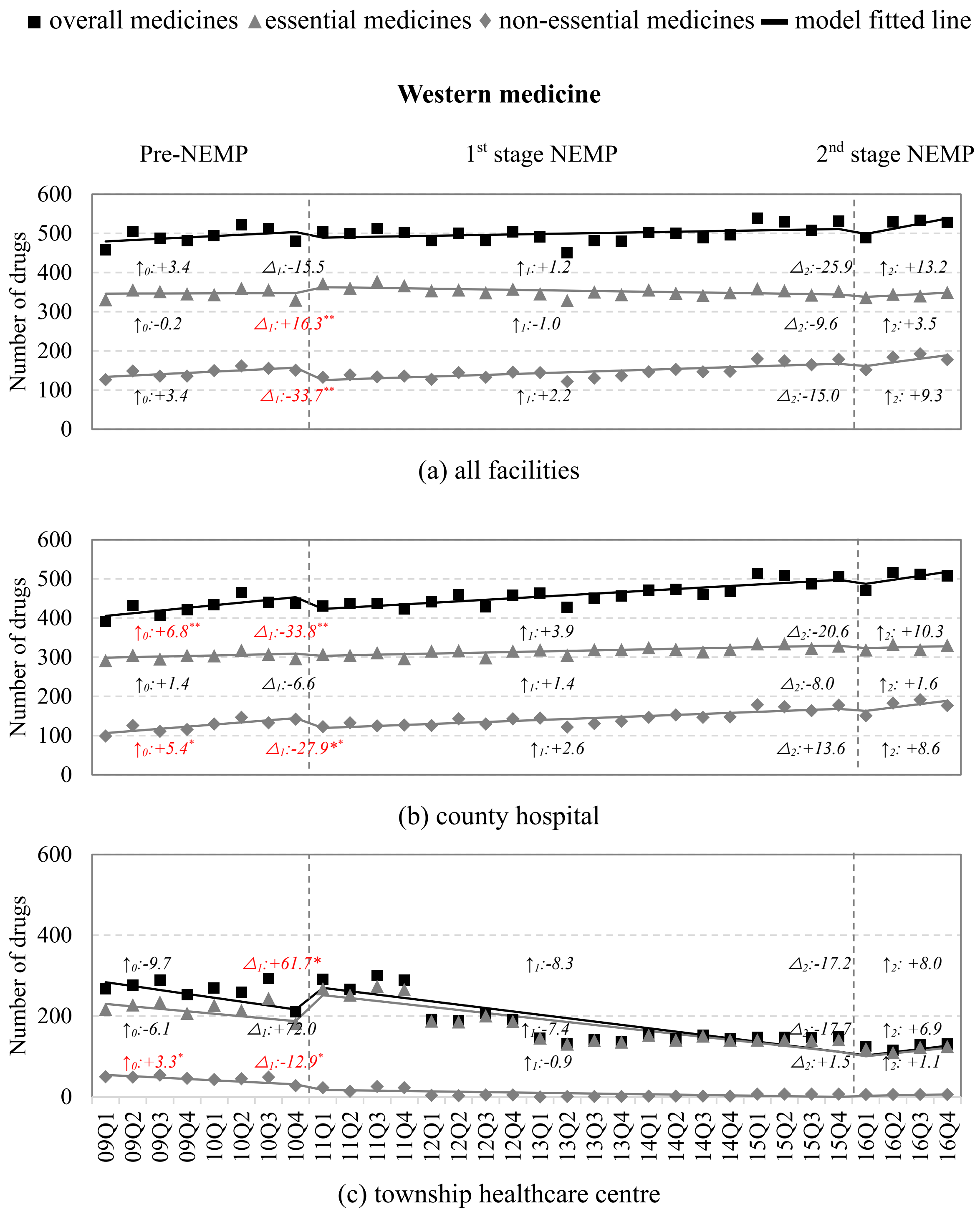

Traditional Chinese medicine

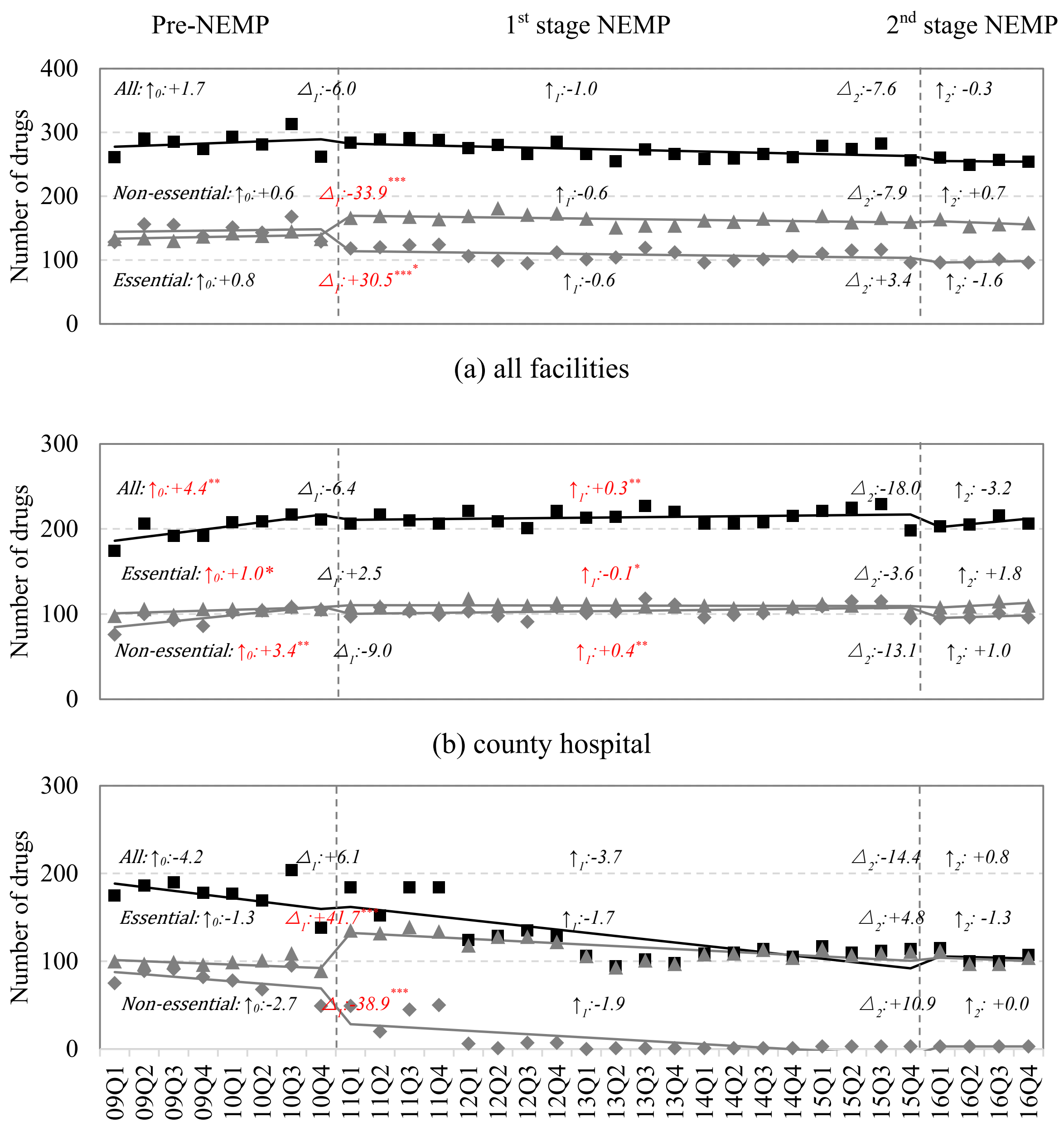

(c) township healthcare centre

$\uparrow_0$: sustained change per observation period before first-phase NEMP (β_1); $\triangle_1$: immediate change after first-phase NEMP (estimator β_3 in ITSA model); $\uparrow_1$: sustained change per observation period after first-phase NEMP ($\beta_1+\beta_3$); $\triangle_2$: immediate change after second-phase NEMP (β_4); $\uparrow_2$: sustained change per observation period after second-phase NEMP ($\beta_1+\beta_3+\beta_5$); ITSA, interrupted time-series analysis; NEMP, National Essential Medicines Policy; *, $P<0.05$; **, $P<0.01$; ***, $P<0.001$

Appendix 6. Number of medicines by ATC system for western medicines and TCM classification

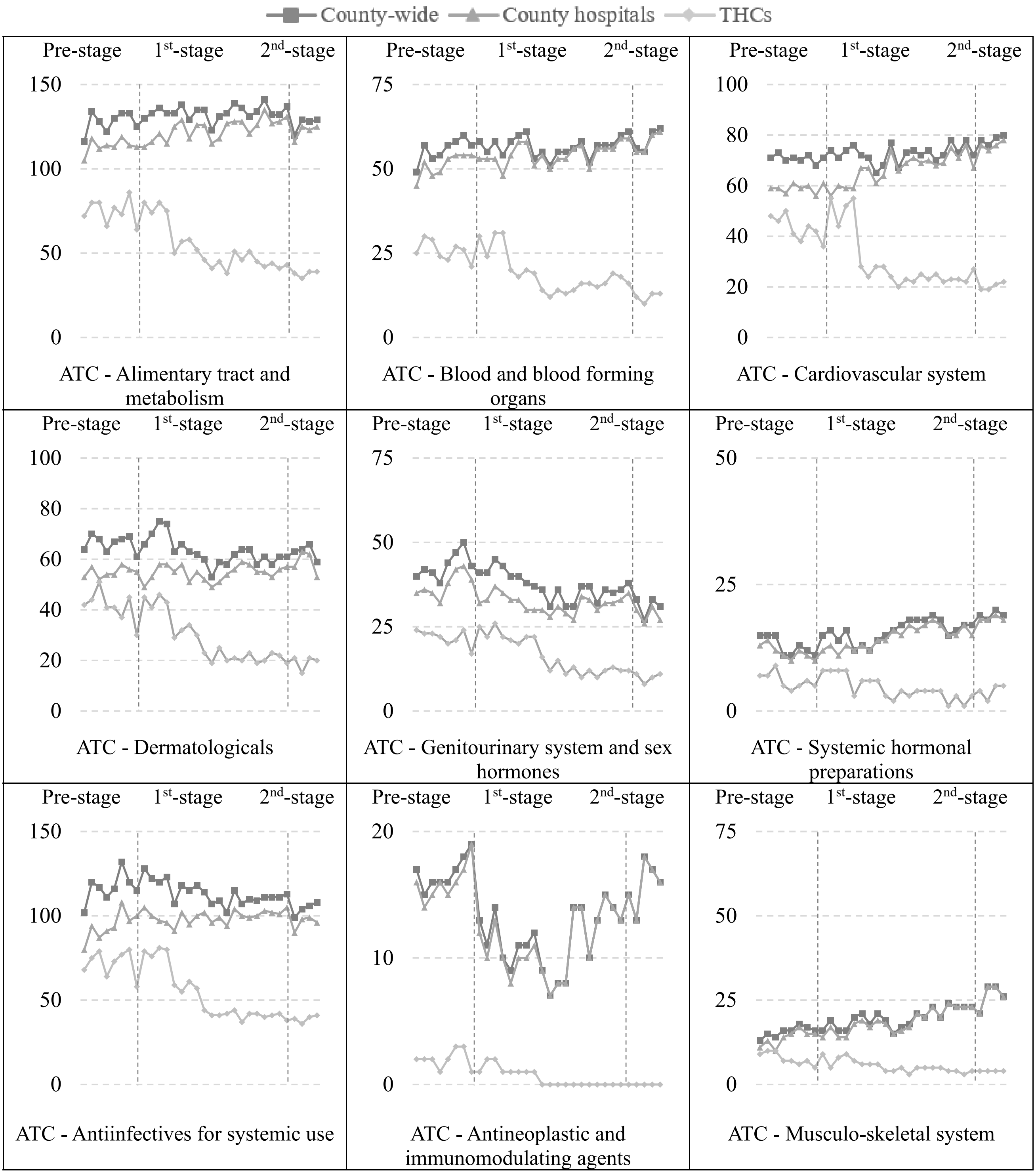

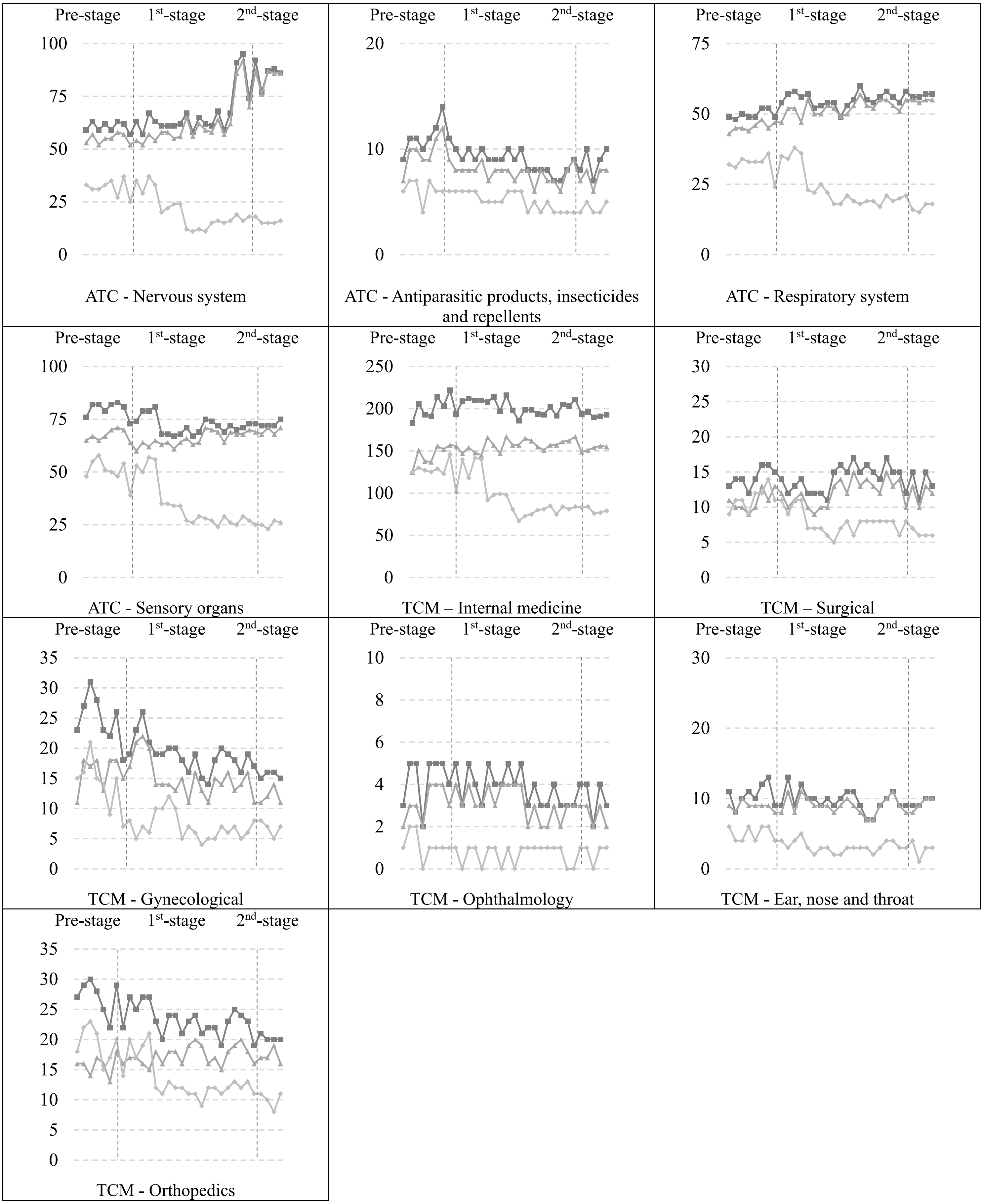
Pre-stage
1st-stage
2nd-stage
ATC - Nervous system
ATC - Antiparasitic products, insecticides and repellents
ATC - Respiratory system
ATC - Sensory organs
TCM – Internal medicine
TCM – Surgical
TCM - Gynecological
TCM - Ophthalmology
TCM - Ear, nose and throat
TCM - Orthopedics

Appendix 7. Interrupted time-series analysis on medicine number

Facility	Drug category	Baseline β_0	Time β_1 (*p-value*)	1st-stage NEMP β_2 (*p-value*)	Time after 1st-stage NEMP β_3 (*p-value*)	2nd-stage NEMP β_4 (*p-value*)	Time after 2nd-stage NEMP β_5 (*p-value*)
County-wide	Overall	757	4.9 (0.301)	-17.7 (0.464)	-4.9 (0.318)	-28.0 (0.459)	11.7 (0.373)
	- Essential drug	479	1.0 (0.669)	48.3 (<.001)	-2.5 (0.284)	-8.2 (0.648)	4.0 (0.521)
	- Non-essential drug	277	3.9 (0.170)	-66.2 (<.001)	-2.4 (0.417)	-19.6 (0.398)	7.7 (0.336)
	Western medicine	480	3.4 (0.293)	-15.5 (0.354)	-2.3 (0.501)	-25.9 (0.319)	12.1 (0.182)
	- Essential drug	346	0.2 (0.910)	16.3 (0.051)	-1.2 (0.472)	-9.6 (0.467)	4.5 (0.327)
	- Non-essential drug	134	3.4 (0.080)	-33.7 (0.002)	-1.2 (0.537)	-15.0 (0.324)	7.1 (0.181)
	TCM	277	1.7 (0.298)	-6.0 (0.467)	-2.7 (0.108)	-7.6 (0.600)	0.7 (0.888)
	- Essential drug	133	0.8 (0.450)	30.5 (<.001)	-1.4 (0.240)	3.4 (0.685)	-1.1 (0.712)
	- Non-essential drug	144	0.6 (0.715)	-33.9 (<.001)	-1.1 (0.486)	-7.9 (0.558)	1.3 (0.786)
	By ATC-classification:						
	- Alimentary tract and metabolism	125	0.9 (0.165)	1.2 (0.709)	-0.7 (0.245)	-13.9 (0.017)	2.3 (0.244)
	- Blood and blood forming organs	52	1.1 (0.020)	-3.4 (0.133)	-1.0 (0.032)	-4.7 (0.204)	2.3 (0.069)
	- Cardiovascular system	72	-0.2 (0.578)	1.2 (0.572)	0.4 (0.399)	1.8 (0.623)	0.9 (0.490)
	- Dermatological	67	-0.3 (0.714)	3.8 (0.322)	-0.3 (0.750)	6.0 (0.266)	-0.3 (0.893)
	- Genito urinary system and sex hormones	40	0.9 (0.137)	-4.5 (0.129)	-1.3 (0.042)	-2.8 (0.515)	0.5 (0.728)
	- Systemic hormonal preparations	15	-0.6 (0.043)	3.3 (0.029)	0.8 (0.019)	1.6 (0.420)	-0.2 (0.810)
	- Anti-infective for systemic use	111	1.7 (0.040)	-2.6 (0.529)	-2.4 (0.007)	-9.0 (0.211)	3.3 (0.189)
	- Antineoplastic and immunomodulating agents	16	0.2 (0.548)	-7.5 (<.001)	-0.1 (0.854)	-0.8 (0.789)	1.0 (0.333)
	- Musculo-skeletal system	14	0.5 (0.122)	-1.2 (0.457)	-0.2 (0.591)	-0.3 (0.900)	1.2 (0.217)
	- Nervous system	61	-0.4 (0.770)	-4.0 (0.562)	1.7 (0.241)	-7.5 (0.461)	2.6 (0.466)
	- Antiparasitic products, insecticides and repellents	10	0.4 (0.004)	-2.7 (<.001)	-0.5 (<.001)	0.7 (0.567)	0.3 (0.398)
	- Respiratory system	49	0.3 (0.523)	3.9 (0.059)	-0.2 (0.651)	-0.9 (0.773)	0.5 (0.649)
	- Sensory organs	80	-0.4 (0.584)	-2.6 (0.462)	0.2 (0.830)	-1.0 (0.842)	1.5 (0.391)
	By TCM-classification:						
	- Internal medicine	192	2.6 (0.030)	-1.5 (0.793)	-3.2 (0.010)	-3.1 (0.765)	0.0 (0.997)
	- Surgical	13	0.4 (0.128)	-3.0 (0.026)	-0.2 (0.342)	-1.6 (0.460)	-0.3 (0.677)
	- Gynecological	27	-0.8 (0.084)	-0.3 (0.911)	0.6 (0.249)	-1.1 (0.755)	0.3 (0.812)
	- Orthopedics	29	-0.7 (0.036)	1.4 (0.412)	0.5 (0.139)	-0.2 (0.938)	-0.1 (0.954)
	- Ear, nose and throat	10	0.2 (0.360)	-0.6 (0.591)	-0.3 (0.206)	-0.4 (0.831)	0.5 (0.444)
	- Ophthalmology	4	0.1 (0.243)	-0.3 (0.558)	-0.2 (0.095)	0.1 (0.913)	0.0 (0.987)
County hospitals	Overall	591	11.2 (<.001)	-40.1 (0.011)	-7.0 (0.024)	-37.8 (0.140)	9.0 (0.305)
	- Essential drug	400	2.4 (0.036)	-3.9 (0.493)	-1.1 (0.322)	-11.9 (0.244)	2.3 (0.517)
	- Non-essential drug	191	8.9 (<.001)	-36.9 (0.001)	-5.9 (0.008)	-26.4 (0.138)	6.7 (0.274)

	Western medicine	405	6.8 (0.004)	-33.8 (0.006)	-2.9 (0.204)	-20.6 (0.294)	6.4 (0.346)
	- Essential drug	299	1.4 (0.108)	-6.6 (0.146)	-0.1 (0.934)	-8.0 (0.324)	0.2 (0.933)
	- Non-essential drug	106	5.4 (0.002)	-27.9 (0.002)	-2.9 (0.094)	-13.6 (0.327)	6.0 (0.212)
	TCM	186	4.4 (0.001)	-6.4 (0.302)	-4.0 (0.003)	-18.0 (0.100)	2.9 (0.435)
	- Essential drug	101	1.0 (0.019)	2.5 (0.239)	-1.1 (0.015)	-3.6 (0.334)	1.9 (0.146)
	- Non-essential drug	85	3.4 (0.003)	-9.0 (0.108)	-3.1 (0.009)	-13.1 (0.155)	0.7 (0.835)
	By ATC-classification:						
	- Alimentary tract and metabolism	112	0.6 (0.367)	0.9 (0.792)	0.1 (0.870)	-13.7 (0.020)	1.7 (0.383)
	- Blood and blood forming organs	47	1.1 (0.008)	-3.4 (0.111)	-0.9 (0.041)	-4.7 (0.173)	2.0 (0.090)
	- Cardiovascular system	59	0.0 (0.942)	0.4 (0.832)	0.8 (0.060)	-2.0 (0.575)	0.6 (0.623)
	- Dermatological	54	0.4 (0.459)	-3.4 (0.231)	-0.2 (0.660)	5.2 (0.222)	-1.4 (0.354)
	- Genito urinary system and sex hormones	34	0.9 (0.055)	-8.3 (0.002)	-1.0 (0.056)	-2.5 (0.482)	-0.3 (0.815)
	- Systemic hormonal preparations	13	-0.4 (0.049)	1.7 (0.123)	0.7 (0.004)	1.6 (0.331)	-0.4 (0.523)
	- Anti-infective for systemic use	85	2.7 (<.001)	-6.7 (0.013)	-2.5 (<.001)	-9.4 (0.047)	1.4 (0.376)
	- Antineoplastic and immunomodulating agents	15	0.4 (0.354)	-8.3 (<.001)	-0.1 (0.750)	-0.7 (0.796)	0.9 (0.376)
	- Musculo-skeletal system	11	0.7 (0.031)	-2.4 (0.144)	-0.2 (0.455)	-0.7 (0.794)	1.0 (0.271)
	- Nervous system	55	0.0 (0.994)	-7.0 (0.249)	1.5 (0.231)	-3.3 (0.723)	2.0 (0.532)
	- Antiparasitic products, insecticides and repellents	9	0.3 (0.015)	-2.8 (<.001)	-0.4 (0.009)	-0.6 (0.605)	0.3 (0.411)
	- Respiratory system	44	0.4 (0.140)	2.9 (0.044)	-0.2 (0.524)	0.3 (0.903)	-0.1 (0.862)
	- Sensory organs	66	0.3 (0.357)	-7.3 (<.001)	0.1 (0.812)	-1.8 (0.556)	0.2 (0.860)
	By TCM-classification:						
	- Internal medicine	135	3.4 (<.001)	-6.8 (0.136)	-3.0 (0.002)	-13.1 (0.103)	1.8 (0.507)
	- Surgical	10	0.4 (0.135)	-1.7 (0.173)	-0.2 (0.412)	-2.0 (0.329)	0.0 (0.946)
	- Gynecological	15	0.3 (0.437)	1.0 (0.646)	-0.6 (0.167)	-0.5 (0.886)	0.4 (0.730)
	- Orthopedics	15	0.1 (0.737)	0.6 (0.603)	0.0 (0.985)	-0.8 (0.684)	-0.1 (0.894)
	- Ear, nose and throat	9	-0.1 (0.762)	0.7 (0.477)	0.0 (0.914)	-1.1 (0.449)	0.7 (0.164)
	- Ophthalmology	2	0.2 (0.043)	-0.1 (0.876)	-0.3 (0.013)	0.4 (0.616)	-0.1 (0.658)
THCs	Overall	474	-14.7 (0.150)	75.2 (0.127)	2.5 (0.818)	-3.7 (0.954)	20.5 (0.392)
	- Essential drug	331	-7.6 (0.265)	115.3 (<.001)	-1.5 (0.843)	-13.5 (0.730)	14.7 (0.341)
	- Non-essential drug	142	-5.6 (0.092)	-55.7 (0.002)	2.8 (0.402)	14.1 (0.581)	3.6 (0.684)
	Western medicine	284	-9.7 (0.141)	61.7 (0.044)	1.3 (0.853)	-17.2 (0.646)	16.3 (0.269)
	- Essential drug	230	-6.1 (0.240)	72.0 (0.005)	-1.3 (0.824)	-17.7 (0.557)	14.3 (0.229)
	- Non-essential drug	54	-3.3 (0.017)	-12.9 (0.048)	2.5 (0.103)	1.5 (0.853)	2.0 (0.529)
	TCM	189	-4.2 (0.239)	6.1 (0.733)	0.5 (0.899)	14.4 (0.583)	2.9 (0.752)
	- Essential drug	101	-1.3 (0.467)	41.7 (<.001)	-0.4 (0.843)	4.8 (0.659)	0.4 (0.927)
	- Non-essential drug	88	-2.7 (0.187)	-38.9 (<.001)	0.8 (0.706)	10.9 (0.519)	1.9 (0.744)
	By ATC-classification:						
	- Alimentary tract and metabolism	77	-0.8 (0.545)	2.5 (0.722)	-1.1 (0.476)	-0.9 (0.930)	3.0 (0.414)
	- Blood and blood forming organs	27	-1.0 (0.222)	6.8 (0.071)	0.2 (0.784)	-3.7 (0.430)	1.6 (0.364)

	- Cardiovascular system	49	-2.5 (0.094)	15.2 (0.028)	1.0 (0.537)	-6.6 (0.429)	4.0 (0.226)
	- Dermatological	46	-1.6 (0.075)	7.3 (0.114)	0.2 (0.810)	3.5 (0.606)	1.8 (0.455)
	- Genito urinary system and sex hormones	24	-0.7 (0.094)	6.1 (0.010)	-0.1 (0.884)	0.1 (0.963)	1.2 (0.298)
	- Systemic hormonal preparations	7	-0.4 (0.089)	3.0 (0.012)	0.1 (0.742)	1.0 (0.597)	0.9 (0.163)
	- Anti-infective for systemic use	74	-1.4 (0.360)	14.2 (0.060)	-1.0 (0.542)	2.7 (0.781)	4.2 (0.248)
	- Antineoplastic and immunomodulating agents	2	0.0 (0.954)	-0.4 (0.371)	-0.1 (0.344)	0.3 (0.677)	0.1 (0.592)
	- Musculo-skeletal system	10	-0.7 (<.001)	2.3 (0.010)	0.4 (0.017)	0.8 (0.561)	0.2 (0.662)
	- Nervous system	34	-1.2 (0.259)	5.5 (0.286)	0.2 (0.843)	-1.0 (0.877)	2.2 (0.380)
	- Antiparasitic products, insecticides and repellents	6	-0.1 (0.445)	0.3 (0.503)	0.0 (0.644)	0.6 (0.496)	0.1 (0.729)
	- Respiratory system	34	-1.2 (0.170)	8.4 (0.041)	0.3 (0.753)	-4.5 (0.367)	2.5 (0.220)
	- Sensory organs	54	-1.6 (0.176)	9.0 (0.120)	0.0 (0.980)	1.4 (0.848)	2.8 (0.317)
	By TCM-classification:						
	- Internal medicine	132	-3.0 (0.291)	17.1 (0.231)	-0.1 (0.973)	9.7 (0.618)	3.6 (0.612)
	- Surgical	10	0.3 (0.205)	-2.9 (0.038)	-0.5 (0.101)	0.0 (0.997)	-0.1 (0.892)
	- Gynecological	18	-1.2 (0.006)	-1.7 (0.391)	1.1 (0.014)	2.0 (0.537)	-0.3 (0.755)
	- Orthopedics	21	-0.8 (0.098)	1.9 (0.443)	0.4 (0.397)	0.4 (0.912)	0.4 (0.774)
	- Ear, nose and throat	5	0.0 (0.841)	-1.8 (0.016)	0.0 (0.752)	-0.1 (0.965)	0.0 (0.907)
	- Ophthalmology	1	-0.1 (0.207)	-0.1 (0.696)	0.1 (0.206)	-0.3 (0.683)	0.1 (0.621)

Note: Segmented linear regression model was built with two interruption points: $Y_t = \beta_0 + \beta_1 T + \beta_2 X_{1\_t} + \beta_3 T X_{1\_t} + \beta_4 X_{2\_t} + \beta_5 T X_{2\_t} + \varepsilon_t$, Coefficient β0 estimates the baseline level of outcome; β1 estimates the time trend of outcome in the pre-policy period; β2 and β4 estimate the immediate changes in level after the first- and second-stage policy; and β3 and β5¬ estimate the sustained change in trend after the first- and second-stage policy; ATC, Anatomical Therapeutic Chemical; TCM, Traditional Chinese Medicine; NEMP, National Essential Medicines Policy; *, P<0.05; **, P<0.01; ***, P<0.001

Appendix 8. Sales of medicines by western or Traditional Chinese medicines

■ overall medicines ▲essential medicines ♦ non-essential medicines ▬ model fitted line

Western medicine

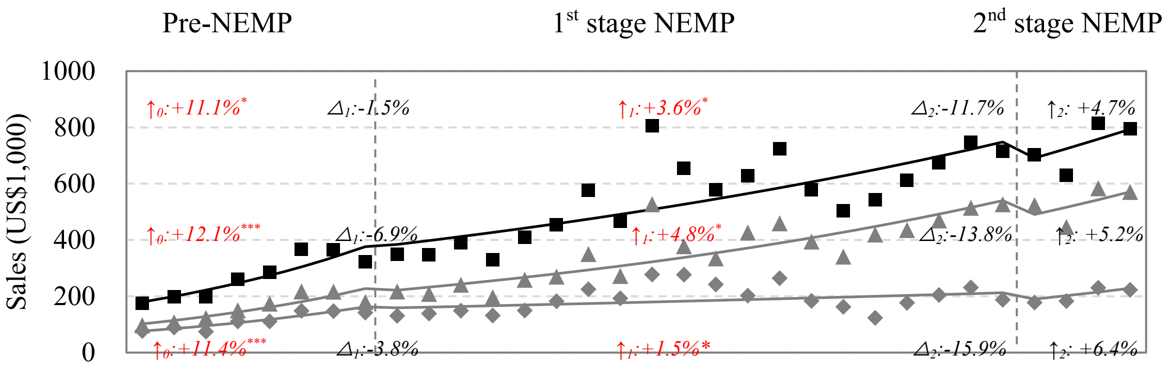

(a) all facilities

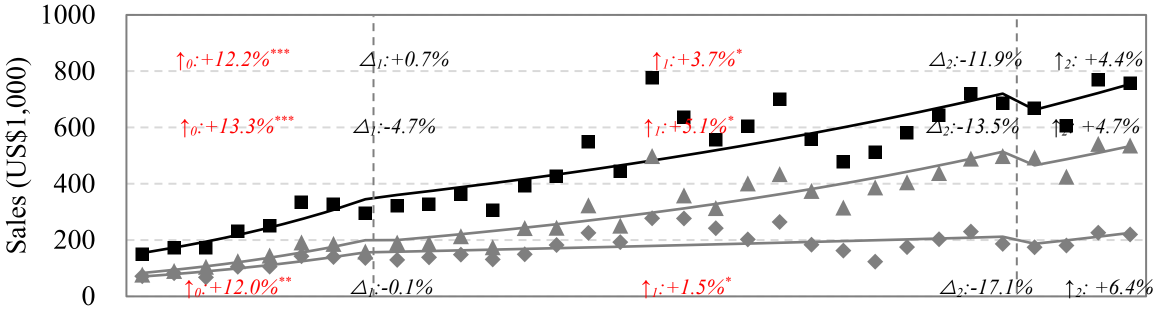

(b) county hospital

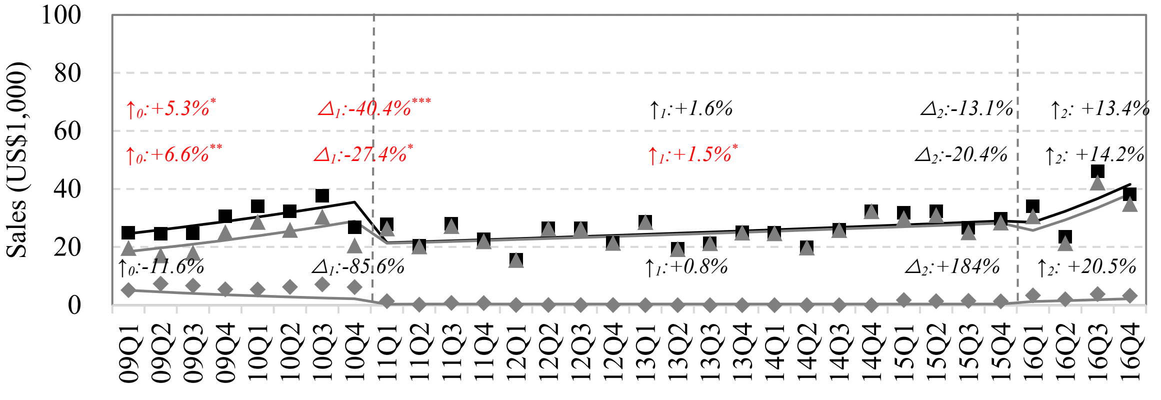

(c) township healthcare centre

Traditional Chinese medicine

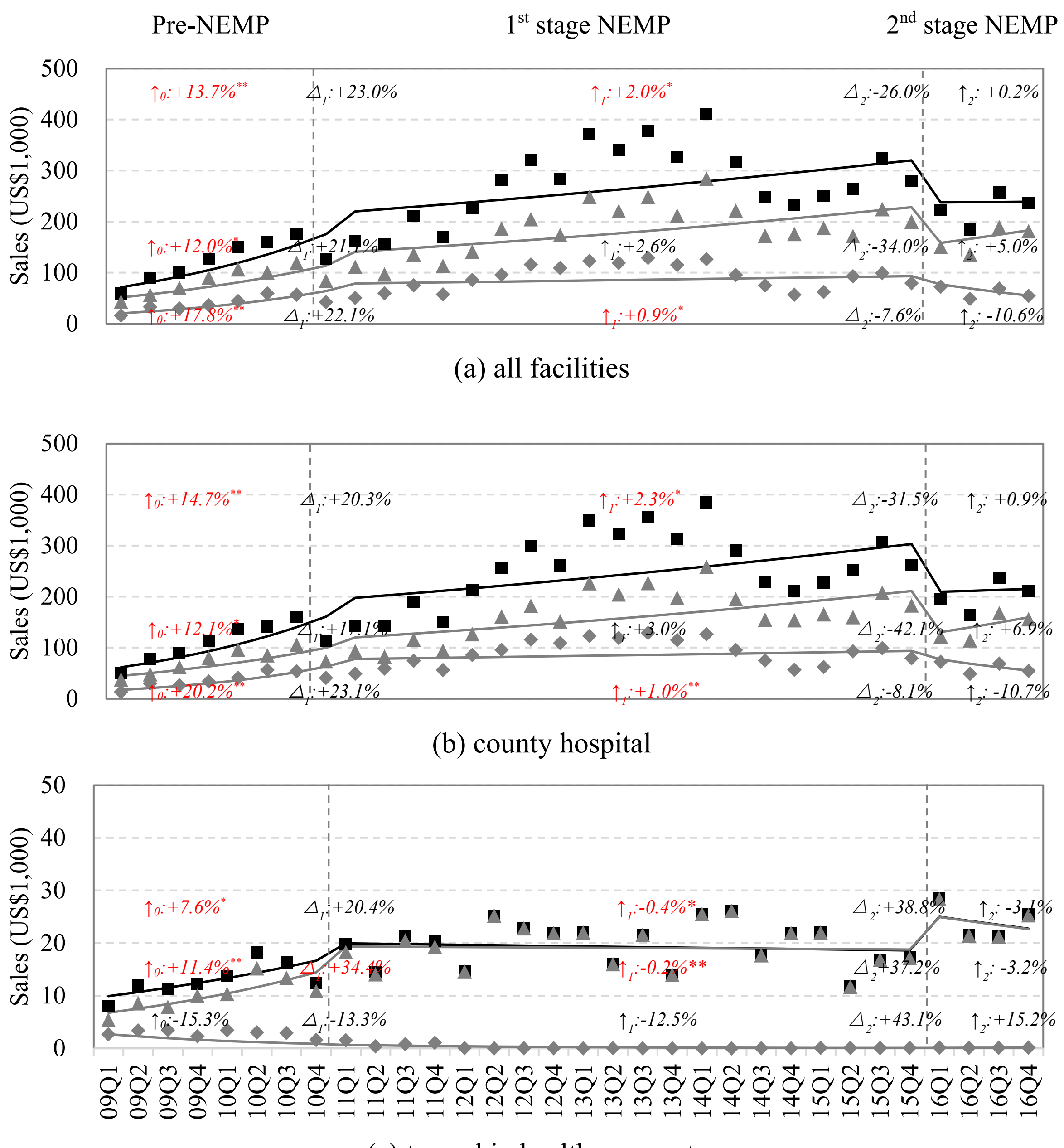

(c) township healthcare centre

↑0: sustained change per observation period before first-phase NEMP (β_1); △1: immediate change after first-phase NEMP (estimator β_3 in ITSA model); ↑1: sustained change per observation period after first-phase NEMP ($\beta_1+\beta_3$); △2: immediate change after second-phase NEMP (β_4); ↑2: sustained change per observation period after second-phase NEMP ($\beta_1+\beta_3+\beta_5$); ITSA, interrupted time-series analysis; NEMP, National Essential Medicines Policy; *, P<0.05; **, P<0.01; ***, P<0.001

Appendix 9. Sales of medicines by ATC system for western medicines and TCM classification (US$1,000)

(a) county hospitals

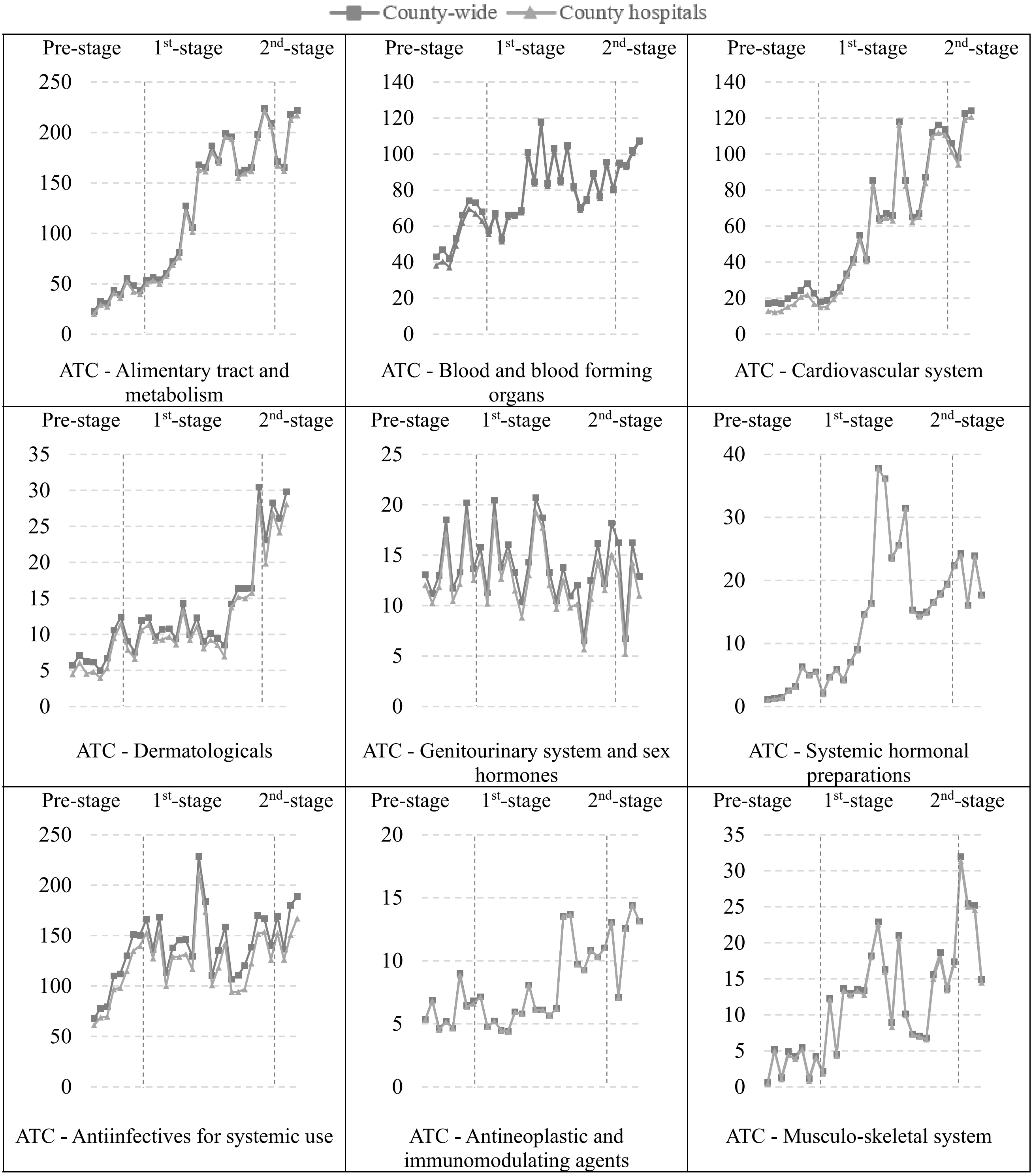

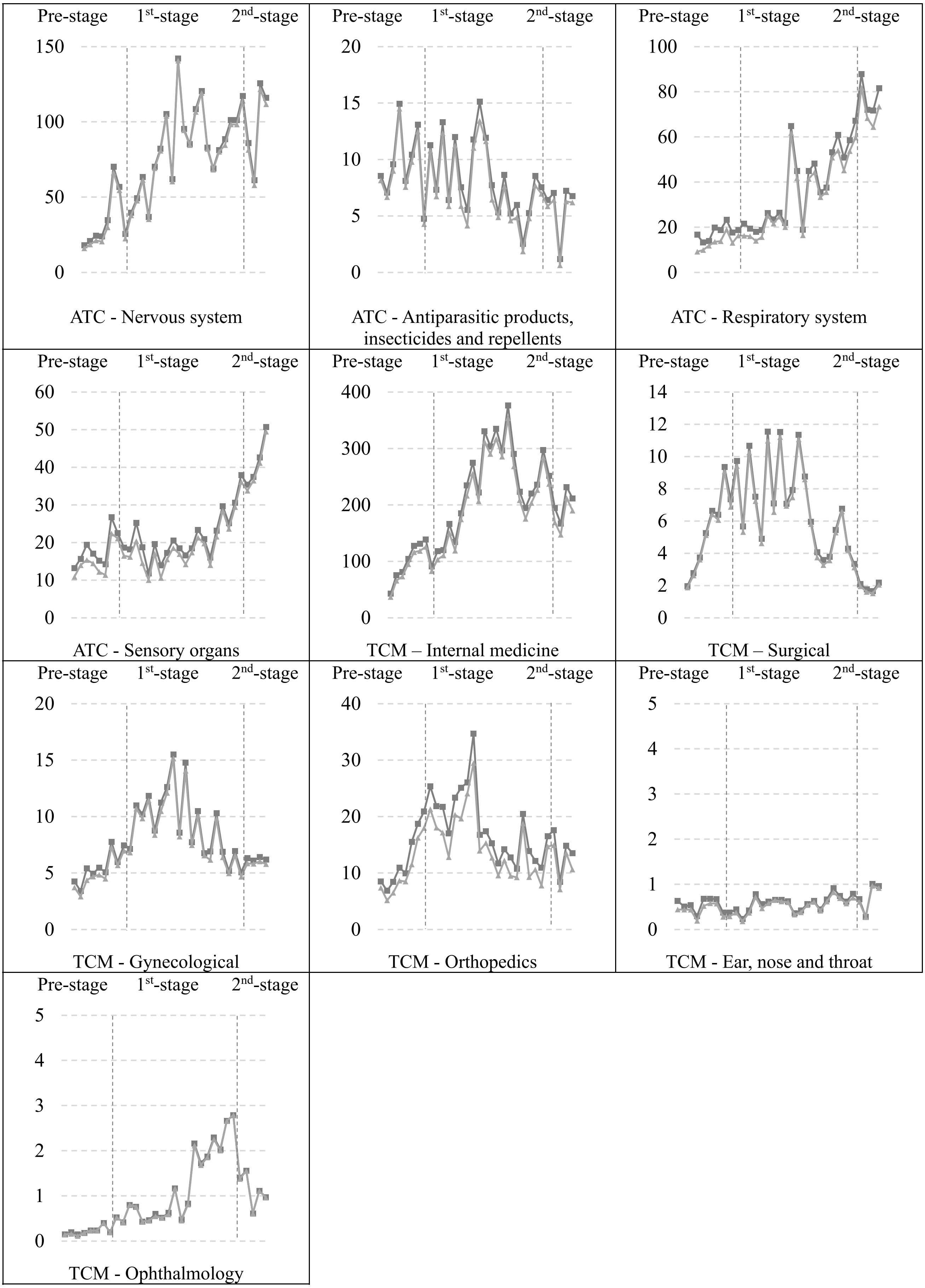

Pre-stage
1st-stage
2nd-stage
ATC - Nervous system
ATC - Antiparasitic products, insecticides and repellents
ATC - Respiratory system
ATC - Sensory organs
TCM – Internal medicine
TCM – Surgical
TCM - Gynecological
TCM - Orthopedics
TCM - Ear, nose and throat
TCM - Ophthalmology

(b) THCs

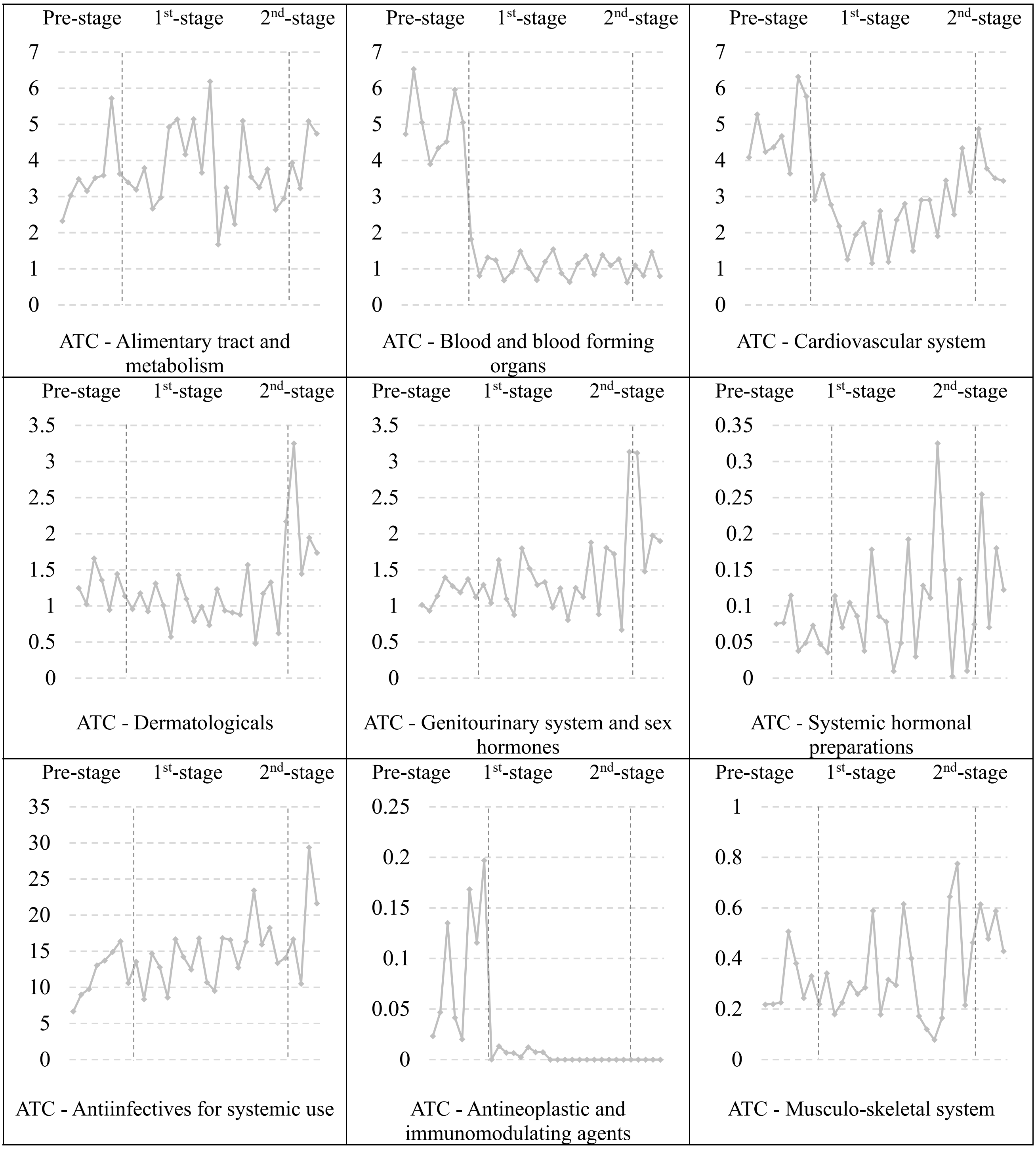
Pre-stage 1st-stage 2nd-stage
7 6 5 4 3 2 1 0
ATC - Alimentary tract and metabolism
Pre-stage 1st-stage 2nd-stage
7 6 5 4 3 2 1 0
ATC - Blood and blood forming organs
Pre-stage 1st-stage 2nd-stage
7 6 5 4 3 2 1 0
ATC - Cardiovascular system
Pre-stage 1st-stage 2nd-stage
3.5 3 2.5 2 1.5 1 0.5 0
ATC - Dermatologicals
Pre-stage 1st-stage 2nd-stage
3.5 3 2.5 2 1.5 1 0.5 0
ATC - Genitourinary system and sex hormones
Pre-stage 1st-stage 2nd-stage
0.35 0.3 0.25 0.2 0.15 0.1 0.05 0
ATC - Systemic hormonal preparations
Pre-stage 1st-stage 2nd-stage
35 30 25 20 15 10 5 0
ATC - Antiinfectives for systemic use
Pre-stage 1st-stage 2nd-stage
0.25 0.2 0.15 0.1 0.05 0
ATC - Antineoplastic and immunomodulating agents
Pre-stage 1st-stage 2nd-stage
1 0.8 0.6 0.4 0.2 0
ATC - Musculo-skeletal system

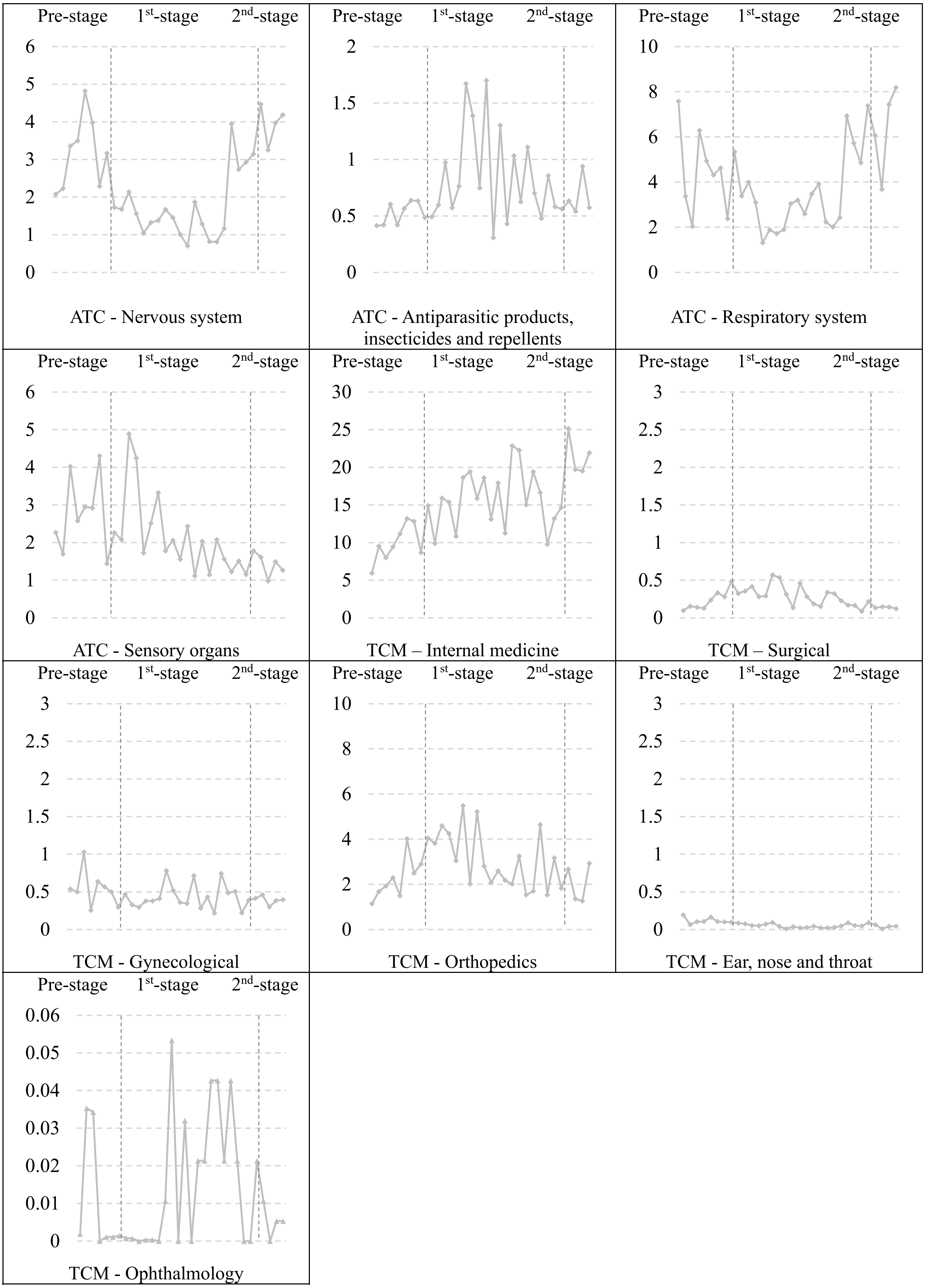
Pre-stage
1st-stage
2nd-stage
ATC - Nervous system
ATC - Antiparasitic products, insecticides and repellents
ATC - Respiratory system
ATC - Sensory organs
TCM – Internal medicine
TCM – Surgical
TCM - Gynecological
TCM - Orthopedics
TCM - Ear, nose and throat
TCM - Ophthalmology

Appendix 10. Interrupted time-series analysis on medicine sales after log transformation

Facility	Drug category	Baseline US$1,000, $\exp(\beta_0)$	Time, $\exp(\beta_1)$-1 (*p-value*)	1st-stage NEMP, $\exp(\beta_2)$-1 (*p-value*)	Time after 1st-stage NEMP, $\exp(\beta_3)$-1 (*p-value*)	2nd-stage NEMP, $\exp(\beta_4)$-1 (*p-value*)	Time after 2nd-stage NEMP, $\exp(\beta_4)$-1 (*p-value*)
County-wide	Overall	253.7	11.7% (0.002)	6.7% (0.683)	-7.7% (0.036)	-15.1% (0.442)	11.3% (0.567)
	- Essential drug	155.9	11.6% (0.001)	3.9% (0.802)	-6.8% (0.036)	-18.3% (0.363)	10.2% (0.586)
	- Non-essential drug	97.7	12.9% (0.009)	2.0% (0.917)	-10.2% (0.035)	-13.3% (0.559)	13.4% (0.596)
	Western medicine	179.7	11.1% (0.001)	-1.5% (0.917)	-6.8% (0.031)	-11.7% (0.551)	11.4% (0.561)
	- Essential drug	102.1	12.1% (<.001)	-6.9% (0.577)	-6.5% (0.013)	-13.8% (0.475)	7.8% (0.670)
	- Non-essential drug	76.3	11.4% (0.012)	-3.8% (0.840)	-8.9% (0.047)	-15.9% (0.486)	19.8% (0.455)
	TCM	71.2	13.7% (0.007)	23.0% (0.310)	-10.3% (0.040)	-26.0% (0.244)	11.6% (0.631)
	- Essential drug	51.3	12.0% (0.013)	21.1% (0.347)	-8.4% (0.074)	-34.0% (0.123)	18.0% (0.458)
	- Non-essential drug	20.3	17.8% (0.006)	22.1% (0.432)	-14.4% (0.018)	-7.6% (0.807)	-4.3% (0.869)
	By ATC-classification:						
	- Alimentary tract and metabolism	25.9	10.6% (0.018)	11.6% (0.552)	-2.9% (0.530)	-29.1% (0.150)	-1.0% (0.913)
	- Blood and blood forming organs	42.2	9.0% (0.007)	-13.2% (0.352)	-6.8% (0.034)	1.7% (0.938)	1.4% (0.860)
	- Cardiovascular system	16.6	7.0% (0.113)	-22.1% (0.231)	2.6% (0.564)	-20.9% (0.411)	-5.5% (0.586)
	- Dermatological	5.3	9.9% (0.056)	-24.6% (0.235)	-5.1% (0.322)	-8.5% (0.781)	11.3% (0.360)
	- Genito urinary system and sex hormones	12.4	3.8% (0.357)	-6.1% (0.763)	-4.6% (0.256)	-5.7% (0.867)	3.4% (0.785)
	- Systemic hormonal preparations	1.2	34.9% (0.005)	-61.1% (0.019)	-17.5% (0.115)	15.8% (0.746)	-22.8% (0.211)
	- Anti-infective for systemic use	68.4	13.1% (<.001)	-8.4% (0.540)	-11.9% (<.001)	4.4% (0.851)	6.4% (0.437)
	- Antineoplastic and immunomodulating agents	5.3	3.5% (0.361)	-34.3% (0.038)	1.7% (0.676)	-44.6% (0.063)	16.3% (0.163)
	- Musculo-skeletal system	2.0	8.0% (0.363)	142.5% (0.048)	-4.7% (0.574)	181.4% (0.173)	-22.8% (0.324)
	- Nervous system	19.6	13.3% (0.025)	14.5% (0.621)	-8.3% (0.124)	-44.0% (0.178)	11.7% (0.453)
	- Antiparasitic products, insecticides and repellents	9.4	-0.7% (0.916)	16.1% (0.641)	-2.3% (0.713)	-56.1% (0.148)	28.0% (0.211)
	- Respiratory system	14.7	5.1% (0.150)	-17.9% (0.258)	1.7% (0.623)	28.3% (0.406)	-8.7% (0.383)
	- Sensory organs	14.0	6.5% (0.089)	-30.0% (0.061)	-3.3% (0.368)	6.1% (0.833)	11.6% (0.268)
	By TCM-classification:	3.3	22.6% (0.009)	26.5% (0.524)	-16.6% (0.026)	8.7% (0.873)	-3.1% (0.865)
	- Internal medicine	55.9	13.8% (0.019)	21.9% (0.402)	-9.6% (0.097)	-29.3% (0.248)	-2.1% (0.854)
	- Surgical	2.4	22.3% (<.001)	5.0% (0.848)	-21.7% (<.001)	-54.7% (0.054)	4.1% (0.769)
	- Gynecological	4.0	8.0% (0.067)	69.7% (0.017)	-9.7% (0.021)	-7.3% (0.822)	1.7% (0.884)
	- Orthopedics	6.8	16.7% (<.001)	30.9% (0.186)	-17.5% (<.001)	14.6% (0.672)	1.6% (0.889)
	- Ear, nose and throat	0.5	-0.5% (0.924)	-23.7% (0.283)	3.6% (0.478)	-52.2% (0.086)	23.8% (0.148)
	- Ophthalmology	0.2	8.9% (0.115)	23.6% (0.439)	1.1% (0.842)	-54.0% (0.100)	-14.2% (0.338)
County hospitals	Overall	218.3	12.7% (0.002)	7.3% (0.673)	-8.4% (0.031)	-16.6% (0.405)	10.8% (0.606)
	- Essential drug	129.5	12.5% (0.001)	4.0% (0.810)	-7.2% (0.038)	-20.0% (0.340)	9.3% (0.645)
	- Non-essential drug	89.1	13.7% (0.006)	5.2% (0.797)	-10.8% (0.027)	-14.2% (0.538)	13.2% (0.604)
	Western medicine	154.5	12.2% (<.001)	0.7% (0.963)	-7.5% (0.024)	-11.9% (0.556)	10.0% (0.629)

	- Essential drug	83.3	13.3% (<.001)	-4.7% (0.729)	-7.2% (0.011)	-13.5% (0.510)	5.6% (0.775)
	- Non-essential drug	70.7	12.0% (0.009)	-0.1% (0.996)	-9.3% (0.039)	-17.1% (0.457)	19.7% (0.459)
	TCM	61.6	14.7% (0.007)	20.3% (0.383)	-10.8% (0.041)	-31.5% (0.161)	14.4% (0.575)
	- Essential drug	44.7	12.1% (0.018)	17.1% (0.462)	-8.1% (0.106)	-42.1% (0.055)	23.7% (0.375)
	- Non-essential drug	17.3	20.2% (0.003)	23.1% (0.428)	-16.0% (0.010)	-8.1% (0.800)	-4.6% (0.863)
	By ATC-classification:						
	- Alimentary tract and metabolism	23.4	10.9% (0.019)	13.8% (0.504)	-2.8% (0.546)	-30.2% (0.151)	-1.3% (0.887)
	- Blood and blood forming organs	37.3	10.0% (0.004)	-9.3% (0.533)	-7.6% (0.023)	1.6% (0.944)	1.4% (0.861)
	- Cardiovascular system	12.5	7.7% (0.127)	-13.3% (0.531)	2.6% (0.626)	-21.6% (0.425)	-6.2% (0.572)
	- Dermatological	4.0	12.4% (0.034)	-26.1% (0.248)	-6.9% (0.222)	-16.8% (0.603)	14.2% (0.307)
	- Genito urinary system and sex hormones	11.3	3.8% (0.403)	-4.9% (0.823)	-4.8% (0.275)	-17.7% (0.611)	6.9% (0.613)
	- Systemic hormonal preparations	1.1	36.5% (0.005)	-62.9% (0.016)	-18.3% (0.103)	14.7% (0.767)	-22.9% (0.217)
	- Anti-infective for systemic use	60.6	13.2% (<.001)	-4.8% (0.765)	-12.2% (<.001)	10.6% (0.691)	5.1% (0.572)
	- Antineoplastic and immunomodulating agents	5.2	3.3% (0.392)	-32.9% (0.048)	1.9% (0.632)	-44.5% (0.063)	16.2% (0.164)
	- Musculo-skeletal system	1.7	8.0% (0.382)	172.2% (0.033)	-4.7% (0.593)	183.9% (0.192)	-23.0% (0.345)
	- Nervous system	17.1	14.0% (0.025)	22.6% (0.479)	-8.8% (0.119)	-46.2% (0.169)	12.4% (0.450)
	- Antiparasitic products, insecticides and repellents	9.0	-0.9% (0.900)	11.1% (0.778)	-2.2% (0.767)	-66.3% (0.107)	36.4% (0.183)
	- Respiratory system	9.8	8.5% (0.030)	-16.9% (0.322)	-1.0% (0.787)	29.0% (0.425)	-10.5% (0.320)
	- Sensory organs	11.5	7.2% (0.088)	-33.5% (0.051)	-3.2% (0.435)	4.4% (0.888)	11.6% (0.309)
	By TCM-classification:						
	- Internal medicine	48.5	14.8% (0.017)	18.5% (0.488)	-10.1% (0.094)	-35.1% (0.166)	-1.6% (0.898)
	- Surgical	2.3	22.2% (<.001)	5.4% (0.843)	-21.6% (<.001)	-55.9% (0.058)	4.2% (0.775)
	- Gynecological	3.4	9.7% (0.036)	73.4% (0.017)	-11.2% (0.011)	-6.9% (0.836)	1.8% (0.881)
	- Orthopedics	5.3	17.8% (<.001)	30.9% (0.230)	-18.4% (<.001)	24.2% (0.554)	0.2% (0.989)
	- Ear, nose and throat	0.4	0.1% (0.982)	-18.5% (0.464)	3.6% (0.522)	-54.0% (0.102)	24.3% (0.181)
	- Ophthalmology	0.1	11.1% (0.069)	21.4% (0.502)	-0.9% (0.871)	-51.7% (0.138)	-14.9% (0.334)
THCs	Overall	34.7	6.0% (0.002)	-22.2% (0.008)	-4.8% (0.009)	2.5% (0.879)	23.7% (0.082)
	- Essential drug	25.4	7.8% (<.001)	-7.2% (0.459)	-6.4% (0.002)	-0.9% (0.959)	23.8% (0.106)
	- Non-essential drug	6.9	-13.1% (0.597)	-72.3% (0.197)	11.3% (0.739)	168.1% (0.395)	92.0% (0.727)
	Western medicine	24.6	5.3% (0.028)	-40.4% (<.001)	-3.6% (0.122)	-13.1% (0.496)	42.7% (0.027)
	- Essential drug	18.5	6.6% (0.009)	-27.4% (0.010)	-4.8% (0.044)	-20.4% (0.281)	45.3% (0.027)
	- Non-essential drug	5.1	-11.6% (0.611)	-85.6% (0.050)	14.0% (0.649)	183.9% (0.362)	77.4% (0.751)
	TCM	9.9	7.6% (0.019)	20.4% (0.233)	-7.4% (0.017)	38.8% (0.219)	-4.9% (0.813)
	- Essential drug	6.8	11.4% (0.002)	34.4% (0.075)	-10.4% (0.002)	37.2% (0.252)	-6.2% (0.766)
	- Non-essential drug	2.7	-15.3% (0.339)	-13.3% (0.822)	3.2% (0.878)	43.1% (0.632)	100.6% (0.684)
	By ATC-classification:						
	- Alimentary tract and metabolism	2.7	8.0% (0.057)	-15.9% (0.389)	-8.1% (0.041)	-4.8% (0.887)	13.2% (0.307)
	- Blood and blood forming organs	5.0	0.4% (0.924)	-77.9% (<.001)	-0.9% (0.811)	-6.0% (0.854)	3.3% (0.785)
	- Cardiovascular system	4.2	3.5% (0.486)	-62.8% (<.001)	-1.9% (0.711)	87.9% (0.140)	-12.0% (0.380)

	- Dermatological	1.3	-1.8% (0.601)	-15.3% (0.361)	2.2% (0.546)	235.0% (<.001)	-19.3% (0.074)
	- Genito urinary system and sex hormones	1.0	3.7% (0.326)	-13.4% (0.442)	-2.7% (0.462)	133.5% (0.017)	-16.9% (0.126)
	- Systemic hormonal preparations	0.1	-8.2% (0.376)	94.4% (0.185)	6.8% (0.502)	182.7% (0.236)	-7.8% (0.788)
	- Anti-infective for systemic use	8.3	10.1% (0.002)	-33.3% (0.009)	-7.1% (0.017)	-44.0% (0.035)	25.4% (0.021)
	- Antineoplastic and immunomodulating agents	0.0	20.7% (<.001)	-88.6% (<.001)	-20.1% (<.001)	16.7% (0.728)	3.0% (0.847)
	- Musculo-skeletal system	0.3	0.9% (0.918)	-4.3% (0.925)	-0.2% (0.982)	88.4% (0.359)	-7.1% (0.761)
	- Nervous system	2.5	3.1% (0.696)	-58.5% (0.028)	-1.0% (0.905)	64.6% (0.328)	2.8% (0.882)
	- Antiparasitic products, insecticides and repellents	0.5	3.6% (0.463)	47.1% (0.119)	-4.4% (0.352)	-21.5% (0.579)	8.9% (0.577)
	- Respiratory system	5.9	-11.8% (0.148)	3.6% (0.933)	16.2% (0.109)	-12.8% (0.819)	17.8% (0.447)
	- Sensory organs	2.5	1.4% (0.755)	11.6% (0.619)	-5.6% (0.199)	13.6% (0.744)	0.8% (0.953)
	By TCM-classification:						
	- Internal medicine	7.5	7.2% (0.057)	19.9% (0.323)	-6.4% (0.078)	48.5% (0.201)	-4.3% (0.678)
	- Surgical	0.1	21.4% (<.001)	8.2% (0.756)	-21.5% (<.001)	-8.0% (0.845)	1.9% (0.898)
	- Gynecological	0.6	-5.2% (0.231)	-2.9% (0.894)	5.5% (0.235)	-5.1% (0.895)	-1.2% (0.930)
	- Orthopedics	1.4	12.4% (0.002)	33.9% (0.103)	-14.4% (<.001)	-9.5% (0.756)	3.6% (0.760)
	- Ear, nose and throat	0.1	-5.0% (0.545)	-39.0% (0.262)	4.1% (0.657)	-15.0% (0.801)	2.8% (0.901)
	- Ophthalmology	0.0	-12.1% (0.223)	27.6% (0.652)	19.4% (0.111)	-48.0% (0.447)	-9.7% (0.730)

Note: Segmented linear regression model was built with two interruption points: $Y_t = \beta_0 + \beta_1 T + \beta_2 X_{1\_t} + \beta_3 T X_{1\_t} + \beta_4 X_{2\_t} + \beta_5 T X_{2\_t} + \varepsilon_t$, Coefficient β0 estimates the baseline level of outcome; β1 estimates the time trend of outcome in the pre-policy period; β2 and β4 estimate the immediate changes in level after the first- and second-stage policy; and β3 and β5¬ estimate the sustained change in trend after the first- and second-stage policy; ATC, Anatomical Therapeutic Chemical; TCM, Traditional Chinese Medicine; NEMP, National Essential Medicines Policy; *, P<0.05; **, P<0.01; ***, P<0.001

Appendix 11. Retail prices of medicines by western or Traditional Chinese medicines

■ overall (DPI-F) ▲ essential medicines (DPI-F) ♦ non-essential medicines (DPI-F)

□ overall (DPI-L) △ essential medicines (DPI-L) ◊ non-essential medicines (DPI-L)

▬ model fitted line

Western medicine

Pre-NEMP 1st stage NEMP 2nd stage NEMP

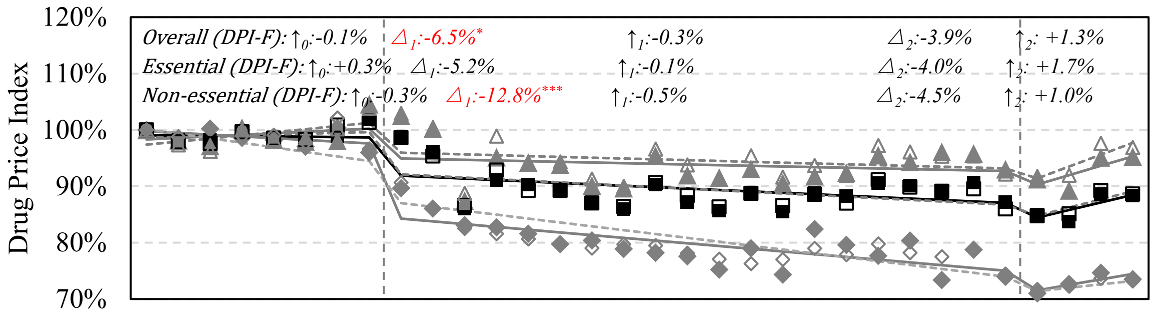

(a) all facilities

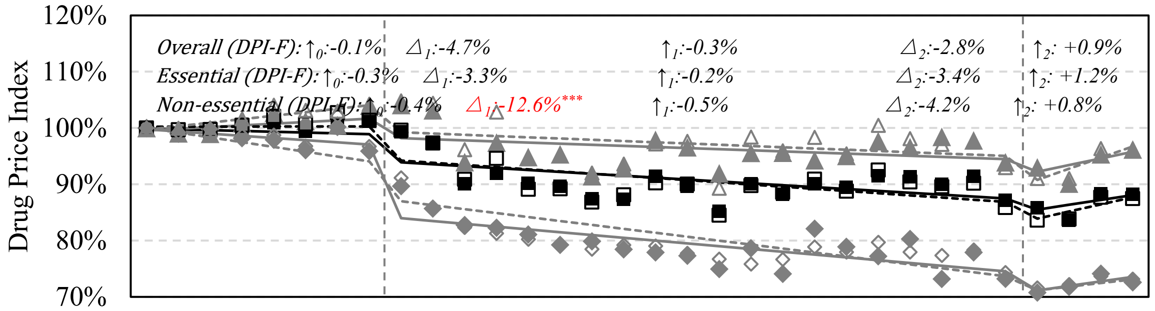

(b) county hospital

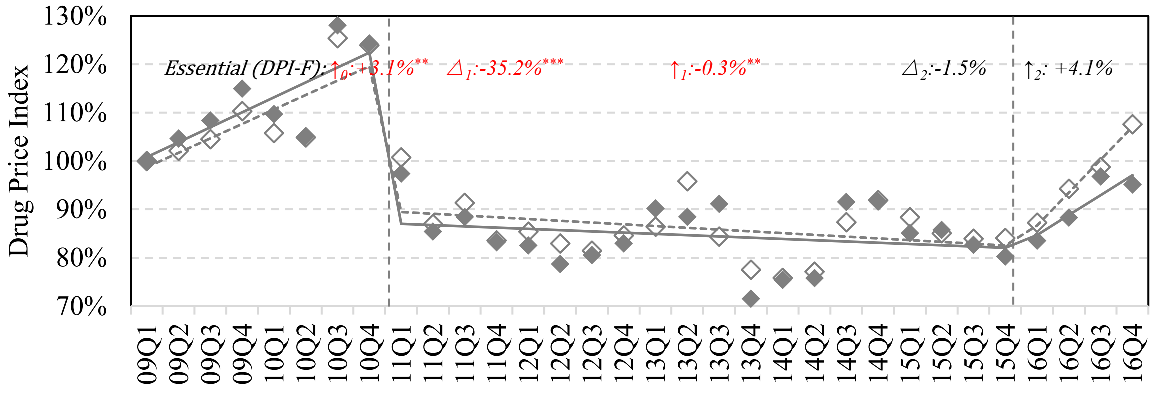

(c) township healthcare centre

Traditional Chinese medicine

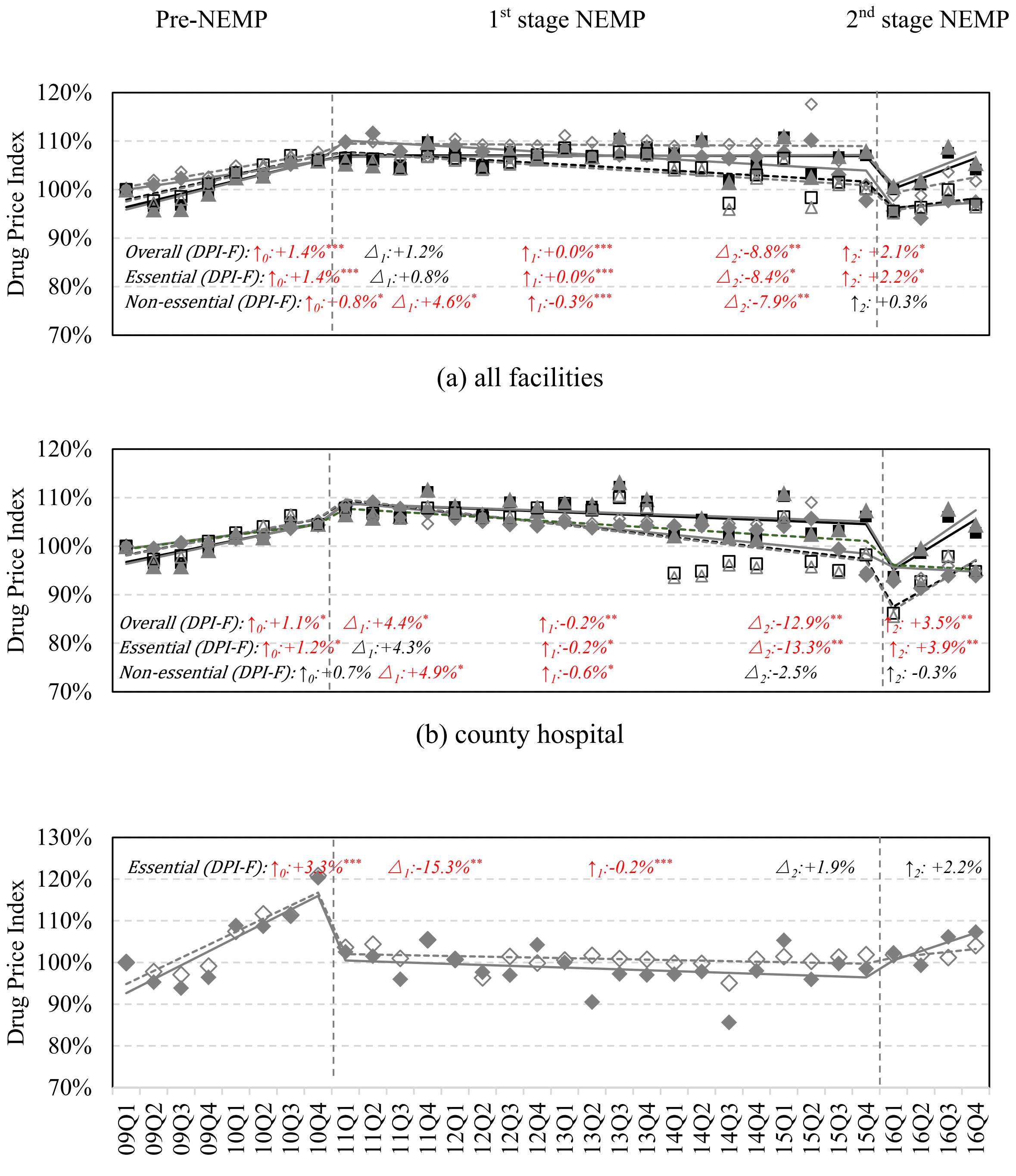

(c) township healthcare centre

↑0: sustained change per observation period before first-phase NEMP (β_1); △1: immediate change after first-phase NEMP (estimator β_3 in ITSA model); ↑1: sustained change per observation period after first-phase NEMP ($\beta_1+\beta_3$); △2: immediate change after second-phase NEMP (β_4); ↑2: sustained change per observation period after second-phase NEMP ($\beta_1+\beta_3+\beta_5$); ITSA, interrupted time-series analysis; NEMP, National Essential Medicines Policy; *, $P<0.05$; **, $P<0.01$; ***, $P<0.001$

Appendix 12. Retail prices of medicines by ATC system for western medicines and TCM classification (Fisher Price Index)

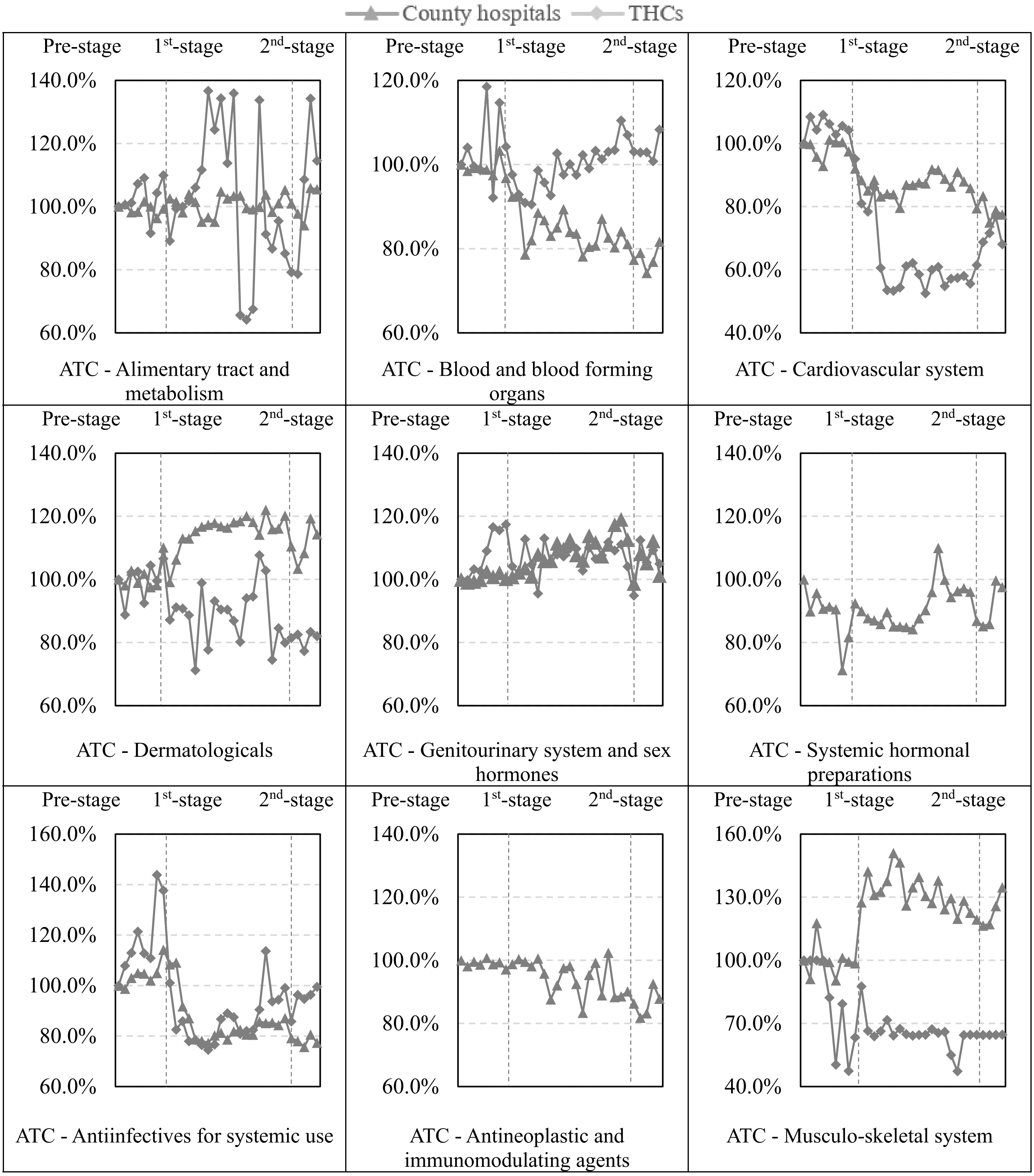

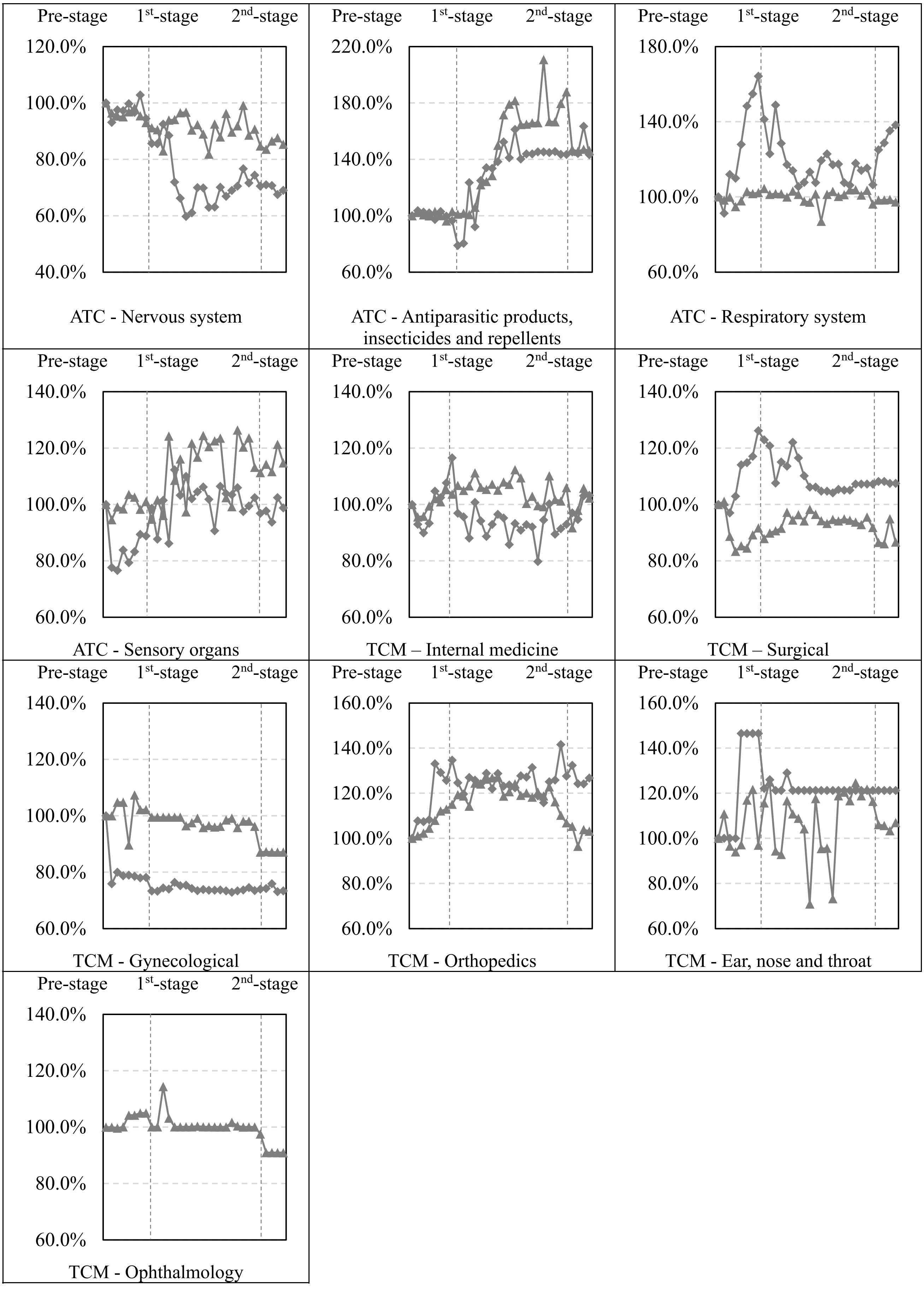
Pre-stage 1st-stage 2nd-stage
120.0%
100.0%
80.0%
60.0%
40.0%
ATC - Nervous system
Pre-stage 1st-stage 2nd-stage
220.0%
180.0%
140.0%
100.0%
60.0%
ATC - Antiparasitic products, insecticides and repellents
Pre-stage 1st-stage 2nd-stage
180.0%
140.0%
100.0%
60.0%
ATC - Respiratory system
Pre-stage 1st-stage 2nd-stage
140.0%
120.0%
100.0%
80.0%
60.0%
ATC - Sensory organs
Pre-stage 1st-stage 2nd-stage
140.0%
120.0%
100.0%
80.0%
60.0%
TCM – Internal medicine
Pre-stage 1st-stage 2nd-stage
140.0%
120.0%
100.0%
80.0%
60.0%
TCM – Surgical
Pre-stage 1st-stage 2nd-stage
140.0%
120.0%
100.0%
80.0%
60.0%
TCM - Gynecological
Pre-stage 1st-stage 2nd-stage
160.0%
140.0%
120.0%
100.0%
80.0%
60.0%
TCM - Orthopedics
Pre-stage 1st-stage 2nd-stage
160.0%
140.0%
120.0%
100.0%
80.0%
60.0%
TCM - Ear, nose and throat
Pre-stage 1st-stage 2nd-stage
140.0%
120.0%
100.0%
80.0%
60.0%
TCM - Ophthalmology

Appendix 13. Interrupted time-series analysis on medicine retail prices

Part I. Drug Price Index – Fisher (DPI-F)

Facility	Drug category	Number of drugs included	Time, $\exp(\beta_1)$-1 (*p-value*)	1st-stage NEMP, $\exp(\beta_2)$-1 (*p-value*)	Time after 1st-stage NEMP, $\exp(\beta_3)$-1 (*p-value*)	2nd-stage NEMP, $\exp(\beta_4)$-1 (*p-value*)	Time after 2nd-stage NEMP, $\exp(\beta_4)$-1 (*p-value*)
County-wide	Overall	405	0.5% (0.146)	-5.3% (0.003)	-0.7% (0.055)	-6.2% (0.027)	1.8% (0.058)
	- Essential drug	321	0.7% (0.035)	-3.6% (0.048)	-0.8% (0.024)	-6.3% (0.036)	2.0% (0.053)
	- Non-essential drug	84	-0.3% (0.512)	-10.0% (<.001)	-0.3% (0.498)	-4.7% (0.147)	1.5% (0.188)
	Western medicine	278	-0.1% (0.910)	-6.5% (0.015)	-0.2% (0.712)	-3.9% (0.291)	1.6% (0.227)
	- Essential drug	230	0.3% (0.628)	-5.2% (0.080)	-0.4% (0.509)	-4.0% (0.354)	1.8% (0.244)
	- Non-essential drug	48	-0.3% (0.410)	-12.8% (<.001)	-0.2% (0.653)	-4.5% (0.174)	1.5% (0.199)
	TCM	127	1.4% (<.001)	1.2% (0.452)	-1.4% (<.001)	-8.8% (0.003)	2.1% (0.036)
	- Essential drug	91	1.4% (<.001)	0.8% (0.663)	-1.4% (<.001)	-8.4% (0.012)	2.2% (0.049)
	- Non-essential drug	36	0.8% (0.024)	4.6% (0.015)	-1.2% (0.004)	-7.9% (0.006)	0.6% (0.495)
	By ATC-classification:						
	- Alimentary tract and metabolism	82	0.0% (0.999)	6.3% (0.053)	-0.2% (0.801)	-12.1% (0.031)	4.1% (0.032)
	- Blood and blood forming organs	35	0.0% (0.958)	-11.4% (<.001)	-0.4% (0.423)	-5.0% (0.251)	1.5% (0.318)
	- Cardiovascular system	47	-0.3% (0.618)	-9.1% (0.004)	0.1% (0.856)	-4.6% (0.238)	0.0% (0.983)
	- Dermatological	35	0.3% (0.589)	1.8% (0.532)	0.6% (0.330)	-22.5% (<.001)	4.5% (0.014)
	- Genito urinary system and sex hormones	20	0.3% (0.621)	-1.6% (0.651)	0.1% (0.846)	-0.1% (0.992)	-2.2% (0.267)
	- Systemic hormonal preparations	9	-2.8% (0.161)	4.9% (0.623)	3.7% (0.076)	-19.6% (0.213)	4.3% (0.431)
	- Anti-infective for systemic use	54	1.8% (0.202)	-10.4% (0.035)	-2.8% (0.094)	2.0% (0.711)	1.0% (0.700)
	- Antineoplastic and immunomodulating agents	6	-0.1% (0.889)	0.8% (0.786)	-0.4% (0.487)	-10.4% (0.056)	3.5% (0.064)
	- Musculo-skeletal system	8	-0.6% (0.731)	30.4% (0.001)	-0.2% (0.920)	-29.2% (0.049)	5.7% (0.255)
	- Nervous system	39	-0.5% (0.386)	-2.8% (0.345)	0.4% (0.488)	-6.3% (0.199)	0.7% (0.661)
	- Antiparasitic products, insecticides and repellents	6	1.0% (0.706)	-4.4% (0.692)	2.9% (0.326)	-25.1% (0.076)	-5.6% (0.323)
	- Respiratory system	34	-2.8% (0.190)	3.3% (0.758)	3.0% (0.175)	6.1% (0.704)	0.0% (0.995)
	- Sensory organs	51	0.9% (0.562)	-0.2% (0.984)	0.4% (0.805)	-7.9% (0.541)	-0.6% (0.890)
	By TCM-classification:						
	- Internal medicine	99	1.3% (0.001)	0.7% (0.697)	-1.3% (0.002)	-8.7% (0.013)	2.2% (0.068)
	- Surgical	8	-1.4% (0.068)	2.2% (0.545)	1.5% (0.059)	-4.5% (0.359)	0.0% (0.982)
	- Gynecological	3	0.1% (0.900)	-2.4% (0.431)	-0.1% (0.921)	-5.6% (0.274)	0.5% (0.763)
	- Orthopedics	9	2.8% (<.001)	3.0% (0.369)	-3.1% (<.001)	-2.2% (0.609)	-1.4% (0.416)
	- Ear, nose and throat	6	1.1% (0.606)	-4.9% (0.660)	-0.6% (0.786)	-6.9% (0.694)	-0.3% (0.959)
	- Ophthalmology	2	0.9% (0.033)	-2.3% (0.286)	-1.1% (0.012)	-7.9% (0.031)	0.2% (0.860)
County hospitals	Overall	383	0.4% (0.171)	-4.0% (0.014)	-0.7% (0.037)	-7.0% (0.006)	2.0% (0.023)
	- Essential drug	300	0.7% (0.026)	-1.5% (0.346)	-0.9% (0.008)	-7.7% (0.003)	2.3% (0.009)

	- Non-essential drug	83	-0.4% (0.385)	-9.7% (<.001)	-0.2% (0.645)	-4.1% (0.209)	1.3% (0.257)
	Western medicine	270	-0.1% (0.778)	-4.7% (0.065)	-0.2% (0.725)	-2.8% (0.395)	1.2% (0.330)
	- Essential drug	223	0.3% (0.565)	-3.3% (0.218)	-0.5% (0.375)	-3.4% (0.360)	1.4% (0.302)
	- Non-essential drug	47	-0.4% (0.316)	-12.6% (<.001)	-0.1% (0.784)	-4.2% (0.207)	1.3% (0.257)
	TCM	113	1.1% (0.011)	4.4% (0.047)	-1.3% (0.004)	-12.9% (0.001)	3.7% (0.005)
	- Essential drug	77	1.2% (0.016)	4.3% (0.084)	-1.4% (0.007)	-13.3% (0.002)	4.1% (0.006)
	- Non-essential drug	36	0.7% (0.095)	4.9% (0.010)	-1.3% (0.015)	-2.5% (0.261)	0.3% (0.782)
	By ATC-classification:						
	- Alimentary tract and metabolism	80	-0.2% (0.643)	1.3% (0.565)	0.3% (0.523)	-9.9% (0.014)	3.5% (0.012)
	- Blood and blood forming organs	35	0.0% (0.983)	-11.2% (<.001)	-0.4% (0.428)	-5.2% (0.243)	1.5% (0.309)
	- Cardiovascular system	46	-0.2% (0.756)	-9.4% (0.009)	0.1% (0.905)	0.1% (0.980)	-2.2% (0.203)
	- Dermatological	34	0.6% (0.382)	7.9% (0.029)	-0.1% (0.840)	-20.0% (0.002)	4.2% (0.042)
	- Genito urinary system and sex hormones	20	0.3% (0.679)	1.7% (0.648)	0.2% (0.795)	-1.0% (0.855)	-2.4% (0.232)
	- Systemic hormonal preparations	9	-2.8% (0.155)	7.1% (0.485)	3.5% (0.092)	-18.3% (0.251)	3.7% (0.495)
	- Anti-infective for systemic use	52	1.1% (0.406)	-6.1% (0.231)	-2.3% (0.172)	-0.8% (0.891)	1.3% (0.626)
	- Antineoplastic and immunomodulating agents	6	-0.1% (0.809)	1.1% (0.725)	-0.4% (0.547)	-10.4% (0.056)	3.5% (0.062)
	- Musculo-skeletal system	8	0.0% (0.987)	31.6% (0.002)	-0.2% (0.895)	-18.7% (0.193)	6.1% (0.219)
	- Nervous system	38	-0.5% (0.411)	-2.8% (0.391)	0.5% (0.480)	-6.2% (0.245)	0.7% (0.716)
	- Antiparasitic products, insecticides and repellents	5	0.5% (0.850)	-2.6% (0.840)	4.3% (0.156)	-44.4% (0.015)	-6.2% (0.334)
	- Respiratory system	34	0.6% (0.700)	-2.2% (0.768)	-0.6% (0.674)	0.7% (0.956)	-0.2% (0.965)
	- Sensory organs	48	0.5% (0.721)	4.2% (0.555)	0.2% (0.863)	-7.5% (0.516)	0.4% (0.921)
	By TCM-classification:						
	- Internal medicine	89	1.1% (0.038)	4.0% (0.138)	-1.4% (0.015)	-13.5% (0.004)	4.2% (0.009)
	- Surgical	7	-1.2% (0.099)	4.2% (0.253)	1.4% (0.074)	-7.2% (0.171)	0.0% (0.991)
	- Gynecological	1	0.2% (0.868)	-2.1% (0.758)	-0.3% (0.839)	-12.5% (0.274)	0.3% (0.948)
	- Orthopedics	8	2.6% (0.005)	7.7% (0.066)	-3.1% (0.002)	-5.5% (0.311)	-1.1% (0.577)
	- Ear, nose and throat	6	1.0% (0.657)	-5.7% (0.634)	-0.5% (0.850)	-8.3% (0.666)	-0.5% (0.943)
	- Ophthalmology	2	0.9% (0.033)	-2.3% (0.286)	-1.1% (0.012)	-7.9% (0.031)	0.2% (0.860)
THCs	Overall - Essential drug	117	3.2% (<.001)	-26.1% (<.001)	-3.4% (<.001)	0.5% (0.913)	3.5% (0.050)
	Western medicine - Essential drug	68	3.1% (0.007)	-35.2% (<.001)	-3.4% (0.005)	-1.5% (0.859)	4.4% (0.140)
	TCM - Essential drug	49	3.3% (<.001)	-15.3% (<.001)	-3.5% (<.001)	1.9% (0.751)	2.4% (0.251)
	By ATC-classification:						
	- Alimentary tract and metabolism	25	1.5% (0.680)	2.8% (0.879)	-2.5% (0.496)	-12.7% (0.636)	13.3% (0.164)
	- Blood and blood forming organs	8	1.2% (0.005)	-17.2% (<.001)	-0.4% (0.336)	-7.3% (0.055)	0.6% (0.651)
	- Cardiovascular system	9	-0.9% (0.629)	-12.3% (0.110)	-0.7% (0.759)	9.1% (0.317)	2.9% (0.454)
	- Dermatological	9	1.1% (0.357)	-14.7% (0.028)	-1.2% (0.353)	-8.5% (0.429)	0.7% (0.844)
	- Genito urinary system and sex hormones	4	3.0% (<.001)	-12.4% (<.001)	-2.9% (<.001)	2.7% (0.626)	-1.0% (0.594)
	- Systemic hormonal preparations	0	n.a.	n.a.	n.a.	n.a.	n.a.
	- Anti-infective for systemic use	20	4.8% (0.004)	-53.4% (<.001)	-4.2% (0.013)	3.0% (0.799)	0.1% (0.974)
	- Antineoplastic and immunomodulating agents	0	n.a.	n.a.	n.a.	n.a.	n.a.

	- Musculo-skeletal system	2	-7.7% (<.001)	15.9% (0.008)	7.1% (<.001)	6.0% (0.543)	0.3% (0.921)
	- Nervous system	7	-1.1% (0.452)	-10.1% (0.094)	0.3% (0.854)	1.7% (0.809)	0.7% (0.821)
	- Antiparasitic products, insecticides and repellents	4	0.7% (0.784)	-4.7% (0.716)	2.2% (0.436)	-4.6% (0.798)	-4.6% (0.475)
	- Respiratory system	8	10.0% (<.001)	-31.1% (<.001)	-11.2% (<.001)	13.8% (0.249)	5.7% (0.181)
	- Sensory organs	16	0.1% (0.899)	14.5% (0.008)	0.0% (0.977)	-8.7% (0.321)	1.5% (0.624)
	By TCM-classification:						
	- Internal medicine	36	2.9% (<.001)	-16.4% (<.001)	-3.1% (<.001)	1.8% (0.775)	2.9% (0.189)
	- Surgical	3	3.7% (<.001)	-4.0% (0.312)	-4.4% (<.001)	3.3% (0.545)	1.0% (0.612)
	- Gynecological	2	-1.5% (0.002)	-0.7% (0.757)	1.5% (0.003)	2.4% (0.546)	-0.6% (0.658)
	- Orthopedics	7	5.0% (<.001)	-12.7% (0.006)	-4.8% (<.001)	3.3% (0.641)	-1.9% (0.441)
	- Ear, nose and throat	1	8.8% (<.001)	-30.8% (<.001)	-9.0% (<.001)	0.6% (0.940)	0.1% (0.961)
	- Ophthalmology	0	n.a.	n.a.	n.a.	n.a.	n.a.

Part II. Drug Price Index – Laspeyres (DPI-L)

Facility	Drug category	Number of drugs included	Time, exp(β_1)-1 (*p-value*)	1st-stage NEMP, exp(β_2)-1 (*p-value*)	Time after 1st-stage NEMP, exp(β_3)-1 (*p-value*)	2nd-stage NEMP, exp(β_4)-1 (*p-value*)	Time after 2nd-stage NEMP, exp(β_4)-1 (*p-value*)
County-wide	Overall	405	0.6% (0.071)	-6.1% (<.001)	-0.8% (0.013)	-4.7% (0.083)	1.7% (0.073)
	- Essential drug	321	0.9% (0.011)	-3.6% (0.039)	-1.1% (0.003)	-4.9% (0.095)	2.0% (0.052)
	- Non-essential drug	84	-0.7% (0.193)	-6.1% (0.005)	0.0% (0.984)	-3.2% (0.195)	1.2% (0.255)
	Western medicine	278	0.1% (0.748)	-7.2% (0.004)	-0.4% (0.366)	-3.4% (0.328)	1.7% (0.157)
	- Essential drug	230	0.5% (0.266)	-5.1% (0.049)	-0.7% (0.175)	-3.9% (0.351)	2.2% (0.122)
	- Non-essential drug	48	-0.8% (0.137)	-6.8% (0.004)	0.1% (0.843)	-3.2% (0.228)	1.3% (0.259)
	TCM	127	1.3% (0.003)	1.1% (0.570)	-1.6% (<.001)	-6.2% (0.059)	1.0% (0.367)
	- Essential drug	91	1.3% (0.004)	1.0% (0.635)	-1.7% (<.001)	-5.8% (0.105)	1.0% (0.400)
	- Non-essential drug	36	1.0% (0.033)	2.1% (0.365)	-1.0% (0.035)	-10.7% (0.006)	1.1% (0.380)
	By ATC-classification:						
	- Alimentary tract and metabolism	82	0.1% (0.934)	6.3% (0.085)	-0.3% (0.650)	-13.2% (0.040)	5.2% (0.022)
	- Blood and blood forming organs	35	0.0% (0.926)	-12.7% (<.001)	-0.2% (0.453)	-5.7% (0.045)	1.8% (0.060)
	- Cardiovascular system	47	0.3% (0.592)	-15.9% (<.001)	-0.5% (0.297)	-4.3% (0.201)	-0.1% (0.903)
	- Dermatological	35	0.2% (0.576)	-2.7% (0.210)	1.0% (0.024)	-20.6% (<.001)	4.5% (0.002)
	- Genito urinary system and sex hormones	20	0.4% (0.471)	-3.4% (0.295)	0.1% (0.934)	1.9% (0.705)	-0.9% (0.598)
	- Systemic hormonal preparations	9	-2.8% (0.135)	2.6% (0.785)	4.3% (0.034)	-18.9% (0.232)	4.6% (0.395)
	- Anti-infective for systemic use	54	1.6% (0.234)	-8.0% (0.078)	-2.9% (0.088)	-1.7% (0.737)	2.3% (0.360)
	- Antineoplastic and immunomodulating agents	6	0.1% (0.797)	-0.2% (0.932)	-0.6% (0.222)	-9.7% (0.027)	3.4% (0.025)
	- Musculo-skeletal system	8	0.9% (0.613)	12.9% (0.155)	-0.6% (0.744)	-45.3% (0.006)	9.5% (0.082)
	- Nervous system	39	-0.4% (0.683)	1.4% (0.757)	0.3% (0.721)	-2.2% (0.730)	-0.5% (0.826)

	- Antiparasitic products, insecticides and repellents	6	0.7% (0.758)	-4.1% (0.701)	3.0% (0.293)	-22.3% (0.100)	-5.2% (0.334)
	- Respiratory system	34	-2.8% (0.123)	5.7% (0.531)	3.2% (0.099)	5.2% (0.699)	0.3% (0.946)
	- Sensory organs	51	0.3% (0.885)	6.1% (0.501)	1.0% (0.604)	-7.1% (0.603)	0.7% (0.884)
	By TCM-classification:						
	- Internal medicine	99	1.0% (0.029)	1.3% (0.587)	-1.4% (0.005)	-5.6% (0.144)	1.3% (0.318)
	- Surgical	8	2.4% (0.003)	-0.4% (0.909)	-2.2% (0.014)	-7.4% (0.070)	-0.8% (0.623)
	- Gynecological	3	-0.4% (0.601)	-1.5% (0.688)	0.2% (0.751)	-6.5% (0.308)	0.5% (0.802)
	- Orthopedics	9	2.5% (<.001)	0.8% (0.733)	-2.7% (<.001)	-4.8% (0.141)	-0.8% (0.524)
	- Ear, nose and throat	6	1.0% (0.662)	-5.3% (0.642)	-0.4% (0.867)	-6.1% (0.739)	-0.3% (0.957)
	- Ophthalmology	2	0.7% (0.065)	-1.5% (0.446)	-0.9% (0.027)	-8.2% (0.015)	0.2% (0.863)
County hospitals	Overall	383	0.5% (0.138)	-4.8% (0.007)	-0.9% (0.012)	-7.1% (0.011)	2.3% (0.018)
	- Essential drug	300	0.9% (0.010)	-2.1% (0.222)	-1.3% (<.001)	-8.8% (0.004)	3.0% (0.005)
	- Non-essential drug	83	-0.7% (0.177)	-5.7% (0.013)	0.1% (0.933)	-3.1% (0.240)	1.3% (0.266)
	Western medicine	270	0.0% (0.984)	-5.7% (0.032)	-0.4% (0.475)	-4.2% (0.236)	1.7% (0.200)
	- Essential drug	223	0.6% (0.256)	-4.6% (0.107)	-0.8% (0.142)	-6.2% (0.162)	2.2% (0.154)
	- Non-essential drug	47	-0.9% (0.143)	-6.4% (0.009)	0.2% (0.822)	-3.1% (0.258)	1.3% (0.267)
	TCM	113	1.1% (0.124)	4.5% (0.187)	-1.7% (0.024)	-12.9% (0.012)	3.8% (0.036)
	- Essential drug	77	1.1% (0.146)	4.6% (0.215)	-1.8% (0.031)	-13.5% (0.016)	4.1% (0.037)
	- Non-essential drug	36	0.7% (0.110)	3.6% (0.111)	-1.1% (0.034)	-4.6% (0.125)	0.1% (0.961)
	By ATC-classification:						
	- Alimentary tract and metabolism	80	-0.1% (0.760)	-0.5% (0.801)	0.3% (0.528)	-13.1% (0.002)	4.6% (0.002)
	- Blood and blood forming organs	35	0.1% (0.859)	-12.6% (<.001)	-0.3% (0.426)	-5.9% (0.048)	2.0% (0.055)
	- Cardiovascular system	46	0.4% (0.524)	-14.4% (<.001)	-0.7% (0.230)	-4.4% (0.284)	-0.2% (0.864)
	- Dermatological	34	0.4% (0.413)	3.3% (0.198)	0.5% (0.370)	-20.0% (<.001)	4.6% (0.004)
	- Genito urinary system and sex hormones	20	0.5% (0.408)	0.0% (0.988)	0.0% (0.980)	0.0% (0.995)	-1.0% (0.530)
	- Systemic hormonal preparations	9	-2.9% (0.129)	4.3% (0.657)	4.0% (0.044)	-17.3% (0.267)	3.6% (0.501)
	- Anti-infective for systemic use	52	1.1% (0.415)	-3.5% (0.417)	-2.5% (0.137)	-5.0% (0.315)	2.3% (0.346)
	- Antineoplastic and immunomodulating agents	6	0.1% (0.790)	-0.3% (0.920)	-0.6% (0.216)	-9.7% (0.027)	3.4% (0.025)
	- Musculo-skeletal system	8	1.0% (0.180)	2.0% (0.611)	0.3% (0.738)	-33.5% (<.001)	12.7% (<.001)
	- Nervous system	38	-0.3% (0.724)	1.5% (0.756)	0.3% (0.771)	-3.8% (0.584)	-0.2% (0.938)
	- Antiparasitic products, insecticides and repellents	5	0.5% (0.858)	-4.3% (0.726)	4.2% (0.142)	-40.9% (0.017)	-6.0% (0.324)
	- Respiratory system	34	0.7% (0.684)	-5.0% (0.581)	-0.8% (0.660)	0.6% (0.966)	0.6% (0.911)
	- Sensory organs	48	-0.2% (0.846)	7.6% (0.254)	1.0% (0.449)	-11.7% (0.291)	2.4% (0.524)
	By TCM-classification:						
	- Internal medicine	89	0.8% (0.338)	4.4% (0.297)	-1.5% (0.090)	-14.2% (0.025)	4.8% (0.030)
	- Surgical	7	3.4% (<.001)	0.4% (0.916)	-3.0% (0.001)	-12.4% (0.009)	-1.6% (0.345)
	- Gynecological	1	0.2% (0.868)	-2.1% (0.758)	-0.3% (0.839)	-12.5% (0.274)	0.3% (0.948)
	- Orthopedics	8	2.2% (0.001)	5.3% (0.050)	-2.6% (0.001)	-3.3% (0.297)	-0.6% (0.637)
	- Ear, nose and throat	6	0.8% (0.747)	-5.1% (0.687)	-0.2% (0.943)	-7.6% (0.708)	-0.5% (0.941)

	- Ophthalmology	2	0.7% (0.065)	-1.5% (0.446)	-0.9% (0.027)	-8.2% (0.015)	0.2% (0.863)
THCs	Overall - Essential drug	117	2.9% (<.001)	-22.7% (<.001)	-3.2% (<.001)	-1.8% (0.677)	4.7% (0.007)
	Western medicine - Essential drug	68	3.0% (0.007)	-29.8% (<.001)	-3.3% (0.005)	-2.7% (0.723)	7.2% (0.011)
	TCM - Essential drug	49	3.1% (<.001)	-14.6% (<.001)	-3.3% (<.001)	0.8% (0.827)	0.8% (0.517)
	By ATC-classification:						
	- Alimentary tract and metabolism	25	1.7% (0.641)	-2.2% (0.904)	-2.1% (0.584)	-14.2% (0.601)	18.9% (0.054)
	- Blood and blood forming organs	8	1.5% (0.014)	-18.3% (<.001)	-0.9% (0.125)	0.7% (0.898)	-1.6% (0.401)
	- Cardiovascular system	9	-0.7% (0.709)	-12.3% (0.088)	-1.4% (0.527)	21.2% (0.016)	2.8% (0.449)
	- Dermatological	9	-0.8% (0.454)	-5.7% (0.276)	0.9% (0.423)	-2.9% (0.725)	-0.2% (0.947)
	- Genito urinary system and sex hormones	4	2.8% (<.001)	-12.6% (<.001)	-2.9% (<.001)	-1.0% (0.848)	0.0% (0.990)
	- Systemic hormonal preparations	0	0.0% (<.001)	0.0% (<.001)	0.0% (<.001)	0.0% (<.001)	0.0% (<.001)
	- Anti-infective for systemic use	20	4.8% (<.001)	-42.4% (<.001)	-4.7% (0.001)	-6.7% (0.509)	6.3% (0.079)
	- Antineoplastic and immunomodulating agents	0	0.0% (<.001)	0.0% (<.001)	0.0% (<.001)	0.0% (<.001)	0.0% (<.001)
	- Musculo-skeletal system	2	-9.0% (<.001)	23.0% (<.001)	8.5% (<.001)	6.4% (0.548)	0.1% (0.979)
	- Nervous system	7	-1.3% (0.459)	-9.0% (0.157)	0.1% (0.945)	-6.5% (0.383)	1.9% (0.581)
	- Antiparasitic products, insecticides and repellents	4	-0.4% (0.874)	3.1% (0.823)	3.1% (0.283)	-8.1% (0.680)	-3.1% (0.651)
	- Respiratory system	8	10.1% (<.001)	-31.9% (<.001)	-10.8% (<.001)	16.4% (0.160)	9.6% (0.024)
	- Sensory organs	16	-1.1% (0.525)	34.7% (<.001)	1.4% (0.453)	-3.5% (0.810)	0.3% (0.957)
	By TCM-classification:						
	- Internal medicine	36	2.8% (<.001)	-15.9% (<.001)	-2.9% (<.001)	1.1% (0.819)	1.1% (0.517)
	- Surgical	3	4.0% (<.001)	-8.0% (<.001)	-4.2% (<.001)	0.6% (0.847)	0.3% (0.824)
	- Gynecological	2	-1.2% (0.003)	-2.4% (0.223)	1.2% (0.005)	0.3% (0.924)	0.0% (0.975)
	- Orthopedics	7	4.5% (<.001)	-11.4% (<.001)	-4.5% (<.001)	-0.2% (0.924)	0.0% (0.985)
	- Ear, nose and throat	1	8.8% (<.001)	-30.8% (<.001)	-9.0% (<.001)	0.6% (0.940)	0.1% (0.961)
	- Ophthalmology	0	0.0% (<.001)	0.0% (<.001)	0.0% (<.001)	0.0% (<.001)	0.0% (<.001)

Note: Segmented linear regression model was built with two interruption points: $Y_t = \beta_0 + \beta_1 T + \beta_2 X_{1\_t} + \beta_3 T X_{1\_t} + \beta_4 X_{2\_t} + \beta_5 T X_{2\_t} + \varepsilon_t$, Coefficient β0 estimates the baseline level of outcome; β1 estimates the time trend of outcome in the pre-policy period; β2 and β4 estimate the immediate changes in level after the first- and second-stage policy; and β3 and β5¬ estimate the sustained change in trend after the first- and second-stage policy; ATC, Anatomical Therapeutic Chemical; TCM, Traditional Chinese Medicine; NEMP, National Essential Medicines Policy; *, P<0.05; **, P<0.01; ***, P<0.001

Appendix 14. Wholesale prices of medicines by western or Traditional Chinese medicines

■ overall (DPI-F) ▲ essential medicines (DPI-F) ♦ non-essential medicines (DPI-F)

□ overall (DPI-L) △ essential medicines (DPI-L) ◊ non-essential medicines (DPI-L)

▬ model fitted line

Western medicine

Pre-NEMP 1st stage NEMP 2nd stage NEMP

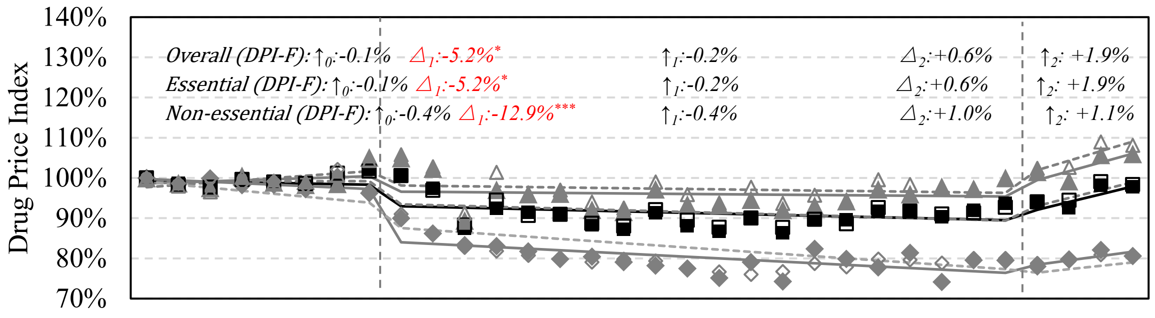

(a) all facilities

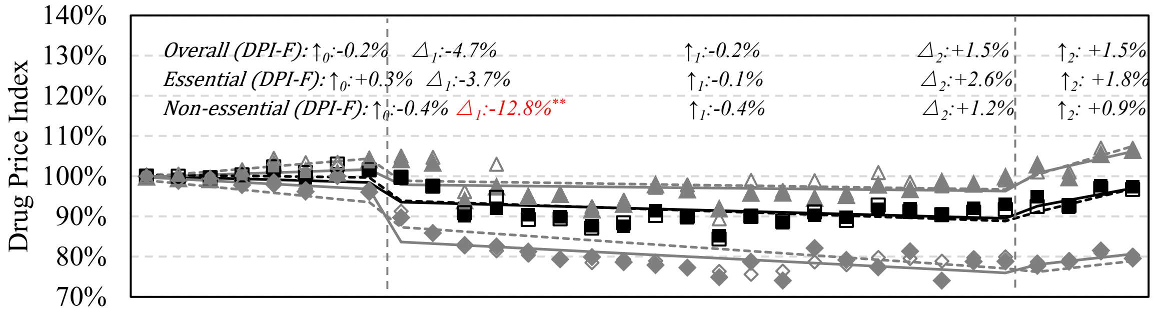

(b) county hospital

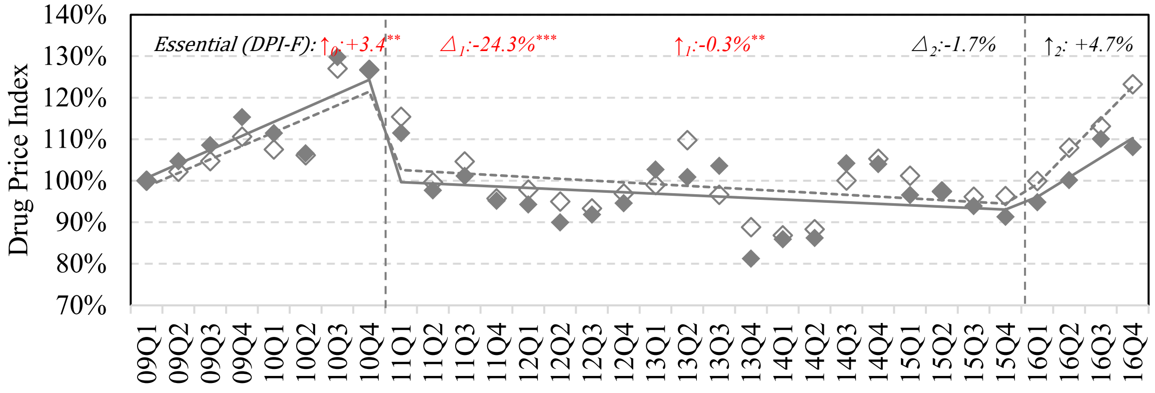

(c) township healthcare centre

Traditional Chinese medicine

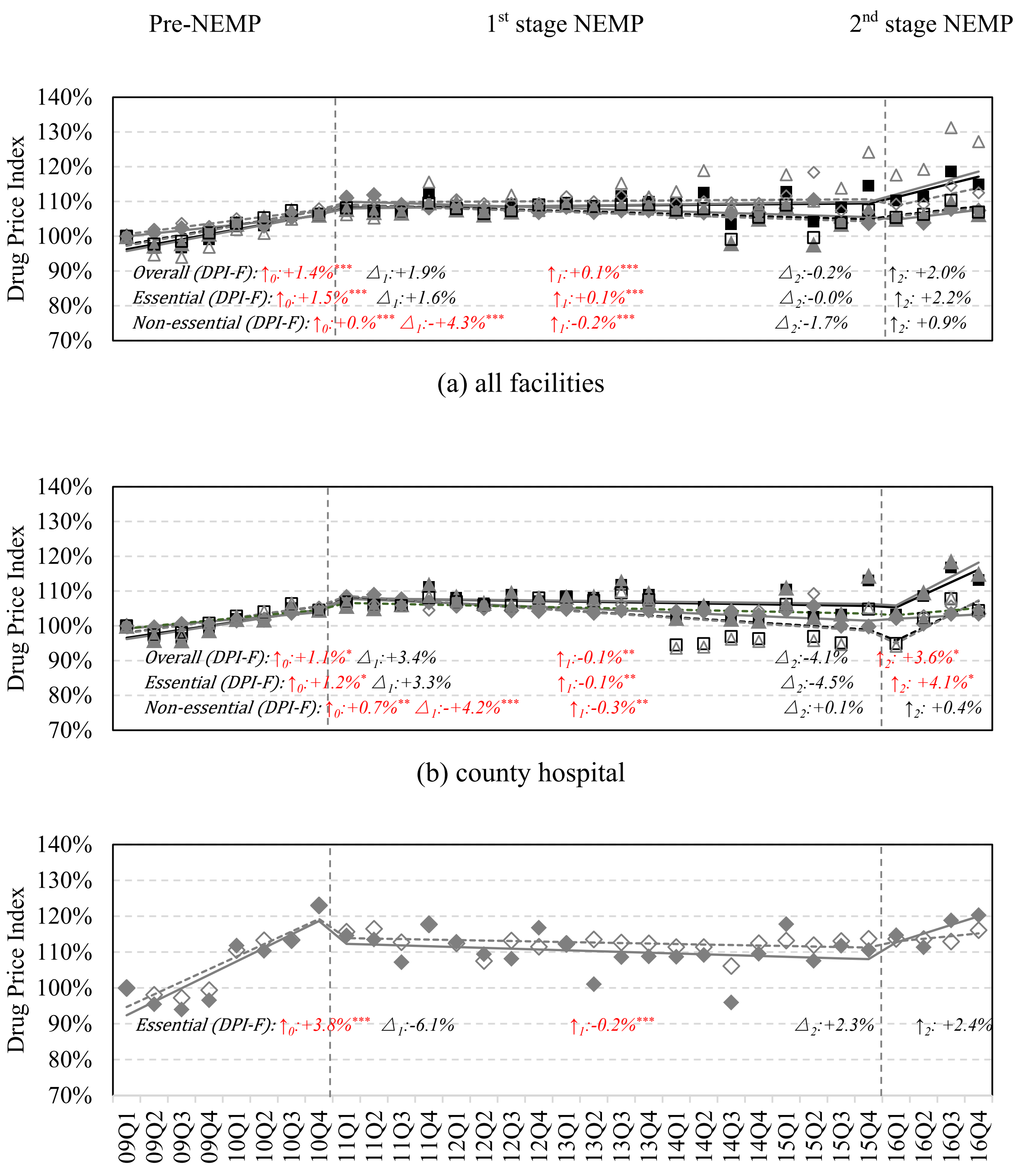

(a) all facilities

(b) county hospital

(c) township healthcare centre

$\uparrow_0$: sustained change per observation period before first-phase NEMP (β_1); $\triangle_1$: immediate change after first-phase NEMP (estimator β_3 in ITSA model); $\uparrow_1$: sustained change per observation period after first-phase NEMP ($\beta_1+\beta_3$); $\triangle_2$: immediate change after second-phase NEMP (β_4); $\uparrow_2$: sustained change per observation period after second-phase NEMP ($\beta_1+\beta_3+\beta_5$); ITSA, interrupted time-series analysis; NEMP, National Essential Medicines Policy; *, $P<0.05$; **, $P<0.01$; ***, $P<0.001$

Appendix 15. Wholesale prices of medicines by ATC system for western medicines and TCM classification (Fisher Price Index)

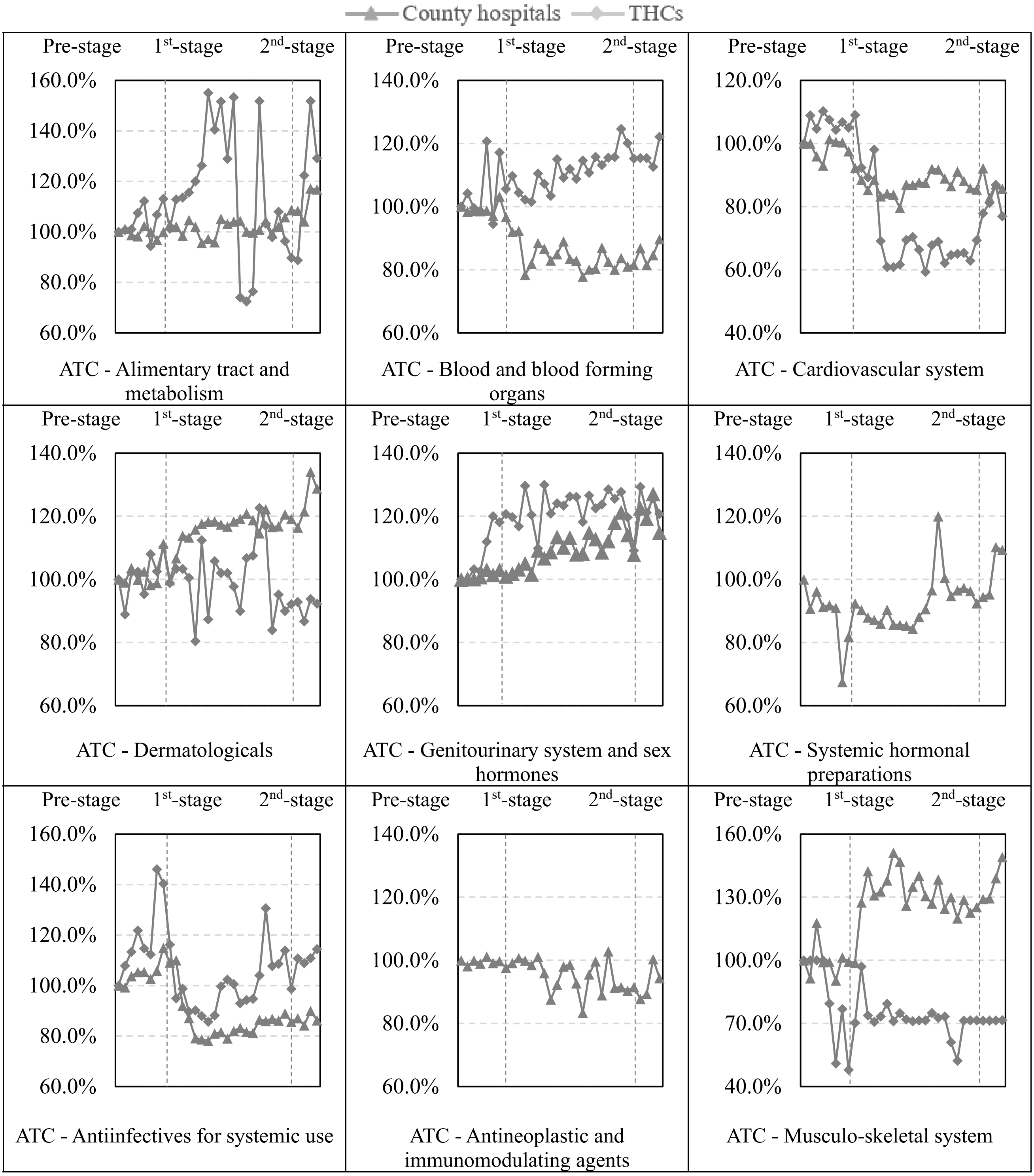

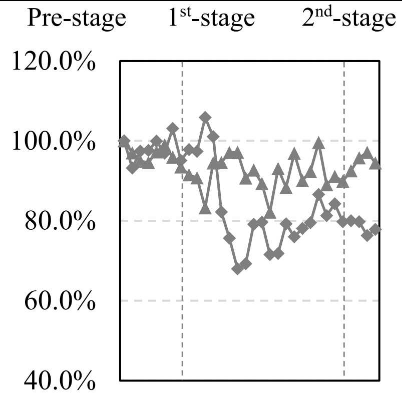

ATC - Nervous system

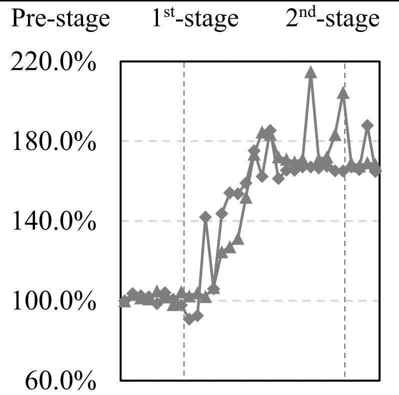

ATC - Antiparasitic products, insecticides and repellents

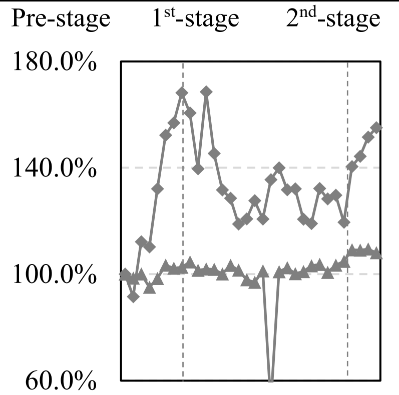

ATC - Respiratory system

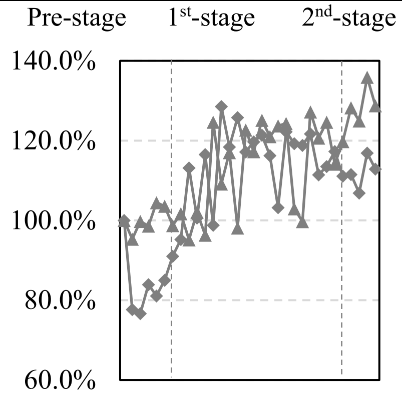

ATC - Sensory organs

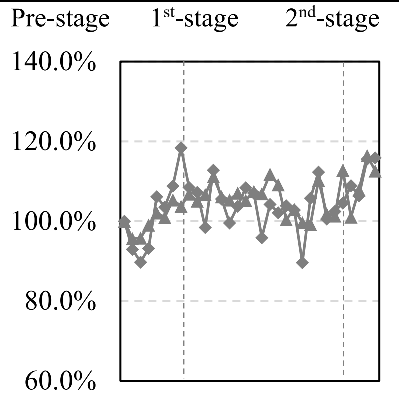

TCM – Internal medicine

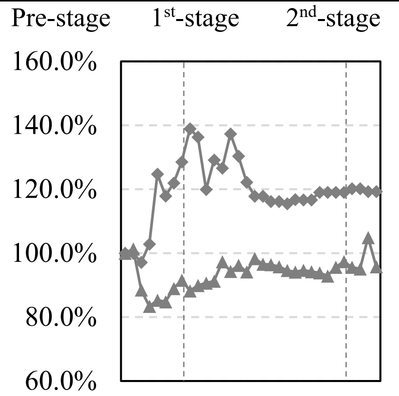

TCM – Surgical

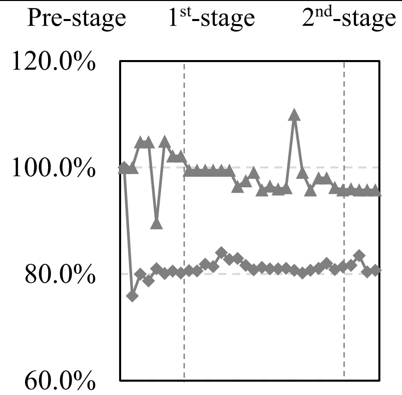

TCM - Gynecological

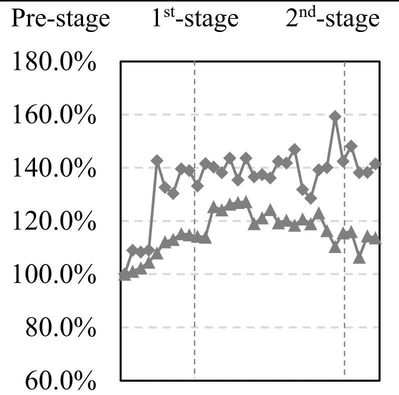

TCM - Orthopedics

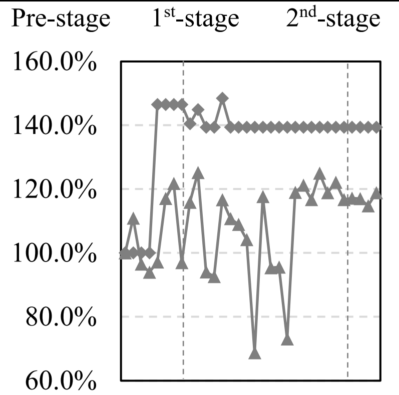

TCM - Ear, nose and throat

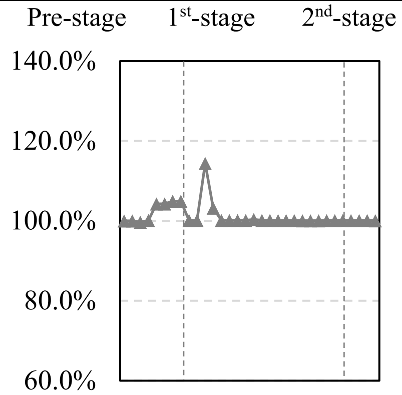

TCM - Ophthalmology

Appendix 16. Interrupted time-series analysis on medicine wholesale prices

Part I. Drug Price Index – Fisher (DPI-F)

Facility	Drug category	Number of drugs included	Time, exp(β1)-1 (*p-value*)	1st-stage NEMP, exp(β2)-1 (*p-value*)	Time after 1st-stage NEMP, exp(β3)-1 (*p-value*)	2nd-stage NEMP, exp(β4)-1 (*p-value*)	Time after 2nd-stage NEMP, exp(β4)-1 (*p-value*)
County-wide	Overall	405	0.4% (0.283)	-4.2% (0.042)	-0.5% (0.194)	0.1% (0.969)	2.0% (0.064)
	- Essential drug	321	0.7% (0.067)	-2.4% (0.251)	-0.8% (0.069)	0.9% (0.789)	2.2% (0.061)
	- Non-essential drug	84	-0.3% (0.441)	-9.7% (<.001)	-0.1% (0.840)	0.2% (0.948)	1.7% (0.168)
	Western medicine	278	-0.1% (0.800)	-5.2% (0.076)	0.0% (0.953)	0.6% (0.866)	2.1% (0.139)
	- Essential drug	230	-0.1% (0.800)	-5.2% (0.076)	0.0% (0.953)	0.6% (0.866)	2.1% (0.139)
	- Non-essential drug	48	-0.4% (0.414)	-12.9% (<.001)	0.0% (0.931)	1.0% (0.789)	1.5% (0.241)
	TCM	127	1.4% (<.001)	1.9% (0.289)	-1.4% (<.001)	-0.2% (0.945)	1.9% (0.088)
	- Essential drug	91	1.5% (<.001)	1.6% (0.432)	-1.4% (0.002)	0.0% (0.998)	2.1% (0.103)
	- Non-essential drug	36	0.8% (<.001)	4.3% (<.001)	-1.1% (<.001)	-1.7% (0.377)	1.2% (0.081)
	By ATC-classification:						
	- Alimentary tract and metabolism	82	0.1% (0.905)	6.8% (0.067)	-0.2% (0.813)	-4.2% (0.484)	4.3% (0.044)
	- Blood and blood forming organs	35	0.0% (0.997)	-11.5% (<.001)	-0.3% (0.534)	2.2% (0.618)	1.5% (0.332)
	- Cardiovascular system	47	0.0% (0.997)	-11.5% (<.001)	-0.3% (0.534)	2.2% (0.618)	1.5% (0.332)
	- Dermatological	35	0.4% (0.410)	2.9% (0.196)	0.5% (0.263)	-11.7% (0.007)	4.9% (0.002)
	- Genito urinary system and sex hormones	20	0.4% (0.422)	0.4% (0.874)	0.1% (0.800)	7.4% (0.097)	-1.6% (0.279)
	- Systemic hormonal preparations	9	-3.0% (0.134)	6.1% (0.544)	4.0% (0.058)	-14.0% (0.380)	5.4% (0.326)
	- Anti-infective for systemic use	54	1.9% (0.183)	-8.1% (0.096)	-2.8% (0.107)	4.0% (0.462)	0.9% (0.736)
	- Antineoplastic and immunomodulating agents	6	0.0% (0.977)	-0.2% (0.958)	-0.4% (0.547)	-6.1% (0.257)	3.6% (0.060)
	- Musculo-skeletal system	8	-0.6% (0.690)	33.1% (<.001)	-0.1% (0.961)	-22.5% (0.116)	6.1% (0.215)
	- Nervous system	39	-0.4% (0.456)	-3.2% (0.259)	0.4% (0.497)	1.2% (0.799)	0.8% (0.637)
	- Antiparasitic products, insecticides and repellents	6	1.2% (0.641)	-3.3% (0.770)	3.3% (0.275)	-21.1% (0.140)	-5.8% (0.313)
	- Respiratory system	34	-2.8% (0.223)	4.1% (0.715)	3.0% (0.203)	12.4% (0.459)	0.5% (0.935)
	- Sensory organs	51	1.0% (0.494)	2.0% (0.802)	0.3% (0.837)	2.1% (0.866)	-0.3% (0.951)
	By TCM-classification:						
	- Internal medicine	99	1.4% (0.002)	1.5% (0.487)	-1.3% (0.003)	-0.1% (0.975)	2.1% (0.116)
	- Surgical	8	-1.4% (0.049)	4.1% (0.253)	1.7% (0.032)	-0.4% (0.928)	0.6% (0.744)
	- Gynecological	3	0.1% (0.881)	1.8% (0.522)	-0.1% (0.844)	0.6% (0.902)	0.5% (0.781)
	- Orthopedics	9	3.0% (<.001)	1.9% (0.568)	-3.1% (0.001)	0.5% (0.913)	-1.3% (0.445)
	- Ear, nose and throat	6	1.1% (0.637)	-3.5% (0.760)	-0.5% (0.815)	2.9% (0.877)	0.0% (0.999)
	- Ophthalmology	2	0.9% (0.035)	-2.5% (0.256)	-1.1% (0.014)	1.0% (0.783)	0.2% (0.874)
County hospitals	Overall	383	0.3% (0.344)	-4.3% (0.024)	-0.5% (0.201)	-0.8% (0.775)	2.3% (0.021)
	- Essential drug	300	0.7% (0.061)	-2.2% (0.233)	-0.7% (0.051)	-0.8% (0.766)	2.7% (0.008)

	- Non-essential drug	83	-0.4% (0.363)	-9.8% (<.001)	0.0% (0.941)	0.6% (0.860)	1.5% (0.227)
	Western medicine	270	-0.2% (0.690)	-4.7% (0.079)	0.0% (0.990)	1.5% (0.646)	1.7% (0.190)
	- Essential drug	223	0.3% (0.630)	-3.7% (0.182)	-0.3% (0.558)	2.6% (0.491)	1.9% (0.173)
	- Non-essential drug	47	-0.4% (0.339)	-12.8% (<.001)	0.0% (0.971)	1.2% (0.742)	1.3% (0.305)
	TCM	113	1.1% (0.016)	3.4% (0.143)	-1.3% (0.010)	-4.1% (0.306)	3.7% (0.010)
	- Essential drug	77	1.2% (0.023)	3.3% (0.212)	-1.3% (0.019)	-4.5% (0.310)	4.2% (0.010)
	- Non-essential drug	36	0.7% (0.005)	4.2% (<.001)	-1.0% (<.001)	0.1% (0.933)	0.7% (0.204)
	By ATC-classification:						
	- Alimentary tract and metabolism	80	-0.2% (0.729)	0.7% (0.784)	0.4% (0.479)	-1.8% (0.679)	3.6% (0.019)
	- Blood and blood forming organs	35	0.0% (0.991)	-11.4% (<.001)	-0.3% (0.522)	2.2% (0.626)	1.5% (0.328)
	- Cardiovascular system	46	-0.1% (0.835)	-11.0% (<.001)	0.1% (0.810)	4.6% (0.310)	-1.9% (0.226)
	- Dermatological	34	0.6% (0.302)	6.9% (0.034)	-0.1% (0.894)	-9.2% (0.089)	4.6% (0.017)
	- Genito urinary system and sex hormones	20	0.3% (0.540)	1.1% (0.692)	0.3% (0.571)	8.2% (0.078)	-1.9% (0.228)
	- Systemic hormonal preparations	9	-3.1% (0.126)	7.6% (0.455)	3.9% (0.067)	-12.7% (0.430)	4.7% (0.394)
	- Anti-infective for systemic use	52	1.3% (0.379)	-6.2% (0.224)	-2.2% (0.205)	2.0% (0.735)	1.3% (0.635)
	- Antineoplastic and immunomodulating agents	6	-0.1% (0.896)	0.1% (0.978)	-0.3% (0.611)	-6.2% (0.255)	3.6% (0.059)
	- Musculo-skeletal system	8	-0.1% (0.964)	31.8% (0.001)	0.0% (0.978)	-9.4% (0.506)	6.9% (0.163)
	- Nervous system	38	-0.4% (0.479)	-3.5% (0.275)	0.4% (0.477)	1.3% (0.799)	0.7% (0.690)
	- Antiparasitic products, insecticides and repellents	5	0.6% (0.816)	-4.3% (0.741)	4.6% (0.121)	-35.9% (0.045)	-5.6% (0.380)
	- Respiratory system	34	0.6% (0.685)	-3.3% (0.669)	-0.6% (0.705)	10.5% (0.416)	-0.4% (0.937)
	- Sensory organs	48	0.5% (0.693)	3.3% (0.632)	0.3% (0.806)	3.6% (0.748)	0.5% (0.899)
	By TCM-classification:						
	- Internal medicine	89	3.2% (<.001)	-6.8% (0.108)	-3.4% (<.001)	2.2% (0.755)	3.2% (0.187)
	- Surgical	7	-1.3% (0.080)	4.1% (0.256)	1.6% (0.044)	-1.3% (0.810)	0.4% (0.841)
	- Gynecological	1	0.1% (0.919)	-2.3% (0.722)	-0.1% (0.960)	-4.8% (0.654)	0.0% (0.999)
	- Orthopedics	8	2.7% (0.003)	4.0% (0.327)	-2.8% (0.004)	-1.9% (0.733)	-0.7% (0.713)
	- Ear, nose and throat	6	1.0% (0.660)	-6.1% (0.617)	-0.4% (0.857)	2.3% (0.904)	-0.3% (0.970)
	- Ophthalmology	2	0.9% (0.035)	-2.5% (0.256)	-1.1% (0.014)	1.0% (0.783)	0.2% (0.874)
THCs	Overall - Essential drug	117	3.5% (<.001)	-15.9% (<.001)	-3.8% (<.001)	0.6% (0.905)	4.0% (0.043)
	Western medicine - Essential drug	68	3.4% (0.008)	-24.3% (<.001)	-3.7% (0.006)	-1.7% (0.856)	5.1% (0.124)
	TCM - Essential drug	49	3.8% (<.001)	-6.1% (0.138)	-4.0% (<.001)	2.3% (0.735)	2.6% (0.258)
	By ATC-classification:						
	- Alimentary tract and metabolism	25	2.0% (0.608)	14.2% (0.486)	-3.3% (0.433)	-14.5% (0.633)	15.0% (0.163)
	- Blood and blood forming organs	8	1.6% (0.001)	-8.8% (<.001)	-0.7% (0.114)	-8.4% (0.051)	0.8% (0.602)
	- Cardiovascular system	9	-1.0% (0.655)	0.7% (0.931)	-0.9% (0.723)	10.6% (0.308)	3.3% (0.459)
	- Dermatological	9	1.8% (0.220)	-6.8% (0.366)	-1.9% (0.216)	-9.9% (0.429)	0.8% (0.853)
	- Genito urinary system and sex hormones	4	3.6% (<.001)	-0.1% (0.972)	-3.5% (<.001)	3.2% (0.618)	-1.2% (0.588)
	- Systemic hormonal preparations	0	n.a.	n.a.	n.a.	n.a.	n.a.
	- Anti-infective for systemic use	20	5.1% (0.006)	-42.8% (<.001)	-4.4% (0.020)	3.9% (0.765)	0.1% (0.981)
	- Antineoplastic and immunomodulating agents	0	n.a.	n.a.	n.a.	n.a.	n.a.

	- Musculo-skeletal system	2	-7.9% (<.001)	24.7% (<.001)	7.2% (<.001)	6.3% (0.543)	0.5% (0.898)
	- Nervous system	7	-1.1% (0.511)	1.6% (0.807)	0.1% (0.940)	1.5% (0.843)	0.8% (0.802)
	- Antiparasitic products, insecticides and repellents	4	1.1% (0.708)	7.9% (0.599)	2.3% (0.491)	-5.2% (0.799)	-5.4% (0.473)
	- Respiratory system	8	10.5% (<.001)	-16.8% (0.077)	-11.9% (<.001)	15.7% (0.250)	6.4% (0.185)
	- Sensory organs	16	0.8% (0.475)	25.3% (<.001)	-0.6% (0.579)	-10.3% (0.300)	1.7% (0.615)
	By TCM-classification:						
	- Internal medicine	36	1.2% (0.037)	3.3% (0.237)	-1.3% (0.021)	-4.6% (0.332)	4.3% (0.013)
	- Surgical	3	4.4% (<.001)	6.0% (0.239)	-5.3% (<.001)	5.2% (0.478)	1.0% (0.686)
	- Gynecological	2	-1.1% (0.021)	4.3% (0.077)	1.1% (0.031)	2.5% (0.539)	-0.7% (0.650)
	- Orthopedics	7	5.9% (<.001)	-4.9% (0.357)	-5.6% (<.001)	3.9% (0.663)	-2.2% (0.467)
	- Ear, nose and throat	1	8.8% (<.001)	-12.3% (0.026)	-9.0% (<.001)	0.7% (0.932)	0.2% (0.956)
	- Ophthalmology	0	n.a.	n.a.	n.a.	n.a.	n.a.

Part II. Drug Price Index – Laspeyres (DPI-L)

Facility	Drug category	Number of drugs included	Time, $\exp(\beta_1)$-1 (*p-value*)	1st-stage NEMP, $\exp(\beta_2)$-1 (*p-value*)	Time after 1st-stage NEMP, $\exp(\beta_3)$-1 (*p-value*)	2nd-stage NEMP, $\exp(\beta_4)$-1 (*p-value*)	Time after 2nd-stage NEMP, $\exp(\beta_4)$-1 (*p-value*)
County-wide	Overall	405	0.6% (0.108)	-5.1% (0.007)	-0.8% (0.041)	1.9% (0.500)	1.7% (0.086)
	- Essential drug	321	0.9% (0.011)	-2.2% (0.215)	-1.1% (0.005)	2.8% (0.364)	2.0% (0.070)
	- Non-essential drug	84	-0.6% (0.302)	-5.1% (0.014)	0.1% (0.896)	-1.4% (0.519)	1.4% (0.220)
	Western medicine	278	0.0% (0.948)	-5.6% (0.035)	-0.2% (0.650)	1.7% (0.642)	2.2% (0.100)
	- Essential drug	230	0.0% (0.948)	-5.6% (0.035)	-0.2% (0.650)	1.7% (0.642)	2.2% (0.100)
	- Non-essential drug	48	-0.7% (0.239)	-5.8% (0.009)	0.2% (0.792)	-1.7% (0.484)	1.4% (0.249)
	TCM	127	1.4% (<.001)	1.9% (0.330)	-1.6% (<.001)	0.3% (0.918)	1.1% (0.339)
	- Essential drug	91	1.4% (0.002)	1.8% (0.392)	-1.7% (<.001)	0.7% (0.837)	1.0% (0.420)
	- Non-essential drug	36	1.0% (0.003)	2.1% (0.196)	-1.0% (0.005)	-3.6% (0.191)	1.7% (0.083)
	By ATC-classification:						
	- Alimentary tract and metabolism	82	-0.1% (0.816)	-1.1% (0.689)	0.4% (0.483)	-3.6% (0.438)	4.4% (0.010)
	- Blood and blood forming organs	35	-0.8% (0.111)	-0.6% (0.802)	0.8% (0.097)	-1.9% (0.626)	3.5% (0.015)
	- Cardiovascular system	47	-0.8% (0.118)	-2.1% (0.377)	0.9% (0.082)	-3.6% (0.400)	4.2% (0.008)
	- Dermatological	35	0.1% (0.824)	-13.0% (<.001)	-0.2% (0.472)	2.3% (0.423)	1.9% (0.064)
	- Genito urinary system and sex hormones	20	-0.6% (0.210)	-12.1% (<.001)	0.2% (0.703)	1.8% (0.658)	1.5% (0.301)
	- Systemic hormonal preparations	9	-0.6% (0.076)	-13.5% (<.001)	0.3% (0.351)	1.9% (0.467)	1.9% (0.053)
	- Anti-infective for systemic use	54	0.5% (0.339)	-15.7% (<.001)	-0.7% (0.164)	-0.7% (0.847)	0.2% (0.876)
	- Antineoplastic and immunomodulating agents	6	-0.7% (0.178)	-11.7% (<.001)	0.6% (0.259)	4.2% (0.290)	-1.7% (0.222)
	- Musculo-skeletal system	8	-0.1% (0.734)	-16.4% (<.001)	-0.2% (0.648)	-0.8% (0.794)	0.3% (0.800)
	- Nervous system	39	0.5% (0.314)	1.6% (0.505)	0.5% (0.295)	-9.1% (0.029)	5.0% (0.001)

	- Antiparasitic products, insecticides and repellents	6	0.1% (0.894)	4.6% (0.162)	0.3% (0.639)	-8.7% (0.113)	4.3% (0.028)
	- Respiratory system	34	-0.1% (0.798)	0.2% (0.942)	0.9% (0.064)	-8.9% (0.029)	4.7% (0.002)
	- Sensory organs	51	0.5% (0.232)	-1.0% (0.635)	0.1% (0.799)	10.7% (0.005)	-0.9% (0.453)
	By TCM-classification:						
	- Internal medicine	99	-0.1% (0.721)	-2.2% (0.262)	0.6% (0.150)	9.6% (0.006)	-0.7% (0.510)
	- Surgical	8	-3.1% (0.105)	4.7% (0.624)	4.4% (0.031)	-10.2% (0.513)	4.3% (0.421)
	- Gynecological	3	-3.6% (0.060)	6.3% (0.504)	4.2% (0.035)	-12.1% (0.416)	4.5% (0.383)
	- Orthopedics	9	-3.6% (0.045)	3.7% (0.679)	4.6% (0.014)	-9.9% (0.493)	4.2% (0.406)
	- Ear, nose and throat	6	1.2% (0.376)	-3.5% (0.414)	-2.4% (0.169)	-3.0% (0.548)	2.2% (0.383)
	- Ophthalmology	2	0.7% (0.596)	-8.6% (0.086)	-1.7% (0.303)	1.6% (0.780)	1.4% (0.609)
County hospitals	Overall	383	0.5% (0.160)	-5.7% (0.004)	-0.8% (0.039)	-0.5% (0.860)	2.5% (0.017)
	- Essential drug	300	0.9% (0.008)	-3.0% (0.086)	-1.2% (0.001)	-0.2% (0.940)	3.0% (0.007)
	- Non-essential drug	83	-0.7% (0.270)	-5.1% (0.021)	0.2% (0.835)	-1.5% (0.542)	1.4% (0.240)
	Western medicine	270	-0.1% (0.865)	-5.5% (0.048)	-0.2% (0.784)	0.3% (0.932)	2.2% (0.109)
	- Essential drug	223	0.6% (0.247)	-5.4% (0.065)	-0.8% (0.192)	1.3% (0.765)	2.5% (0.114)
	- Non-essential drug	47	-0.8% (0.239)	-5.7% (0.015)	0.2% (0.766)	-1.7% (0.500)	1.4% (0.259)
	TCM	113	1.1% (0.126)	3.3% (0.351)	-1.6% (0.037)	-7.0% (0.196)	4.3% (0.027)
	- Essential drug	77	1.1% (0.151)	3.4% (0.384)	-1.6% (0.048)	-7.7% (0.193)	4.6% (0.029)
	- Non-essential drug	36	0.8% (0.008)	2.2% (0.121)	-0.9% (0.002)	-1.1% (0.603)	0.9% (0.265)
	By ATC-classification:						
	- Alimentary tract and metabolism	80	0.7% (0.614)	-5.9% (0.166)	-1.8% (0.268)	-3.0% (0.536)	2.2% (0.373)
	- Blood and blood forming organs	35	0.2% (0.740)	-1.4% (0.614)	-0.5% (0.326)	-5.8% (0.191)	3.4% (0.029)
	- Cardiovascular system	46	-0.7% (0.219)	-1.2% (0.681)	0.2% (0.722)	-5.9% (0.234)	3.5% (0.049)
	- Dermatological	34	-0.4% (0.367)	-2.7% (0.283)	0.0% (0.966)	-5.5% (0.173)	3.3% (0.023)
	- Genito urinary system and sex hormones	20	1.1% (0.183)	1.5% (0.719)	0.3% (0.702)	-23.2% (0.003)	13.9% (<.001)
	- Systemic hormonal preparations	9	-0.6% (0.708)	28.5% (0.003)	0.4% (0.834)	-9.0% (0.511)	6.5% (0.177)
	- Anti-infective for systemic use	52	0.4% (0.581)	0.3% (0.939)	0.7% (0.334)	-21.9% (0.003)	13.0% (<.001)
	- Antineoplastic and immunomodulating agents	6	-0.2% (0.784)	0.8% (0.862)	0.3% (0.738)	2.5% (0.708)	0.2% (0.928)
	- Musculo-skeletal system	8	-1.0% (0.079)	-4.5% (0.125)	0.9% (0.120)	1.0% (0.840)	0.8% (0.637)
	- Nervous system	38	-0.8% (0.347)	-0.7% (0.860)	0.7% (0.403)	2.0% (0.744)	0.3% (0.891)
	- Antiparasitic products, insecticides and repellents	5	0.5% (0.827)	-5.9% (0.629)	4.5% (0.107)	-32.8% (0.055)	-5.3% (0.383)
	- Respiratory system	34	0.1% (0.965)	-5.8% (0.638)	4.6% (0.105)	-33.9% (0.045)	-5.1% (0.396)
	- Sensory organs	48	0.0% (0.989)	-7.3% (0.532)	4.6% (0.090)	-31.0% (0.054)	-4.9% (0.400)
	By TCM-classification:						
	- Internal medicine	89	0.0% (0.993)	-4.5% (0.537)	-0.1% (0.949)	9.5% (0.427)	-0.2% (0.959)
	- Surgical	7	0.1% (0.949)	-7.1% (0.417)	-0.2% (0.911)	8.1% (0.564)	0.6% (0.894)
	- Gynecological	1	-0.2% (0.840)	6.9% (0.276)	1.1% (0.365)	-0.9% (0.928)	2.6% (0.474)
	- Orthopedics	8	-0.1% (0.940)	1.8% (0.779)	0.8% (0.556)	3.0% (0.781)	0.6% (0.876)
	- Ear, nose and throat	6	-0.8% (0.470)	5.4% (0.365)	1.5% (0.201)	-1.3% (0.898)	2.5% (0.464)
	- Ophthalmology	2	1.5% (0.001)	-7.9% (<.001)	-1.8% (<.001)	4.1% (0.246)	0.7% (0.548)

THCs	Overall - Essential drug	117	3.2% (<.001)	-12.0% (0.003)	-3.5% (<.001)	-2.1% (0.667)	5.4% (0.005)
	Western medicine - Essential drug	68	3.3% (0.008)	-18.4% (0.004)	-3.7% (0.005)	-3.1% (0.708)	8.2% (0.009)
	TCM - Essential drug	49	3.5% (<.001)	-5.2% (0.044)	-3.6% (<.001)	1.0% (0.806)	0.9% (0.517)
	By ATC-classification:						
	- Alimentary tract and metabolism	25	0.6% (0.030)	-6.7% (<.001)	-0.9% (0.002)	4.2% (0.091)	0.4% (0.625)
	- Blood and blood forming organs	8	0.8% (0.060)	-8.8% (<.001)	-1.2% (0.006)	3.3% (0.331)	0.9% (0.448)
	- Cardiovascular system	9	3.0% (<.001)	-5.8% (0.082)	-3.2% (<.001)	1.2% (0.810)	1.2% (0.505)
	- Dermatological	9	2.5% (0.002)	-7.9% (0.046)	-2.8% (<.001)	1.8% (0.782)	3.1% (0.172)
	- Genito urinary system and sex hormones	4	2.3% (<.001)	-7.2% (0.023)	-2.6% (<.001)	0.9% (0.841)	1.2% (0.463)
	- Systemic hormonal preparations	0	3.4% (<.001)	0.1% (0.971)	-2.8% (0.005)	-3.5% (0.450)	-1.5% (0.431)
	- Anti-infective for systemic use	20	-1.7% (0.028)	1.4% (0.694)	1.9% (0.022)	-1.6% (0.750)	0.3% (0.866)
	- Antineoplastic and immunomodulating agents	0	2.8% (0.002)	-2.7% (0.452)	-2.4% (0.017)	-3.6% (0.414)	-1.2% (0.489)
	- Musculo-skeletal system	2	0.1% (0.919)	-2.3% (0.722)	-0.1% (0.960)	-4.8% (0.654)	0.0% (0.999)
	- Nervous system	7	-0.5% (0.664)	-3.5% (0.564)	0.4% (0.711)	-4.8% (0.638)	0.1% (0.971)
	- Antiparasitic products, insecticides and repellents	4	-0.5% (0.664)	-3.5% (0.564)	0.4% (0.711)	-4.8% (0.638)	0.1% (0.971)
	- Respiratory system	8	2.4% (<.001)	-0.8% (0.691)	-2.3% (<.001)	-0.5% (0.812)	-0.5% (0.604)
	- Sensory organs	16	2.1% (0.016)	1.4% (0.715)	-2.3% (0.013)	-1.9% (0.722)	-0.6% (0.754)
	By TCM-classification:						
	- Internal medicine	36	0.8% (0.746)	-5.4% (0.671)	-0.2% (0.945)	3.4% (0.870)	-0.5% (0.941)
	- Surgical	3	0.4% (0.846)	-7.3% (0.518)	0.0% (0.999)	1.9% (0.916)	-0.1% (0.984)
	- Gynecological	2	0.2% (0.933)	-6.7% (0.577)	0.3% (0.913)	2.9% (0.881)	-0.4% (0.955)
	- Orthopedics	7	0.7% (0.066)	-1.6% (0.409)	-0.9% (0.029)	0.8% (0.790)	0.2% (0.876)
	- Ear, nose and throat	1	0.3% (0.469)	-3.7% (0.063)	-0.6% (0.141)	0.7% (0.829)	0.3% (0.788)
	- Ophthalmology	0	0.1% (0.837)	-2.9% (0.106)	-0.4% (0.309)	0.6% (0.840)	0.3% (0.782)

Note: Segmented linear regression model was built with two interruption points: $Y_t = \beta_0 + \beta_1 T + \beta_2 X_{1\_t} + \beta_3 T X_{1\_t} + \beta_4 X_{2\_t} + \beta_5 T X_{2\_t} + \varepsilon_t$, Coefficient β0 estimates the baseline level of outcome; β1 estimates the time trend of outcome in the pre-policy period; β2 and β4 estimate the immediate changes in level after the first- and second-stage policy; and β3 and β5¬ estimate the sustained change in trend after the first- and second-stage policy; ATC, Anatomical Therapeutic Chemical; TCM, Traditional Chinese Medicine; NEMP, National Essential Medicines Policy; [*], P<0.05; [**], P<0.01; [***], P<0.001

Appendix 17. Sensitivity analysis on drug price index after adjusting for inflation

Outcome 1: Adjusted Retail Price

■ overall (DPI-F) ▲ essential medicines (DPI-F) ♦ non-essential medicines (DPI-F)

□ overall (DPI-L) △ essential medicines (DPI-L) ◊ non-essential medicines (DPI-L)

▬ model fitted line for DPI-F

Pre-NEMP 1st stage NEMP 2nd stage NEMP

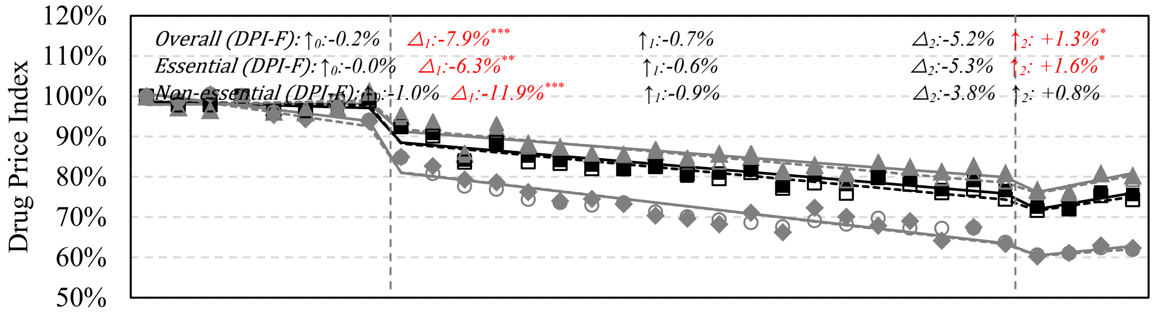

(a) all facilities

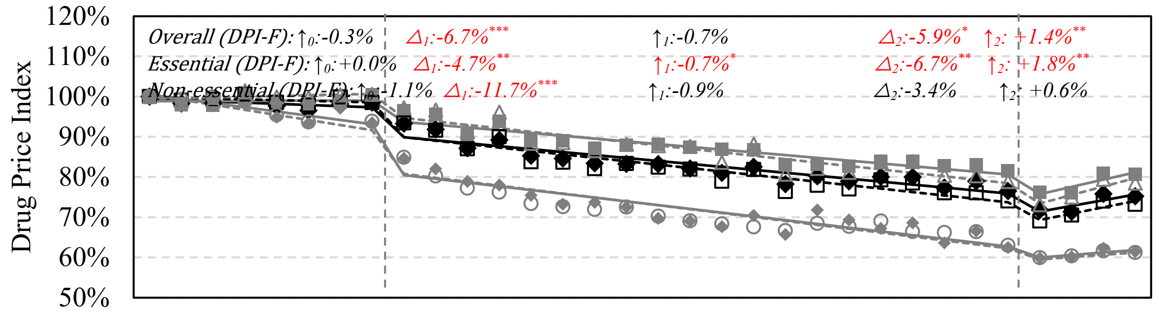

(b) county hospital

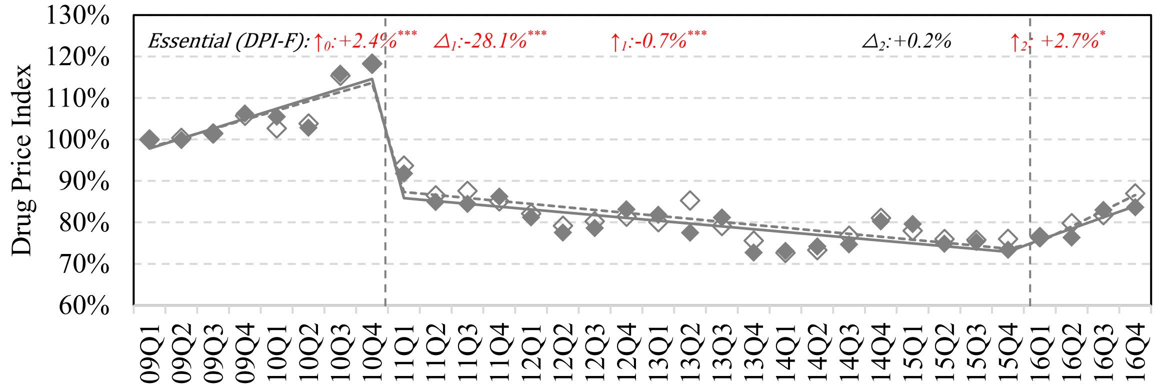

(c) township healthcare centre

$\uparrow_0$: sustained change per observation period before first-phase NEMP (β_1); $\triangle_1$: immediate price change% after first-phase NEMP (exp(β_3) in ITSA model); $\uparrow_1$: sustained price change% per observation period after first-phase NEMP (exp($\beta_1+\beta_3$)); $\triangle_2$: immediate price change% after second-phase NEMP (exp(β_4)); $\uparrow_2$: sustained price change% per observation period after second-phase NEMP (exp($\beta_1+\beta_3+\beta_5$)); ITSA, interrupted time-series analysis; NEMP, National Essential Medicines Policy; *, P<0.05; **, P<0.01; ***, P<0.001

Outcome 2: Adjusted Wholesale Price

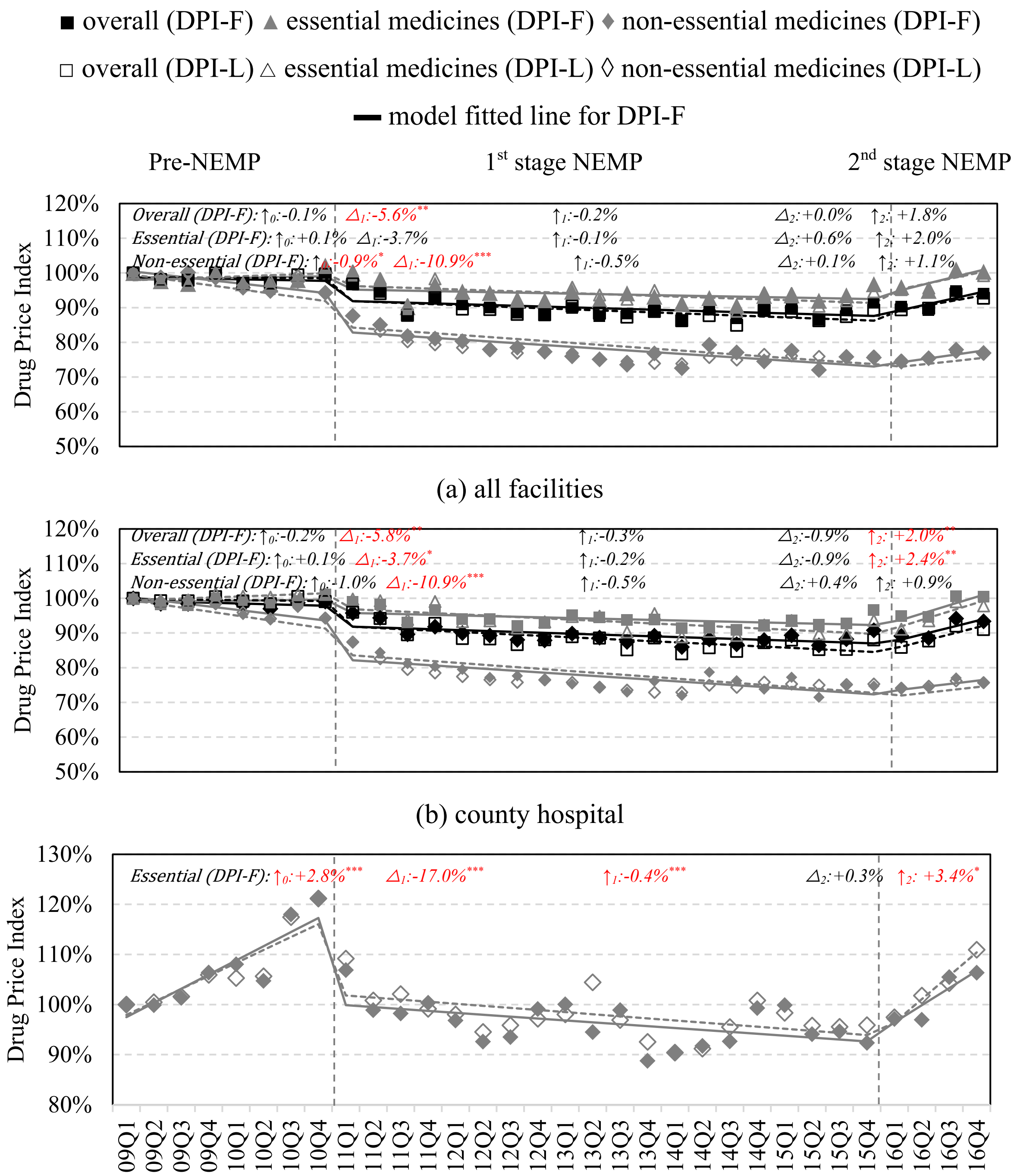

(c) township healthcare centre

$\uparrow_0$: sustained change per observation period before first-phase NEMP (β_1); $\triangle_1$: immediate price change% after first-phase NEMP ($\exp(\beta_3)$ in ITSA model); $\uparrow_1$: sustained price change% per observation period after first-phase NEMP ($\exp(\beta_1+\beta_3)$); $\triangle_2$: immediate price change% after second-phase NEMP ($\exp(\beta_4)$); $\uparrow_2$: sustained price change% per observation period after second-phase NEMP ($\exp(\beta_1+\beta_3+\beta_5)$); ITSA, interrupted time-series analysis; NEMP, National Essential Medicines Policy; *, P<0.05; **, P<0.01; ***, P<0.001
